# Supporting Ultra Poor People with Rehabilitation and Therapy among families of children with Cerebral Palsy in rural Bangladesh (SUPPORT CP): Protocol of a randomised controlled trial

**DOI:** 10.1371/journal.pone.0261148

**Published:** 2021-12-31

**Authors:** Mahmudul Hassan Al Imam, Israt Jahan, Mohammad Muhit, Manik Chandra Das, Rosalie Power, Arifuzzaman Khan, Delwar Akbar, Nadia Badawi, Gulam Khandaker

**Affiliations:** 1 CSF Global, Dhaka, Bangladesh; 2 Asian Institute of Disability and Development (AIDD), University of South Asia, Dhaka, Bangladesh; 3 School of Health, Medical and Applied Sciences, Central Queensland University, Rockhampton, Queensland, Australia; 4 Central Queensland Public Health Unit, Central Queensland Hospital and Health Service, Rockhampton, Queensland, Australia; 5 Translational Health Research Institute (THRI), Western Sydney University, Campbelltown, New South Wales, Australia; 6 School of Public Health, The University of Queensland, Brisbane, Australia; 7 School of Business and Law, Central Queensland University, Rockhampton, Queensland, Australia; 8 Cerebral Palsy Alliance, Sydney Medical School, The University of Sydney, Camperdown, New South Wales, Australia; 9 Grace Centre for Newborn Intensive Care, Sydney Children’s Hospital Network, Westmead, New South Wales, Australia; 10 Discipline of Child and Adolescent Health, Sydney Medical School, The University of Sydney, Sydney, Australia; Prince Sattam Bin Abdulaziz University, College of Applied Medical Sciences, SAUDI ARABIA

## Abstract

**Introduction:**

Poverty is a key contributor to delayed diagnosis and limited access to early intervention and rehabilitation for children with cerebral palsy (CP) in rural Bangladesh. 97% of families of children with CP live below the poverty line in Bangladesh. Therefore, in low-and middle-income countries (LMICs), efforts to improve outcomes for children with CP (including health-related quality of life, motor function, communication, and nutritional attainments) should also include measures to improve family economic and social capital. We propose a randomised controlled trial (RCT) to evaluate the effectiveness of an integrated microfinance/livelihood and community-based rehabilitation (IMCBR) program for ultra-poor families of children with CP in rural Bangladesh.

**Material and methods:**

This will be a cluster RCT comparing three arms: (a) integrated microfinance/livelihood and community-based rehabilitation (IMCBR); (b) community-based rehabilitation (CBR) alone; and (c) care-as-usual (i.e. no intervention). Seven clusters will be recruited within each arm. Each cluster will consist of 10 child-caregiver dyads totalling 21 clusters with 210 dyads. Parents recruited in the IMCBR arm will take part in a microfinance/livelihood program and Parent Training Module (PTM), their children with CP will take part in a Goal Directed Training (GDT) program. The programs will be facilitated by specially trained Community Rehabilitation Officers. The CBR arm includes the same PTM and GDT interventions excluding the microfinance/livelihood program. The care-as-usual arm will be provided with information about early intervention and rehabilitation. The assessors will be blinded to group allocation. The duration of the intervention will be 12 months; outcomes will be measured at baseline, 6 months, 12 months, and 18 months.

**Conclusion:**

This will be the first RCT of an integrated microfinance/livelihood and CBR program for children with CP in LMIC settings. Evidence from the study could transform approaches to improving wellbeing of children with CP and their ultra-poor families.

## Introduction

Cerebral palsy (CP) is a group of non-progressive neurological disorders caused by damage to the developing brain [1]. The prevalence and severity of CP are considerably higher in low- and middle-income countries (LMICs) compared with high-income countries (HICs) [2–4] and diagnosis is likely to be delayed [3]. Early diagnosis of children with CP and access to evidence-based early interventions such as community-based rehabilitation (CBR) are key to improving the long-term health-related quality of life (HRQoL) [5], motor function [6], cognitive [7], and other health outcomes in children with CP. However, the majority of such evidence is from HICs [8, 9]. Randomised controlled trials (RCTs) testing the effectiveness of CBR programs for children with CP in LMICs are relatively scarce. Moreover, these interventions rarely consider issues pertinent in the lives of children with CP and their ultra-poor families in LMICs.

In LMICs, many families of children with CP are ultra-poor, which contributes to poor health care access, delayed diagnosis, delayed intervention, overall poor health and wellbeing, and long-term reduced effectiveness of rehabilitation therapies [3, 10–15]. Our last 16 years of research in rural Bangladesh, which led to the development of the Bangladesh CP Register (BCPR—first ongoing population-based CP register in LMICs) [16], confirms that diagnosis of CP is delayed [17] and there is limited or no access to evidence-based rehabilitation programs in rural Bangladesh [18]. The average age at diagnosis of CP in Bangladesh is 5 years compared to 1.5 years in HICs [2, 3]. We also found that even when rehabilitation programs were available, access to care was negatively impacted by poverty [3, 14]. In Bangladesh, 97% of families of children with CP live below the poverty line [3]. Moreover, people with disability and their families are often excluded from economic activities [19]. These families struggle to meet basic needs and their child’s rehabilitation often does not feature high on the agenda. Therefore, an integrated approach combining the physical rehabilitation of children with CP and the economic empowerment of their family is required for tangible long-term improvements.

Microfinance/livelihood support is an effective tool for improving economic, human (including non-cognitive skills), and social capital of disadvantaged people in LMICs particularly vulnerable groups such as women and children [20]. Microfinance/livelihood support programs can improve health by increasing financial access and service utilisation. Combining microfinance with health interventions has yielded promising results in the fields of HIV, malaria, and breastfeeding in Africa [21].

In addition, non-experimental and quasi-experimental studies testing the effectiveness of integrated health and economic interventions report significant improvements in reproductive and child health, nutrition, and immunisation [22, 23]. Non‐cognitive skills or soft skills are considered as important predictors of socio‐economic outcomes in life [24]. For instance, the training on soft skills (e.g. personal initiative) aided the development of small-scale businesses in the African context [25, 26]. Moreover, the interaction between diversified groups in society reduces prejudice and promotes inter‐group cooperation [27–29].

To be effective, interventions need to be tailored according to the needs of the target population [30]. Influential works by Professor Sir Michael Marmot, Chair of the World Health Organization (WHO) Commission on Social Determinants of Health, and others have demonstrated that socioeconomic factors are important determinants of health [31]. Even in HICs like the United Kingdom, the average life expectancy in poorer areas of Glasgow is about 20 years shorter than that for the rest of the country [32]. This gap can be explained as a direct result of poverty and related social disadvantage. Tangible improvements in the overall health status of people living in poverty can only be achieved by focusing on improving both health and economic/social capital. However, to our knowledge, no studies have examined the effectiveness of an integrated health and economic approach for children with CP and their families in LMICs.

In this paper, we describe an RCT protocol to test the effectiveness of an integrated microfinance/livelihood and CBR program (IMCBR) targeted to children with CP and their parents from ultra-poor families in rural Bangladesh. If the proposed integrated program is proved to be scientifically effective in improving the health and wellbeing of children with CP and their caregivers, the implementing NGO partner plans to scale up the program using existing connections with NGOs and microfinance organisations in Bangladesh, and in other LMICs where CSF Global is research active (e.g., Nepal, Indonesia, and Ghana).

## Materials and methods

### Study aims and objectives

This study aims to test the effectiveness of an “Integrated Microfinance/livelihood and CBR program” (IMCBR) among children with CP and their primary caregivers from ultra-poor families in rural Bangladesh. The program aims to improve the HRQoL, motor function, communication and nutritional status of children with CP; mental health, HRQoL and social capital of their parents; and socio-economic status (SES) and food security of their families.

Our specific objectives are;

1. To conduct an RCT with three parallel arms comparing (a) IMCBR, (b) CBR alone, and (c) care-as-usual (i.e. no intervention).
2. To measure the effectiveness of IMCBR in improving the HRQoL, motor function, communication, and nutritional status of children with CP from ultra-poor families living in rural Bangladesh.
3. To measure the effectiveness of IMCBR in improving mental health, HRQoL, and social capital of parents of children with CP living in rural Bangladesh.
4. To measure the effectiveness of IMCBR in improving the SES of ultra-poor families of children with CP living in rural Bangladesh.

### Hypothesis

We hypothesise that compared to care-as-usual and CBR alone, the IMCBR program will be more effective in improving HRQoL, motor function, communication, and nutritional outcomes of children with CP from ultra-poor families; and the mental health, HRQoL, and social capital of their primary caregivers; and overall improvement in SES of the ultra-poor families of children with CP in rural Bangladesh.

### Overview of the study design

**Fig 1:**
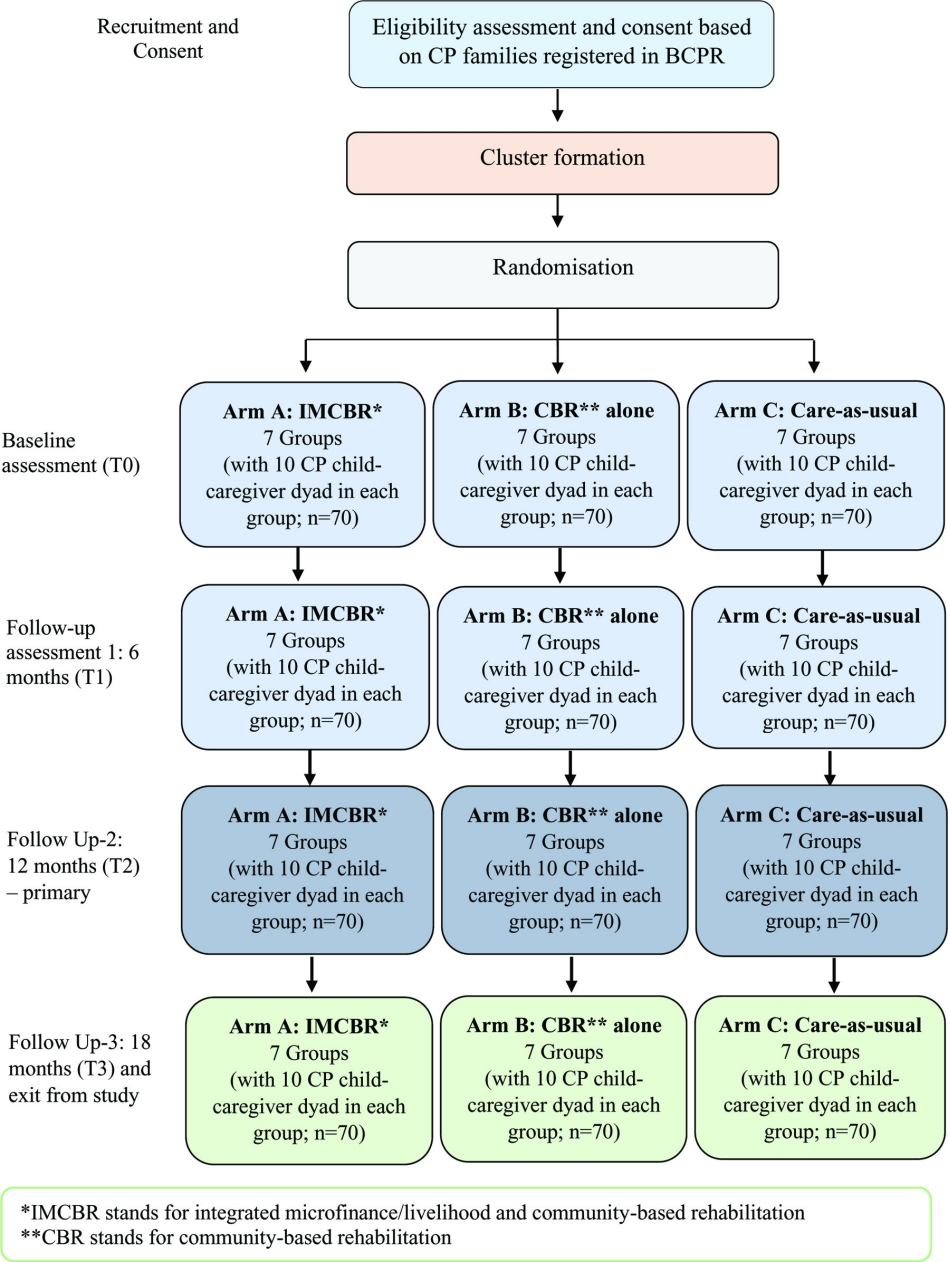
Study design (consolidated standards of reporting trials diagram).

This will be a cluster randomised controlled trial comprising three arms. The unit of randomisation will be a cluster. This trial will incorporate two intervention arms in order to compare the usual rehabilitation services with a new integrated microfinance/livelihood and CBR services. Clusters randomised to intervention arms of the trial (i.e. IMCBR and CBR arms) will receive interventions following the protocol outlined in later sections. The interventions will be provided to dyads consisting of children with CP and their primary caregivers (Fig 1). Whereas clusters randomised to care-as-usual arm of the trial will not receive any active intervention.

### Study location

The study will be implemented in Shahjadpur sub-district (~324.15 sq. km) of Sirajganj district located in the northern part of Bangladesh. The study site is comprised of ~70,998 households with a total population of ~561,076 (child population aged 0–18 years ~226,114), and 12,117 live births per annum [3]. The study site constitutes a complex socio-demographic locale including urban, rural, and hard-to-reach areas and represents the overall socio-demographic and economic characteristics of rural areas in Bangladesh [33, 34].

### Recruitment of study participants

**Fig 2:**
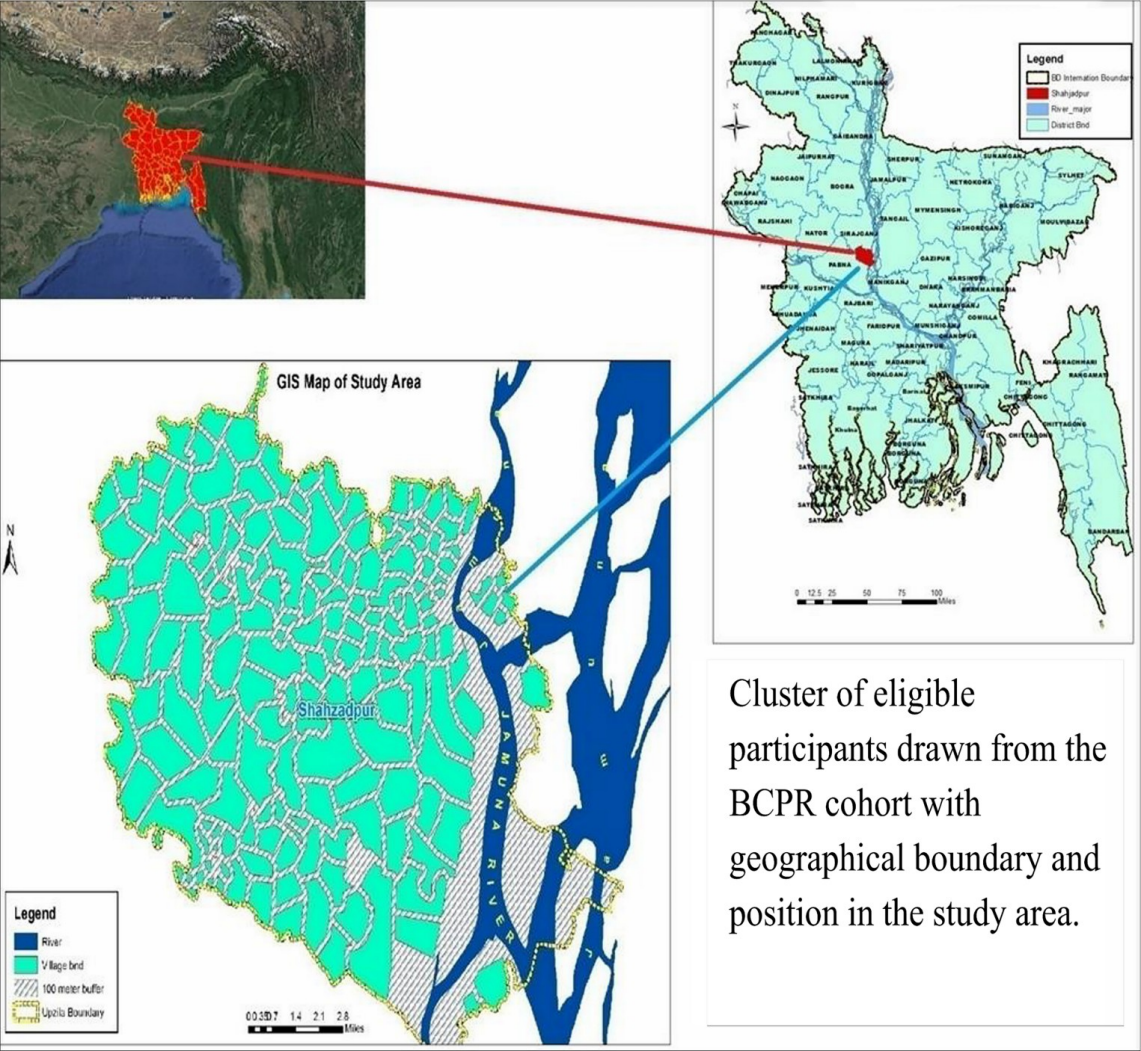
Geographic Information System (GIS) map of the study area and eligible clusters. This map was produced by the authors using ArcGIS Desktop 10.8 software.

The study will utilise the BCPR as a sampling frame for participant recruitment. The BCPR is an ongoing surveillance of children with CP commenced in 2015 [3], and is currently being operated in four districts of Bangladesh. Between 2015 and 2019, 1125 children with CP have been registered into the BCPR from Shahjadpur (i.e. the study site) (study area–Fig 2). As part of this RCT, dyads of children with CP and their primary caregivers who meet the inclusion criteria will be recruited and assigned to different arms of the study.

### Inclusion/Exclusion criteria

Participants will be considered eligible for participation based on the following criteria:

1. Children with CP aged ≤5 years, from an ultra-poor family (as classified by the World Bank i.e. per day per capita income <1.90 USD; [35]) and registered in the BCPR. The BCPR registers children with CP following the case definition adopted from the Surveillance of CP in Europe (SCPE) and the Australian CP Register (ACPR) [3].
2. Primary caregiver (e.g. parent, sibling, grandparent of the child with CP)
3. Primary caregiver has the capacity to give informed consent and is willing to take part in the study including microfinance/livelihood arm along with their child with CP.

Participants will be considered ineligible for participation based on the following criteria:

1. Currently in receipt of microfinance/livelihood support from another source.
2. Currently participating in any other clinical trial or intervention program.

The BCPR findings show that 34.2% of the children registered are aged <5 years and 97% of the families are ultra-poor [3]. Considering the number of registrants from Shahjadpur (i.e.~1125), there are ~373 children eligible to participate in the study. Therefore, recruitment of 210 children and their primary caregiver in the trial (<60% of the available pool) is feasible. Sociodemographic, economic, and health data of these children and their families are already recorded in the BCPR allowing quick identification and recruitment. All families enrolled in the BCPR have also been mapped using Geographic Information System (GIS) (Fig 2).

### Sample size calculation

The sample size for this cluster RCT has been computed based on methods described in Donner et al. [36]. We made several assumptions from the existing knowledge about the study population while calculating the sample size for this cluster RCT. Specifically, our sample size calculation is balanced between a standard statistical power and a pragmatic study setting. Based on our pilot data and existing literature we predict a 35% improvement of HRQoL in the IMCBR group, 20% improvement in the CBR alone group, and 5% improvement in the care-as-usual group [9]. A homogeneous study population will allow us to balance randomisation considering intra-cluster correlation of 0.5 and coefficient of variation in cluster size of 0.5.

Based on the calculation, we will recruit seven clusters in each arm (allocating 1:1:1), and each cluster will consist of 10 children with CP- primary caregivers dyads totalling 21 clusters of 210 dyads. With a sample size of 210 dyads, this study will have 80% statistical power to detect these effects (two-sided α-value = 0.05). Power calculation took into account up to 20% sample attrition by the end of the trial.

### Cluster formation and randomisation

The study will include 21 clusters randomised to three arms (7 clusters each) and allocated by a 1:1:1 ratio. While deciding the allocation ratio of 1:1:1 we considered the number of children with CP available in the BCPR sampling frame as well as the high mortality rate among children with CP below five years of age [37]. Each cluster will come from a ‘Mouza’, the smallest public administrative unit in Bangladesh comprising of approximately five villages (~8,250 people) and will include 10 CP child-primary caregiver dyads. In order to minimise ‘contamination’ of the intervention types, clusters will be separated from each other by buffering areas comprising villages not taking part in the study. Cluster margins will be configured so that they align with natural divisions that separate residents in the community (e.g. rivers). The randomisation process will be executed following the standard process.

### Blinding

The interventions will be open to participants and investigators, however, the outcome assessments will be observer blinded. An independent team, informed on the purpose and importance of blinding for this study, will conduct outcome assessments. The assessors will be masked/blinded about the interventions and the outcome assessment questionnaire will be designed in a manner that does not disclose the intervention.

### Intervention

#### Arm A- Integrated Microfinance/livelihood and Community-Based Rehabilitation (IMCBR)

Participants randomised to IMCBR arm will be supported to create microfinance/livelihood groups (10 CP child-primary caregiver dyads per group). The groups will be formed voluntarily along geographical boundaries to facilitate participation, retention, and meeting logistics. Each cluster will meet weekly to discuss microfinance/livelihood activities (e.g. weekly credit collection and troubleshooting) (90 minutes) and for CBR with children with CP comprising early intervention and primary caregiver’s education (90 minutes).

*A*.*1 Microfinance/livelihood program details*. Group meetings will be organised with members of each cluster to discuss (i) details of the program, (ii) potential benefits and challenges of participation in the program, and (iii) motivations for participation. Participants of each cluster can then apply for a loan/livelihood support; a minimum 10% deposit of the requested loan/livelihood support amount in the form of savings is required and is admissible immediately after cluster formation. Once the application is completed, loan approval and disbursement of the loan will occur approximately within one week. The loan disbursement and collection of repayment will be managed by a local microfinance organisation. However, the funds required for the loan will be reimbursed from the study budget at the end of loan disbursement.

Amount, return cycle of loan and investment areas: Each of the ultra-poor families will receive a loan/livelihood support amounting/equivalent to ~100–300 AUD at a 12% flat interest rate. The return cycle will be one year with a weekly repayment schedule. Common investment areas for the ultra-poor loan will be for goat or cattle rearing, seeds for agriculture, home-based weaving, and handicraft business [38].

*A*.*2 CBR*. There will be two major components of the CBR program, which will occur during cluster meetings following the microfinance/livelihood portion.

a. Goal Directed Training (GDT): Community-based GDT focused on motor learning will be conducted with children with CP and their primary caregivers. GDT is an activity-based approach to therapy where meaningful, client-selected (i.e. caregivers of children with CP) goals are used to provide opportunities for problem solving and to indirectly drive the movements required to successfully meet task demands [39]. Evidence from a meta-analysis showed that GDT based interventions are highly effective and should be the gold standard treatment for CP [40]. In this study, GDT will be delivered by child’s primary caregiver (participating parent). There are less than 10 rehabilitation professionals per 1 million population in Bangladesh and the majority of them are located in urban cities [41]. In absence of adequately trained professionals, parents-led early intervention strategies have been found very effective and been proposed in earlier studies including RCTs [42]. Parent training is vital as they are the only person who can identify a child’s self-initiated attempts and therefore can practice active motor tasks [43].

There will be four components of GDT;

1. Goal selection: The CBR session will start with realistic and appropriately time-framed goals set up in collaboration with parents. Goals are basically some specific tasks that are needed for everyday life and which children with CP find challenging. These tasks could be gross motor, self-care, communication, play, or school-based activities.
2. Assessment: A detailed assessment will then be carried out to identify facilitating and limiting factors in achieving the goals. This will involve assessing the physical requirements of the selected task, the resources and equipment that are needed, and the settings in which the task is done. Both goal selection and assessment session will be facilitated by an experienced physiotherapist.
3. Intervention: Weekly group therapy sessions will be conducted by two Community Rehabilitation Officer (CROs). The CROs hold a certificate level course on basic CBR techniques and have five years of experience in community-based management of children with CP. The CROs will guide parents/primary caregivers to scaffold the selected motor tasks, so that the child could actively complete at least a part of the task. Parents will be encouraged to use their knowledge of their child’s play preferences to elicit self-generated motor activity. Modified constraint induced movement therapy and/or bimanual training will be used when asymmetrical hand function is evident. CROs will also assist parents to set up a motor-enriched play environment at home to promote child’s self-generated movements, exploration, and task success. Parents will be advised to practice through joyful play until the main goal is achieved.
4. Outcome evaluation: CROs will evaluate the compliance to the intervention by weekly follow-up phone calls weekly and monthly home visits. The attendance to group sessions and compliance to intervention will be monitored daily by a physiotherapist to ensure adherence to intervention. The extent to which a child’s goals are achieved will be monitored and recorded.

*b*. *Parents Training Module (PTM)*: Primary caregivers will participate in PTM to learn basic therapeutically correct skills for the day-to-day care and support of their child with CP embedded in the principles of GDT. This study will follow the PTM ‘Getting to know cerebral palsy’ which includes 10 modules and covers the following topics; introduction to CP, evaluating your child, positioning and carrying, communication, everyday activities, feeding your child, play, disability in your local community, running your own parent support group, and assistive devices and resources [44].

Specially trained CROs will facilitate each of the cluster meetings (both microfinance/livelihood and CBR activities). The CROs will facilitate microfinance/livelihood discussions and lead the GDT and PTM sessions with an aim to upskill primary caregivers so that they can continue to deliver GDT independently at home. Prior to implementation of the RCT, CROs will take part in a 5-day training by a Research Physiotherapist. The CRO training will cover the following areas; (i) socio-cultural considerations in working with primary caregivers of children with CP, (ii) introduction to microfinance/livelihood support program management, (iii) developmental milestones and development in children with CP, (iv) therapeutic principles, (v) GDT, (vi) activity focused therapies, (vii) basic speech development strategies, (viii) contraindications of therapies, (ix) research ethics, and (x) child rights.

#### Arm B- CBR alone

Participants from clusters randomised to this arm will attend a weekly rehabilitation session at a local focal point (preferably one of the group members’ homes). Each session will last for 90 minutes and will be identical to the CBR component of IMCBR (discussed above); however, the microfinance/livelihood component will not be provided.

#### Arm C- care-as-usual (i.e. no intervention)

This group will not receive any active intervention. Once children with CP are identified and randomised into clusters, the ‘care-as-usual’ participants will be provided with basic education on early intervention and rehabilitation and will be encouraged to access healthcare via usual routes, which typically include treatment in government hospitals. The proposed intervention schedules for all three arms have been summarised in S1 Table.

### Concurrent interventions

Children with CP from all three study arms will be able to continue accessing need-based medical and therapy support from other sources as per their family’s preferences. Frequency and duration of access to local medical/therapy services will be recorded during follow-up assessments and included in analysis.

### Outcome measures

The following outcomes will be measured at baseline, 6 months, 12 months, and 18 months.

### Primary outcomes

1. Health-related quality of life (HRQoL) of children with CP

*TNO-AZL Preschool children Quality of Life (TAPQOL)*: The TAPQOL is a validated tool designed to measure the parent perceived (i.e. proxy-reported) HRQoL of preschool children [45, 46]. The multidimensional instrument covers four domains; (a) Physical functioning, (b) Social functioning, (c) Cognitive functioning, and (d) Emotional functioning. The tool contains 43 items covering 12 scales (the number of items per scale ranges from 3 to 7), with higher scores indicating better HRQoL.

### Secondary and exploratory outcomes

1. Motor function of children with CP

*Gross Motor Function Measure (GMFM)-66*: The GMFM-66 is designed to assess the gross motor function in children with CP and measure changes over time. It is a valid and reliable tool comprising 66 items, subset of the 88 items included in original GMFM-88 developed based on the Rasch analysis to define the “gross motor function” of children with CP [47, 48]. The 66 activities in GMFM-66 cover five dimensions; (a) lying and rolling, (b) sitting, (c) crawling and sitting, (d) standing, and (e) walking, running and jumping.

*Gross Motor Function Classification System (GMFCS)*: The GMFCS is a five-level classification of functional motor abilities of children with CP. The Level I indicates minimal functional deficits among children with CP, whereas the level V indicates the highest level of functional deficits among children with CP [49].

*Classification of CP based on motor function and topographical distribution*: The predominant motor type i.e. spastic CP and non-spastic CP (dyskinesia, ataxia and hypotonia) and topographical distribution i.e. monoplegia, hemiplegia, diplegia, triplegia, and quadriplegia will be assessed following the BCPR protocol [3].

2. Communication function of children with CP

*Communication Function Classification System (CFCS)*: The CFCS is a validated tool to categorise children’s communication skills into five mutually exclusive levels of everyday communicative function. Classifications are made according to the descriptions of the levels and the distinctions between them [50, 51].

3. Nutritional status of children with CP

Anthropometric measurements will be taken using standard guidelines of WHO and will be analysed to assess the nutritional status of children. The following measurements will be collected at each assessment; (a) weight in kilograms, (b) height in centimetre (cm), (c) mid-upper arm circumference in cm, (d) skin-fold thickness in millimetre, and (e) head-circumference in cm.

4. Mental health of primary caregivers of children with CP

*Depression*, *Anxiety*, *Stress Scale–Short Form -21 (DASS-21)*: The DASS-21 is the shortened version of the DASS to assess symptoms of depression, anxiety and stress among adults. There are 21 items in this scale with four response options: 0 “Did not apply to me at all–Never”, 1 “Applied to me to some degree, or some of the time–Sometimes”, 2 “Applied to me to a considerable degree, or a good part of time–Often”, and 3 “Applied to me very much, or most of the time–Almost always” [52]. The Bengali version of the DASS-21 was previously validated to measure depression, anxiety and stress level among adults [53].

5. Health-related quality of life (HRQoL) of primary caregivers of children with CP

*Short Form 12-Version 2 (SF 12v2)*: The SF-12 is a validated tool to measure self-reported HRQoL of adults [54]. The tool has been widely used and has been translated into Bengali. In this study, we will use the reliable and validated Bengali version of the original SF-12 i.e. SF 12V2 [55]. The tool contains 12 questions covering eight domains; (a) physical functioning, (b) role physical, (c) bodily pain, (d) general health (e) vitality, (f) social functioning, (g) role emotional, and (h) mental health.

6. Social capital of primary caregivers of children with CP

*Short version of Adapted Social Capital Assessment Tool (SASCAT)*: The tool measures structural and cognitive social capital of the participants. The structural social capital is assessed based on responses on the following four domains (a) group membership, (b) support from groups, (c) support from individuals, and (d) collective action. The cognitive social capital is assessed based on following two domains; (a) trust and (b) social cohesion [56]. The SASCAT has been formally translated and validated into Bengali [57].

7. Family socio-economic status and food security

*Family income and expenditure*: Monthly family income and expenditure (as reported by the respondent) will be documented to measure the changes during the study period.

*Housing characteristics and household asset score*: A subset of the Bangladesh Demographic and Health Survey (BDHS) household questionnaire will be adopted to measure household wealth index of the participants. The BDHS is a nationally representative survey conducted every four years by the Government of Bangladesh in collaboration with the National Institute of Population Research and Training, Mitra and Associates and the United States Agency for International Development (USAID). This tool covers information on household ownership of a number of consumer items, ranging from a television to a bicycle, as well as housing characteristics, such as sources of drinking water, sanitation facilities, and type of material used for flooring, wall and roof [58].

*Household Food Insecurity Access Scale (HFIAS)*: The HFIAS tool (developed by the USAID) will be used to measure the household food insecurity level. HFIAS primarily focuses on “occurrence of food insecurity” followed by the frequency of the “occurrence of food insecurity” at the household level. The tool has nine questions and covers three domains of food insecurity (access); (a) anxiety and uncertainty about the household food supply, (b) Insufficient quality of food, and (c) insufficient food intake and its physical consequences [59]. Higher HFIAS score indicates higher food insecurity level.

*Food Consumption Score (FCS)*: The FCS tool (developed by the World Food Programme) will be used to assess dietary diversity at the household level. The tool has nine major food groups, a higher score indicates better quality of diet.

### Statistical methods

Data will be collected using paper-based forms. Research data will be anonymised and stored securely and separately for participant identifiable information. This will include monitoring secure data transfer from field to central office, data entry and quality control of completed forms, query about missing or invalid data and archiving of physical forms. Data will be collected using validated questionnaires. Data will be entered using Microsoft Access or SQL Server as the relational database engine. Any error identified during data entry or in data cleaning will be logged for field supervisor assessment and will be resolved after proper field verification. The physical data will be stored for seven years in a locked cabinet at head office of CSF Global based in Bangladesh as per national and international guidelines.

For statistical analysis, we will import the dataset into Stata 17. Primarily, we will perform an intention-to-treat analysis. Since our primary endpoint (i.e. HRQoL) will have continuous data, we will perform a mixed effect model (e.g. generalised linear mixed model) to calculate the effect of intervention according to the components of TAPQoL. We will also compare the TAPQoL scores between the three arms, and assess the differences using appropriate tests. Our proposed models will include the intervention arm as a fixed effect and study samples in each cluster as a random effect to explain cluster effects. To account for the intra-cluster correlation in calculating 95% CI and p-value, we will use a Sandwich estimate of standard error.

Baseline characteristics will be compared (as part of data matching) and adjusted using appropriate regression models. Descriptive statistics (frequencies, means and 95% confidence intervals) will be used to describe the sample at baseline and post-intervention. All analyses will be conducted using a significance level set at p<0.05. Data visualisation will be done using R Studio version 4.1.0.

### Trial monitoring

An independent Data Safety and Monitoring Board (DSMB) has been formed for this trial. The members of DSMB will meet monthly to monitor the safety of trial participants and the quality of trial data. The Chair of the DSMB will report to the Chief Investigator regarding issues related to data safety, quality of intervention and serious adverse event.

A serious adverse event will be defined as any event that results in injury, requires inpatient hospitalisation or prolongation of existing hospitalisation, or death or results in a persistent or significant disability or incapacity.

The DSMB will conduct a blinded interim analysis of effectiveness and safety endpoints once 210 participants have completed the trial. The DSMB may recommend continuing the trial, early termination of the trial, or modification of the trial. A recommendation to terminate the trial early will be made if there is clear evidence of a clinically harmful effect. The trial will not be stopped early on the grounds of futility.

### Consent and ethical approval

The trial has been approved by the Human Research Ethics Committee (HREC) of the Asian Institute of Disability and Development (Reference number: Southasia-hrec-2019-5-03) and the National Research Ethics Committee (NREC) of Bangladesh Medical Research Council (BMRC) (Reference Number: BMRC/NREC/2016-2019/251; Registration Number: 224 17 06 2019). The study has also been registered at the Australian New Zealand Clinical Trials Registry (ANZCTR) (reg number: ACTRN12619001750178). Informed written consent will be obtained from primary caregivers of children with CP before enrolling into the trial.

### Dissemination plan

The study findings will be shared with local and national Micro Finance Institutions and non-governmental organisations. We also aim to publish the trial findings in peer-reviewed journals and present at national and international conferences/workshops. Findings from this study, including key learnings, will be shared with stakeholders including rehabilitation practitioners working with children with CP in Bangladesh and other LMICs. The findings will also be shared with the participating parents of children with CP in the proposed study sites.

### Patient and public involvement

The integrated microfinance/livelihood and CBR program was conceptualised by the Chief Investigator (GK) during his engagement with parents of children with CP attending “Shishu Shorgo” (Children’s Heaven) Early Intervention and Rehabilitation Centre of Shahjadpur- a rural sub-district of Bangladesh. The community-based group-wise service delivery model was designed based on discussions with the parents of children with CP. Primary caregivers preferred a group-wise approach which is easy to access in terms of distance from home and frequency of session. It is expected that the mutual support from familiar group members will increase peer support and peer-led learning. Group dynamics towards a common goal will facilitate parents to practice the goal directed therapies effectively at home and achieve the targeted goals for individual children. Moreover, it is expected that these groups will continue to engage within themselves beyond the duration of the study.

The CBR program aims to train parents on evidence-based intervention and education related to CP (GDT and PTM) to ensure the sustainability of the rehabilitation practices and care of the participating children with CP. Following completion of the study, focused group discussions will be organised with each group of the intervention arms (Arm A and Arm B) to share the trial findings, explore primary caregiver’s experience of participation in the program, and identify scope for improvement of the integrated program for children with CP in rural Bangladesh.

## Results (pilot data)

We have piloted the activities of the proposed trial in the study area to determine the feasibility of the trial. The aim of the pilot study was to assess the acceptance rate and repayment rate of microfinance program among primary caregivers of children with CP. During the pilot phase, 20 ultra-poor families form the BCPR cohort (recruited between Jan 2015 and Nov 2018, n = 1,381) were identified who already have accessed microfinance from different local NGOs. The amounts of microfinance loan borrowed varied between ~100–300 AUD with 12-month loans payable in 45–46 weekly instalments. Of these families, the majority (n = 12) took loans to start/enhance income-generating activities (e.g. agricultural work, purchasing livestock, vehicle for hire, etc.). About 50% of borrowers repaid their first weekly instalment while the repayment schedule for the rest of the respondents was not due at the time of data collection.

## Supporting information

S1 Checklist SPIRIT checklist. (PDF)

S1 Table Schedule of study activities. (DOCX)

S1 Protocol Study protocol. (PDF)
