# Supplementary material for: Supporting Ultra Poor People with Rehabilitation and Therapy among families of children with Cerebral Palsy in rural Bangladesh (SUPPORT CP): Protocol of a randomised controlled trial
Source: PLoS One. 2021 Dec 31;16(12):e0261148. doi: 10.1371/journal.pone.0261148 (PMC8719685; doi:10.1371/journal.pone.0261148)
Supplement: S1 Checklist — (PDF) [file pone.0261148.s001.pdf]

STANDARD PROTOCOL ITEMS: RECOMMENDATIONS FOR INTERVENTIONAL TRIALS

SPIRIT 2013 Checklist: Recommended items to address in a clinical trial protocol and related documents*

| Section/item | Item No | Description |
|---|---|---|
| **Administrative information** | | |
| Title | 1 | Supporting Ultra Poor People with Rehabilitation and Therapy among families of children with Cerebral Palsy in rural Bangladesh (SUPPORT CP): protocol of a Randomised Controlled Trial |
| Trial registration | 2a | The study has also been registered at Australian New Zealand Clinical Trials Registry (ANZCTR) (reg number: ACTRN12619001750178) |
| | 2b | All items from the World Health Organization Trial Registration Data Set (Appendix-1) |
| Protocol version | 3 | Issue date: 15 Dec 2019; Version 1<br>Author: GK |
| Funding | 4 | This project is funded by the Research Foundation of Cerebral Palsy Alliance (Steptember Project Grant PG02218) and internal funding from CSF Global. |

| | | |
|---|---|---|
| Roles and responsibilities | 5a | Principal Investigator: Gulam Khandaker1,2,3,4,8 |

Associate Investigators:

Mahmudul Hassan Al Imam[1,2,3], Israt Jahan[1,2,3], Mohammad Muhit[1,2], Manik Chandra Das[1,2], Rosalie Power[2,4], Md Arifuzzaman Khan[1,2,5], Delwar Akbar[6], Nadia Badawi[4,7]

[1]CSF Global, Dhaka, Bangladesh
[2]Asian Institute of Disability and Development (AIDD), University of South Asia, Dhaka, Bangladesh
[3]School of Health, Medical and Applied Sciences, Central Queensland University, Rockhampton, Queensland, Australia
[4]Discipline of Child and Adolescent Health, Sydney Medical School, The University of Sydney, Sydney, Australia
[5]School of Public Health, The University of Queensland, Brisbane, Australia
[6]School of Business and Law, Central Queensland University, Rockhampton, Queensland, Australia
[7]Cerebral Palsy Alliance Research Institute, The University of Sydney, Sydney, Australia
[8]Central Queensland Public Health Unit, Central Queensland Hospital and Health Service, Rockhampton, Queensland, Australia

GK conceptualised the study. MM, AK, RP, DA, and NB provided specialist advice in designing the study. MH, AI, IJ, and MCD collected pilot data which helped to design the study. AK provided statistical expertise in clinical trial design. All authors provided substantial contributions to refinement of the study protocol and approved the final manuscript.

5b Trial Sponsor: The Research Foundation of Cerebral Palsy Alliance and CSF Global
Reference: Steptember Project Grant PG02218
Contact person: Peggy Harianto
Email: CPAResearchGrants@cerebralpalsy.org.au

5c Role of study sponsor and funders, if any, in study design; collection, management, analysis, and interpretation of data; writing of the report; and the decision to submit the report for publication, including whether they will have ultimate authority over any of these activities

This funding source had no role in the design of this study and will not have any role during its execution, analyses, interpretation of the data, or decision to submit results.

5d Composition, roles, and responsibilities of the coordinating centre, steering committee, endpoint adjudication committee, data management team, and other individuals or groups overseeing the trial, if applicable (see Item 21a for data monitoring committee)

Coordination Centre: The proposed trial will be coordinated by CSF Global.

Principal Investigator: (Gulam Khandaker)
-Design and conduct SUPPORT CP Trial
-Provide strategic directions
-Preparation of protocol and revisions
-Preparation of CRFs
-Organise steering committee meetings
-Publication of study reports

Steering Committee:
(The steering committee of the trial has been formed with all authors of the protocol)
-Agreement of final protocol
-Coordinate the activities of the project
-Development of GDT manual
-Development of Microfinance/livelihood program
-Study tools selection
-Facilitate training sessions for assessors
-Randomization and random assignment
-Data analysis and report writing
-Data Analysis
-Report Writing

Endpoint adjudication committee:
Safety and efficacy of therapeutic interventions of the trial will be assessed by a Research Physician of CSF Global. Being blinded to the given interventions, Research Physician will independently evaluate suspected adverse events reported by assessors.

Data Management Team: (Israt Jahan, Mahmudul Hassan Al Imam and Manik Chandra Das)
-Data acquisition
-Data entry and cleaning
-Data quality, integrity and safety

## Introduction

| Background and rationale | 6a | In LMICs, many families of children with CP live in extreme poverty, which contributes to poor health care access, delayed diagnosis, delayed intervention, overall poor health and wellbeing, and long-term reduced effectiveness of rehabilitation therapies. Our last 16 years of research in rural Bangladesh, which led to the development of Bangladesh CP register (BCPR - first ongoing population-based CP register in LMICs), confirms that in rural Bangladesh diagnosis of CP is delayed and there is limited or no access to evidence-based rehabilitation programs. Average age at diagnosis of CP in Bangladesh is 5y compared to 1.5y in high-income countries. We also found that even when rehabilitation programs were available access to care was negatively impacted by poverty. In Bangladesh, 97% of families of children with CP live below the poverty line. These families struggle to meet basic needs and their child's rehabilitation often does not feature high on the agenda. Therefore, an integrated approach combining the physical rehabilitation of children with CP and the economic empowerment of their family is required for tangible long-term improvements.<br><br>Microfinance/livelihood support is an effective tool for improving economic, human (including non-cognitive skills), and social capital of disadvantaged people in LMICs particularly vulnerable groups such as women and children. Microfinance/livelihood support programs can improve health by increasing financial access and service utilisation. Combining microfinance with health interventions has yielded promising results in the fields of HIV, malaria, and breast feeding in Africa.<br><br>In addition, non-experimental and quasi-experimental studies testing the effectiveness of integrated health and economic interventions report significant improvements in reproductive and child health, nutrition, and immunisation. Non-cognitive skills are considered as important predictors of socio-economic outcomes, including` the development of small-scale businesses in African context. Moreover, interaction between groups in a society reduces prejudice and promotes inter-group cooperation.<br><br>To be effective, interventions need to be tailored according to the needs of the target population. Influential work by Professor Sir Michael Marmot, Chair of the World Health Organization (WHO) Commission on Social Determinants of Health, and others have demonstrated that socioeconomic factors are important determinants of health. Even in a developed country like UK, the average life expectancy in poorer areas of Glasgow is about 20 years shorter than that for rest of the country. This gap can be explained as a direct result of poverty and related social disadvantage. Tangible improvements in overall health status of people living in poverty can only be achieved by focusing on improving both health and economic/social capital. However, to our knowledge no studies have examined the effectiveness of an integrated health and economic approach for children with CP and their families in LMICs. |
|---|---|---|

6b Explanation for choice of comparators:

The outcomes of Integrated microfinance/livelihood and community-based rehabilitation (IMCBR) arm will be assessed against the outcomes of community-based rehabilitation (CBR) only and care as usual arms. Comparison with the CBR only arm will allow us to determine what extent of outcome directly attributed by microfinance/livelihood program and comparison against care as usual group will help us to evaluate effectiveness of integrated IMBCR program in achieving study objectives.

Objectives 7 Specific objectives or hypotheses:

Aims

The aim of this study is to test the effectiveness of an "Integrated Microfinance/livelihood and CBR program" (IMCBR) targeted to children with CP and their parents from ultra-poor families in rural Bangladesh. The program aims to improve the HRQoL, motor function, communication and nutritional status of children with CP; mental health, HRQoL and social capital of their parents; and socio-economic status and food security of their families.

Our specific objectives are;

1. To conduct an RCT with three parallel arms comparing (a) IMCBR, (b) CBR alone, and (c) care-as-usual (i.e. no intervention).
2. To measure the effectiveness of IMCBR in improving the HRQoL, motor function, communication, and nutritional status of children with CP from ultra-poor families living in rural Bangladesh.
3. To measure the effectiveness of IMCBR in improving mental health, HRQoL, and social capital of parents of children with CP living in rural Bangladesh.
4. To measure the effectiveness of IMCBR in improving the socio-economic status of ultra-poor families of children with CP living in rural Bangladesh.

**Hypothesis**

We hypothesise that compared to care-as-usual and CBR alone, the IMCBR program will be more effective in improving HRQoL, motor function, communication, and nutritional outcomes of children with CP from ultra-poor families; and the mental health, HRQoL, and social capital of their primary caregivers; and overall improvement in socio-economic status of the ultra-poor families of children with CP in rural Bangladesh.

| | | |
|---|---|---|
| Trial design | 8 | Description of trial design including type of trial (eg, parallel group, crossover, factorial, single group), allocation ratio, and framework (eg, superiority, equivalence, noninferiority, exploratory)<br><br>**Overview of design:**<br>This will be a cluster randomised controlled trial comprising three arms. The unit of randomisation will be a cluster. Clusters randomised to intervention arms of the trial (i.e. IMCBR and CBR arms) will receive interventions following the protocol outlined in later sections. The interventions will be provided to dyads consisting of child with CP and their primary caregivers. Care-as-usual arm of the trial will not receive any active intervention. |

**Methods: Participants, interventions, and outcomes**

| | | |
|---|---|---|
| Study setting | 9 | Description of study settings (eg, community clinic, academic hospital) and list of countries where data will be collected. Reference to where list of study sites can be obtained<br><br>The study will be implemented in Shahjadpur sub-district (~324.15 sq. km) of Sirajganj district located in the northern part of Bangladesh. The study site is comprised of ~70,998 households with a total population of ~561,076 (child population aged 0-18 years ~226,114), and 12,117 live births per annum. The study site constitutes a complex socio-demographic locale including urban, rural, and hard-to-reach areas and represents the overall socio-demographic and economic characteristics of rural areas in Bangladesh. |

| | | |
|---|---|---|
| Eligibility criteria | 10 | Inclusion and exclusion criteria for participants. If applicable, eligibility criteria for study centres and individuals who will perform the interventions (eg, surgeons, psychotherapists) |

**Inclusion/Exclusion criteria**

Participants will be considered eligible for participation based on the following criteria:

1. Children with CP aged ≤5 years, classified as from an ultra-poor family (i.e. per day per capita income <1.90 USD) and registered in the BCPR. BCPR classifies children with CP following the case definition adopted from the Surveillance of CP in Europe (SCPE) and the Australian CP Register (ACPR).
2. Primary caregiver (e.g. parent, sibling, grandparent of the child with CP)
3. Primary caregiver has capacity to give informed consent and is willing to take part in the study including microfinance/livelihood arm along with their child with CP.

Participants will be considered ineligible for participation based on the following criteria:

1. Currently in receipt of microfinance/livelihood support from another source.
2. Currently participating in any other clinical trial or intervention program.

| | | |
|---|---|---|
| Interventions | 11a | **Arm A- Integrated Microfinance/livelihood and Community-Based Rehabilitation (IMCBR)** |

Participants randomised to IMCBR will be supported to create microfinance/livelihood groups (10 participant-pairs per group). The groups will be formed voluntarily along geographical boundaries to facilitate participation, retention, and meeting logistics. Each cluster will meet weekly to discuss microfinance/livelihood activities (e.g. weekly credit collection and troubleshooting) (90 minutes) and for CBR with children with CP comprising early intervention and primary caregiver's education (90 minutes).

*A.1 Microfinance/livelihood program details*

Group meetings will be organised with members of each cluster to discuss (i) details of the program, (ii) potential benefits and challenges of participation in the program, and (iii) motivations for participation. Participants of each cluster can then apply for a loan/livelihood support; a minimum 10% deposit of the requested loan/livelihood support amount in the form of savings is required and is admissible immediately after cluster formation. Once the application is completed, loan approval and disbursement of the loan will occur approximately within one week.

Amount, return cycle of loan and investment areas: Each of the ultra-poor families will receive a loan/livelihood support amounting/equivalent to ~100-300AUD at 12% flat interest rate. The return cycle will be one year with weekly repayment schedule. Common investment areas for the ultra-poor loan will be for goat or cattle rearing, seeds for agriculture, home-based weaving, and handicraft business.

*A.2 CBR*

There will be two major components of the CBR program, which will occur during cluster meetings following the microfinance/livelihood portion.

a. Goal Directed Training (GDT): Community-based GDT focused on motor learning will be conducted with children with CP and their primary caregivers. GDT is an activity-based approach to therapy where meaningful, client-selected (i.e. caregivers of children with CP) goals are used to provide opportunities for problem solving and to indirectly drive the movements required to successfully meet task demands. Evidence from a meta-analysis shows that GDT based interventions are highly effective and should be the gold standard treatment for CP. In this study, GDT will be delivered by child's primary caregiver (participating parent).

*b. Parents Training Module (PTM):* Primary caregivers will participate in PTM to learn basic therapeutically correct skills for the day-to-day care and support of their child with CP embedded in the principles of GDT. This study will follow the PTM 'Getting to know cerebral palsy' which includes 10 modules and covers topics; introduction to CP, evaluating your child, positioning and carrying, communication, everyday activities, feeding your child, play, disability in your local community, running your own parent support group, and assistive devices and resources.

Specially trained Community Rehabilitation Officers (CRO's) will facilitate each of the cluster meetings (both microfinance/livelihood and CBR activities). The CROs will facilitate microfinance/livelihood discussions and lead the GDT and PTM sessions with the aim to upskill primary caregivers so that they can continue to deliver GDT independently at home. Prior to implementation of the RCT, CROs will take part in a 5-day training by Research Physiotherapist. The CRO training will cover the following areas; (i) socio-cultural considerations in working with primary caregivers of children with CP, (ii) introduction to microfinance/livelihood support program management, (iii) developmental milestones and development in children with CP, (iv) therapeutic principles, (v) goal directed training, (vi) activity focused therapies, (vii) basic speech development strategies, (viii) contraindications of therapies, (ix) research ethics etc..

**Arm B- CBR alone**

Participants from clusters randomised to this arm will attend a weekly rehabilitation session at a local focal point (preferably one of the group members home). Each session will last for 90 minutes and will be identical to the CBR component of IMCBR (discussed above); however, the microfinance/livelihood component will not be provided

**Arm C- Care-as-usual (i.e. no intervention)**

This group will not receive any active intervention. Once children with CP are identified and randomised into clusters, the 'care-as-usual' participants will be provided with basic education on early intervention and rehabilitation and will be encouraged to access healthcare via usual routes, which typically include treatment in government hospitals.

| | | |
|---|---|---|
| | 11b | Criteria for discontinuing or modifying allocated interventions for a given trial participant (eg, drug dose change in response to harms, participant request, or improving/worsening disease)<br><br>CBR interventions, in both IMCBR and CBR only arms, can only be discontinued when a child is sick and/ or suffering from serious adverse events. |
| | 11c | Strategies to improve adherence to intervention protocols, and any procedures for monitoring adherence (eg, drug tablet return, laboratory tests)<br><br>Microfinance/livelihood activities (i.e. loan) itself would be a great motivating factor for the participants of the IMCBR arm to feel motivated to intervention. Additionally, we would conduct weekly phone follow up and monthly home visits to ensure that participants are adhered to intervention. |
| | 11d | Relevant concomitant care and interventions that are permitted or prohibited during the trial<br><br>Children with CP from all three study arms will continue accessing need-based medical and therapy support from other sources as per their family's preferences. Frequency and duration of access to local medical/therapy services will be recorded during follow-up assessments and included in analysis. |
| Outcomes | 12 | Primary, secondary, and other outcomes, including the specific measurement variable (eg, systolic blood pressure), analysis metric (eg, change from baseline, final value, time to event), method of aggregation (eg, median, proportion), and time point for each outcome. Explanation of the clinical relevance of chosen efficacy and harm outcomes is strongly recommended<br><br>**Outcome measures**<br>Following outcomes will be measured at baseline, at 6 months, 12 months, and 18 months.<br><br>***Primary outcomes***<br>1. Health-related quality of life (HRQoL) of children with CP<br><br>***Secondary and exploratory outcomes***<br>1. Motor function of children with CP<br>2. Communication function of children with CP<br>3. Nutritional status of children with CP<br>4. Mental health of primary caregivers of children with CP<br>5. Health-related quality of life of primary caregivers of children with CP<br>6. Social capital of primary caregivers of children with CP<br>7. Family socio-economic status and food security |

| | | |
|---|---|---|
| Participant timeline | 13 | Time schedule of enrolment, interventions (including any run-ins and washouts), assessments, and visits for participants. A schematic diagram is highly recommended (see Appendix-2) |
| Sample size | 14 | Estimated number of participants needed to achieve study objectives and how it was determined, including clinical and statistical assumptions supporting any sample size calculations<br><br>Sample size for this cluster RCT has been computed based on methods described in Donner et al. We will recruit seven clusters in each arm, and each cluster will consist of 10 CP children with CP- primary caregivers dyads totalling 21 clusters of 210 dyads. Based on our pilot data and existing literature we predict 35% improvement of health-related quality of life (HRQoL) in the IMCBR group, 20% improvement in the CBR alone group, and 5% improvement in the care-as-usual group. With a sample size of 210 dyads, this study will have 80% statistical power to detect these effects (two sided α-value=0.05). Power calculation takes into account up to 20% sample attrition by the end of trial. A homogeneous study population will allow us to balance randomisation considering intra-cluster correlation of 0.5 and coefficient of variation in cluster size of 0.5. |
| Recruitment | 15 | Strategies for achieving adequate participant enrolment to reach target sample size<br>Considering the number of registrants from Shahjadpur (i.e.~1125), there are ~373 children eligible to participate in the study. Therefore, recruitment of 210 children and their primary caregiver in the trial (<60% of the available pool) is feasible. Sociodemographic, economic, and health data of these children and their families are already recorded in the BCPR allowing quick identification and recruitment. All families enrolled in the BCPR have also been mapped using Geographic Information System (GIS). |

**Methods: Assignment of interventions (for controlled trials)**

Allocation:

| | | |
|---|---|---|
| Sequence generation | 16a | Method of generating the allocation sequence (eg, computer-generated random numbers), and list of any factors for stratification. To reduce predictability of a random sequence, details of any planned restriction (eg, blocking) should be provided in a separate document that is unavailable to those who enrol participants or assign interventions<br><br>The study will include 21 clusters randomised to three arms (7 clusters each) and allocated by 1:1:1 ratio. Each cluster will come from a 'mouza', the smallest local administrative unit in Bangladesh, and will comprise 10 CP child-primary caregiver dyads. In order to minimize 'contamination' of intervention, clusters will be separated from each other by buffer areas comprising villages not taking part in the study. We will adjust cluster margins so that they are aligned with natural divisions that separate residents in community (e.g., rivers). The randomization process will be executed by an independent statistician. |
| Allocation concealment mechanism | 16b | Mechanism of implementing the allocation sequence (eg, central telephone; sequentially numbered, opaque, sealed envelopes), describing any steps to conceal the sequence until interventions are assigned<br><br>Participants will be randomised using by an independent statistician. Allocation concealment will be ensured, as statistician will not release the randomisation code until the participant has been recruited into the trial, which takes place after all baseline measurements have been completed. |
| Implementation | 16c | Who will generate the allocation sequence, who will enrol participants, and who will assign participants to interventions<br><br>The generation of allocation sequence, participant enrolment, and intervention assignment will be done by an independent statistician. |
| Blinding (masking) | 17a | Who will be blinded after assignment to interventions (eg, trial participants, care providers, outcome assessors, data analysts), and how<br><br>The interventions will be open to participants and investigators, however the outcome assessments will be observer blinded. An independent team, informed on the purpose and importance of blinding for this study, will conduct outcome assessments. The assessors will be masked/blinded about the interventions and the outcome assessment questionnaire will be designed in a manner that does not disclose the intervention. |

| | |
|---|---|
| 17b | If blinded, circumstances under which unblinding is permissible, and procedure for revealing a participant’s allocated intervention during the trial<br><br>In no circumstance, the allocation of intervention will be unblinded to assessor. |

**Methods: Data collection, management, and analysis**

| | | |
|---|---|---|
| Data collection methods | 18a | Plans for assessment and collection of outcome, baseline, and other trial data, including any related processes to promote data quality (eg, duplicate measurements, training of assessors) and a description of study instruments (eg, questionnaires, laboratory tests) along with their reliability and validity, if known. Reference to where data collection forms can be found, if not in the protocol |

**Outcome measures**
Following outcome related data will be collected at baseline, at 6 months, 12 months, and 18 months.

***Primary outcomes***
1. Health-related quality of life (HRQoL) of children with CP
*TNO-AZL Preschool children Quality of Life (TAPQOL):* The TAPQOL is a validated tool designed to measure the parent perceived (i.e. proxy-reported) HRQoL of preschool children. The multidimensional instrument covers four domains; (a) Physical functioning, (b) Social functioning, (c) Cognitive functioning, and (d) Emotional functioning. The tool contains 43 items covering 12 scales (the number of items per scale ranges from 3 to 7), with higher scores indicating better HRQoL.

***Secondary and exploratory outcomes***
1. Motor function of children with CP
*Gross Motor Function Measure (GMFM)-66:* The GMFM-66 is designed to assess the gross motor function in children with CP and measure changes overtime. The 66 activities in GMFM-66 cover five dimensions; (a) lying and rolling, (b) sitting, (c) crawling and sitting, (d) standing, and (e) walking, running and jumping. The total score ranges between 0-100.

*Gross Motor Function Classification System (GMFCS):* The GMFCS is a five-level classification of functional motor abilities of children with CP. The Level I indicates minimal functional deficits among children with CP, whereas the level V indicates the highest level of functional deficits among children with CP and children categorised as GMFCS level V are usually fully dependent.

*Classification of CP based on motor function and topographical distribution:* The predominant motor type i.e. spastic CP and non-spastic CP (dyskinesia, ataxia and hypotonia) and topographical distribution i.e. monoplegia, hemiplegia, diplegia, triplegia, and quadriplegia will be assessed.

2. Communication function of children with CP
*Communication Function Classification System (CFCS):* The CFCS is a validated tool to categorise children's communication skills into five mutually exclusive levels of everyday communicative function. Classifications are made according to the descriptions of the levels and of the distinctions between them.

3. Nutritional status of children with CP
Anthropometric measurements will be taken using standard guidelines of WHO and will be analysed to assess nutritional status of children. Following measurements will be collected at each assessment; (a) weight in kilograms, (b) height in centimetre (cm), (c) mid-upper arm circumference in cm, (d) skin-fold thickness in millimetre, and (e) head-circumference in cm.

4. Mental health of primary caregivers of children with CP
*Depression, Anxiety, Stress Scale – Short Form -21 (DASS-21):* The DASS-21 is the shortened version of the DASS to assess symptoms of depression, anxiety and stress among adults. There are 21 items in this scale with four response options: 0 "Did not apply to me at all–Never", 1 "Applied to me to some degree, or some of the time–Sometimes", 2 "Applied to me to a considerable degree, or a good part of time–Often" to 3 "Applied to me very much, or most of the time–Almost always".

5. Health-related quality of life of primary caregivers of children with CP
*Short Form 12-Version 2 (SF 12v2):* The SF-12 is a validated tool to measure self-reported HRQoL of adults [47]. The tool contains 12 questions covering eight domains; (a) physical functioning, (b) role physical, (c) bodily pain, (d) general health, (e) vitality, (f) social functioning, (g) role emotional, and (h) mental health.

6. Social capital of primary caregivers of children with CP
*Short version of Adapted Social Capital Assessment Tool (SASCAT):* The tool measures structural and cognitive social capital of the participants. The structural social capital is assessed based on responses on following four domains (a) group membership, (b) support from groups, (c) support from individuals, and (d) collective action.

7. Family socio-economic status and food security
*Family income and expenditure:* Monthly family income and expenditure (as reported by the respondent) will be documented to measure any changes during the study period.

*Housing characteristics and household asset score:* A subset of the Bangladesh Demographic and Health Survey (BDHS) household questionnaire will be adopted to measure household wealth index of the participants.

*Household Food Insecurity Access Scale (HFIAS):* The HFIAS tool (developed by USAID) will be used to measure the household food insecurity level. HFIAS primarily focuses on occurrence of food insecurity at household level followed by frequency of the occurrence of food insecurity. The tool has nine questions and covers three domains of food insecurity (access); (a) anxiety and uncertainty about the household food supply, (b) Insufficient quality of food, and (c) insufficient food intake and its physical consequences [52]. Higher HFIAS score indicates higher food insecurity level.

*Food Consumption Score (FCS):* The FCS tool (developed by World Food Programme) will be used to assess dietary diversity at household level. The tool has nine major food groups, higher score indicates better quality of diet.

The assessors will be trained thoroughly on the CRF by one of the co-investigators. Then the assessors will be asked to assess 5 children with CP and their parents. The collected forms will be reviewed by the trainer and a feedback session will be arranged to share if there is any issue with data collection and how that can be improved.

18b Plans to promote participant retention and complete follow-up, including list of any outcome data to be collected for participants who discontinue or deviate from intervention protocols

Microfinance/livelihood programs (i.e. loan) itself would be a great motivating factor for the parents of the IMCBR arm to adhere to intervention. In addition to that we would conduct weekly phone follow up and monthly home visits to ensure participant retention. In case of a discontinued respondent, primary outcome data i.e. HRQoL of children with CP will be collected.

Data management

19 Plans for data entry, coding, security, and storage, including any related processes to promote data quality (eg, double data entry; range checks for data values). Reference to where details of data management procedures can be found, if not in the protocol

Data will be collected using paper-based forms. Research data will be anonymised and stored securely and separately for participant identifiable information. This will include monitoring secure data transfer from field to central office, data entry and quality control of completed forms, querying of missing or invalid data, and archiving of physical forms. Data will be collected using validated questionnaires. Data will be entered into PCs using Microsoft Access or SQL Server as the relational database engine. Any error identified during data entry or in data cleaning will be logged for field supervisor assessment and will be resolved after proper field verification. The physical data will be stored for 7 years in a locked cabinet at head office of CSF Global based in Bangladesh as per national and international guidelines.

| | | |
|---|---|---|
| Statistical methods | 20a | Statistical methods for analysing primary and secondary outcomes. Reference to where other details of the statistical analysis plan can be found, if not in the protocol<br><br>Intention-to-treat analysis will compare improvements in primary and other outcomes between groups controlling for baseline measures. Descriptive statistics (frequencies, means and 95% confidence intervals) will be used to describe the sample at baseline and post-intervention. Hypothesis testing will be done using appropriate statistical procedures based on type of data. For example, Chi-square test will be used to measure statistical difference between proportions of outcome among study arms. All analyses will be conducted using STATA 15, with the significance level set at $p<0.05$. Data visualisation will be done using R studio/GraphPad Prism 7. |
| | 20b | Methods for any additional analyses (eg, subgroup and adjusted analyses)<br>To account for the intra-cluster correlation in calculating 95% CI and p-value, we will use Sandwich estimate of standard error. Baseline characteristics will be compared and adjusted using appropriate regression models. |
| | 20c | Definition of analysis population relating to protocol non-adherence (eg, as randomised analysis), and any statistical methods to handle missing data (eg, multiple imputation)<br><br>Validity of results will be ascertained using baseline data, including systematic differences between those completing the intervention and drop-outs. Sensitivity analyses will also be done using multiple imputed data sets to investigate the effect of possibly differential drop-out, if required. |
| **Methods: Monitoring** | | |
| Data monitoring | 21a | Composition of data monitoring committee (DMC); summary of its role and reporting structure; statement of whether it is independent from the sponsor and competing interests; and reference to where further details about its charter can be found, if not in the protocol. Alternatively, an explanation of why a DMC is not needed<br><br>An independent Data Safety and Monitoring Board (DSMB) has been formed for this trial. The members of DSMB will meet monthly to monitor the safety of trial participants and the quality of trial data. The Chair of the DSMB will report to Chief Investigator regarding issues related to data safety, quality of intervention and serious adverse event. |

| | | |
|---|---|---|
| | 21b | Description of any interim analyses and stopping guidelines, including who will have access to these interim results and make the final decision to terminate the trial<br><br>The DSMB will conduct a blinded interim analysis of effectiveness and safety end points once 210 participants have completed the trial. The DSMB may recommend continuing the trial, early termination of the trial, or modification of the trial. A recommendation to terminate the trial early will be made if there is clear evidence of a clinically harmful effect. The trial will not be stopped early on the grounds of futility. |
| Harms | 22 | Plans for collecting, assessing, reporting, and managing solicited and spontaneously reported adverse events and other unintended effects of trial interventions or trial conduct<br><br>A serious adverse event will be defined as any event that results in injury, requires inpatient hospitalisation or prolongation of existing hospitalisation, or death or results in a persistent or significant disability or incapacity. Serious adverse events will be monitored by Research Physician (RP)-a representative of Data Safety and Monitoring Board. RP will review compliance and quality of interventions and monitor any serious adverse events on a 6 months basis and report to the Chief Investigator advising whether the adverse events are related to intervention provided in the study. |
| Auditing | 23 | Frequency and procedures for auditing trial conduct, if any, and whether the process will be independent from investigators and the sponsor<br><br>A periodic independent review of activities and processes of the trial and collected data will be conducted and required corrections will be implemented to preserve the integrity of the trial. The audit will include participant enrolment, consent, eligibility, and allocation to study groups; adherence to trial interventions and policies to protect participants, including reporting of harms and completeness, accuracy, and timeliness of data collection. |

**Ethics and dissemination**

| | | |
|---|---|---|
| Research ethics approval | 24 | Plans for seeking research ethics committee/institutional review board (REC/IRB) approval<br><br>The trail has been approved by National Research Ethics Committee (NREC) of Bangladesh Medical Research Council (BMRC) (Reference Number: BMRC/NREC/2016-2019/251; Registration Number: 224 17 06 2019). The study has also been registered at Australian New Zealand Clinical Trials Registry (ANZCTR) (reg number: ACTRN12619001750178). |

| | | |
|---|---|---|
| Protocol amendments | 25 | Plans for communicating important protocol modifications (eg, changes to eligibility criteria, outcomes, analyses) to relevant parties (eg, investigators, REC/IRBs, trial participants, trial registries, journals, regulators)<br><br>Any modifications to the protocol which may impact on the conduct of the study, potential benefit of the patient or may affect patient safety, including changes of study objectives, study design, patient population, sample sizes, study procedures, or significant administrative aspects will require a formal amendment to the protocol. Such amendment will be agreed upon by all investigators and approved by Bangladesh Medical Research Council (BMRC) and notified to trial register and protocol publisher prior to implementation. |
| Consent or assent | 26a | Who will obtain informed consent or assent from potential trial participants or authorised surrogates, and how (see Item 32)<br><br>Trained Research Physiotherapist will orient primary caregivers of children with CP regarding the trial. Patients will also receive a Participant Information Sheet (PIS). Research Physiotherapist will discuss the trial with parents considering the information provided in PIS. Parents will then be able to have an informed discussion with the Research Physiotherapist. Research Physiotherapist will obtain written consent from primary caregivers willing to participate in the trial. Information sheets and consent forms will be provided to all parents involved in the trial. The PIS and Consent Form have been translated into Bengali. |
| | 26b | Additional consent provisions for collection and use of participant data and biological specimens in ancillary studies, if applicable<br><br>Not applicable. |
| Confidentiality | 27 | How personal information about potential and enrolled participants will be collected, shared, and maintained in order to protect confidentiality before, during, and after the trial<br><br>The coded non-identifiable data will be stored on a secure server maintained by CSF Global. The dataset of this study will be on a fire-wall protected server and will be accessible by the administrator only, with computers protected by secure password log-on instigated after five minutes of computer inactivity. An appropriate data backup schedule will be in place. No data will be stored on any researcher's local computer environment. No identifiable information will be presented in any reports nor made apparent through detail of specific personal or health characteristics. |

| | | |
|---|---|---|
| Declaration of interests | 28 | Financial and other competing interests for principal investigators for the overall trial and each study site<br><br>The authors declare that there is no competing interest. |
| Access to data | 29 | Statement of who will have access to the final trial dataset, and disclosure of contractual agreements that limit such access for investigators<br><br>Chief Investigator will have access to the cleaned data sets. The dataset of this study will be kept on a fire-wall protected server and will be accessible by the administrator only, with computers protected by secure password log-on instigated after five minutes of computer inactivity. To ensure confidentiality, data dispersed to project team members will be blinded of any identifying participant information. |
| Ancillary and post-trial care | 30 | Provisions, if any, for ancillary and post-trial care, and for compensation to those who suffer harm from trial participation<br><br>After completion of the trial children with CP that are enrolled into the study will be able to access further rehabilitation care at “Shishu Shorgo” (Children’s heaven) Early Intervention and Rehabilitation Centres of CSF Global. Costs (hospital admission, laboratory tests, consultancy fees, transport and logistics) associated with any serious adverse events arising from interventions given in the trial will be borne by CSF Global. |
| Dissemination policy | 31a | Plans for investigators and sponsor to communicate trial results to participants, healthcare professionals, the public, and other relevant groups (eg, via publication, reporting in results databases, or other data sharing arrangements), including any publication restrictions<br><br>The study findings will be shared with local and national Micro Finance Institutions and non-governmental organisations. We also aim to publish the trial findings in peer reviewed journals and presented at national and international conferences/workshops. Findings from this study, including key learnings, will be shared with stakeholders including rehabilitation practitioners working with children with CP in Bangladesh and other LMICs. The findings will also be shared with the participating parents of children with CP in the proposed study sites. |
| | 31b | Authorship eligibility guidelines and any intended use of professional writers<br><br>Authorship will be given to associate investigators following the International Committee of Medical Journal Editors’ guidelines. |

| | | |
|---|---|---|
| | 31c | Plans, if any, for granting public access to the full protocol, participant-level dataset, and statistical code<br><br>Not applicable. |
| **Appendices** | | |
| Informed consent materials | 32 | Model consent form and other related documentation given to participants and authorised surrogates<br><br>The Participant Information Sheet (Appendix-3) and Consent Form (Appendix-4) have been attached. |
| Biological specimens | 33 | Plans for collection, laboratory evaluation, and storage of biological specimens for genetic or molecular analysis in the current trial and for future use in ancillary studies, if applicable<br><br>Not applicable. |

*It is strongly recommended that this checklist be read in conjunction with the SPIRIT 2013 Explanation & Elaboration for important clarification on the items. Amendments to the protocol should be tracked and dated. The SPIRIT checklist is copyrighted by the SPIRIT Group under the Creative Commons "Attribution-NonCommercial-NoDerivs 3.0 Unported" license.

**Appendix-1: Items from the World Health Organization Trial Registration Data Set**

| Data Category | Information |
|---|---|
| Primary Registry and Trial identifying number | Australian New Zealand Clinical Trials Registry ACTRN12619001750178 |
| Date of registry | 10 Dec 2019 |
| Secondary identifying number | NA |
| Source of monetary support | Research Foundation of Cerebral Palsy Alliance (Steptember Project Grant PG02218) and internal funding from CSF Global. |
| Primary sponsor | Research Foundation of Cerebral Palsy Alliance |
| Secondary sponsor | CSF Global |
| Contact for public query | Gulam Khandaker (Email: gulam.khandaker@health.nsw.gov.au) |
| Contact for scientific query | Gulam Khandaker (Email: gulam.khandaker@health.nsw.gov.au) |
| Public title | Supporting Ultra Poor People with Rehabilitation and Therapy among families of children with Cerebral Palsy in rural Bangladesh (SUPPORT CP): protocol of a Randomised Controlled Trial |
| Scientific title | Supporting Ultra Poor People with Rehabilitation and Therapy among families of children with Cerebral Palsy in rural Bangladesh (SUPPORT CP): protocol of a Randomised Controlled Trial |
| Country of recruitment | Bangladesh |
| Health conditions or problems studied | Cerebral palsy (CP) |
| Interventions | Arm-A: Integrated microfinance/livelihood and community-based rehabilitation program (IMCBR), Arm-B: community-based rehabilitation, Arm-C: Care-as-usual (i.e. no intervention) |
| Key inclusion and exclusion criteria | Inclusion criteria:<br>1. Children with CP aged ≤5 years, classified as ‘from an ultra-poor family’<br>2. Primary caregiver (e.g. parent, sibling, grandparent of the child with CP) |

| | |
|---|---|
| | Exclusion criteria:<br>1. Current recipient of any microfinance/livelihood support from another source.<br>2. Currently participating in any other clinical trial or intervention program. |
| Study type | Interventional<br>Allocation: cluster randomised controlled intervention model. Parallel assignment. Assessor blinded.<br>Primary purpose: to measure the effect of IMCBR on Health-Related Quality of Life (HRQoL) of children with CP. |
| Date of first enrolment | Dec 2019 |
| Target sample size | 210 |
| Recruitment status | Completed |
| Primary outcome(s) | Improvement in HRQoL of children with cerebral palsy. |
| Key secondary outcomes | Improvement in motor function, communication and nutritional status of children with CP; mental health, HRQoL and social capital of their parents; and socio-economic status and food security of their families. |

## Appendix-2: Timeline and Procedure of SUPPORT CP Trial

| | | | | -1 | 0 | T | F1 | F2 | F3 |
|---|---|---|---|---|---|---|---|---|---|
| **Sr No** | **Activity/Assessment** | **Case Report Form (Y/N)** | **Staff Member** | **Screening/ Consent** | **Baseline Assessment/ Randomization** | **Trial Commencement** | **6m Follow-Up** | **12m Follow-Up** | **18m Follow-Up** |
| 1 | Group formation for Arm-A | N | Trial Coordinator | X | | | | | |
| 2 | Pre-Screening Consent | N | Trial Coordinator | X | | | | | |
| 3 | Screening Log | N | Trial Coordinator | X | | | | | |
| 4 | Questionnaire / Consent Form | N | Trial Coordinator | X | | | | | |
| 5 | Demographic questionnaire | Y | Assessor | | X | | X | X | X |
| 6 | Health-related quality of life (HRQoL) of children with CP: using TAPQOL | Y | Assessor | | X | | X | X | X |
| 7 | Gross Motor Function Measure (GMFM)-66 | Y | Assessor | | X | | X | X | X |
| 8 | Gross Motor Function Classification System (GMFCS) | Y | Assessor | | X | | X | X | X |
| 9 | Motor Types: Motor type | Y | Assessor | | X | | X | X | X |
| 10 | Communication Function Classification System (CFCS) | Y | Assessor | | X | | X | X | X |
| 11 | Nutritional status of the children with CP | Y | Assessor | | X | | X | X | X |
| 12 | Depression, Anxiety, Stress Scale – Short Form-21 (DASS-21) | Y | Assessor | | X | | X | X | X |
| 13 | Short Form 12-Version 2 (SF 12v2) | Y | Assessor | | X | | X | X | X |

| | | | | | | | | | |
|---|---|---|---|---|---|---|---|---|---|
| 14 | Short version of Adapted Social Capital Assessment Tool (SASCAT) | Y | Assessor | | X | | X | X | X |
| 15 | Family income | Y | Assessor | | X | | X | X | X |
| 16 | Household asset score | Y | Assessor | | X | | X | X | X |
| 17 | Household Food Insecurity Access Scale (HFIAS) | Y | Assessor | | X | | X | X | X |
| 18 | Randomization | Y | Trial Coordinator | | X | | | | |
| 19 | Commencement of Microfinance and CBR activities | Y | Assessor | | | X | | | |
| 20 | Termination Form | Y | Trial Coordinator | | | | | | X |
| 21 | Serious Adverse Event Form | Y | Trial Coordinator | | As needed throughout the protocol | | | | |
| 22 | Progress Notes | N | Trial Coordinator | | X | X | X | X | X |
| 23 | Communication Log | N | Trial Coordinator | | Every group meeting or related communication | | | | |

# গবেষণা সম্পর্কিত তথ্যাবলী

গবেষণার নামঃ

বাংলাদেশের গ্রামে বসবাসরত সেরেব্রাল পালসিতে আক্রান্ত শিশু ও তাদের অতি দরিদ্র পরিবারের উপর সমাজ-ভিত্তিক পূনর্বাসন এবং আয়বৃদ্ধিমূলক কর্মসূচির প্রভাব নিরীক্ষা (সাপোর্ট সিপি ট্রায়াল)

গবেষণার লক্ষ্য ও উদ্দেশ্যঃ

বাংলাদেশের প্রত্যন্ত গ্রামাঞ্চলে সেরেব্রাল পালসিতে আক্রান্ত শিশুর রোগ নির্ণয়, দ্রুত চিকিৎসা গ্রহনে এবং পূনর্বাসন প্রক্রিয়ার পথে প্রধান অন্তরায় হচ্ছে দারিদ্র। যেসকল পরিবারে সেরেব্রাল পালসিতে আক্রান্ত শিশু আছে সেরকম পরিবারের শতকরা ৯৭ ভাগ দারিদ্র সীমার নীচে বাস করে। তাই নিম্ন ও মধ্যম আয়ের দেশগুলোতে সেরেব্রাল পালসি আক্রান্ত শিশুর উন্নতির (এই উন্নতির মাঝে জীবনযাত্রার মানোন্নয়ন, শারীরিক সক্ষমতার মান বাড়ানো ও শরীরে পুষ্টিমান রয়েছে) জন্যে চিকিৎসার পাশাপাশি পরিবারের অর্থনৈতিক ও সামাজিক উন্নয়নের জন্যেও ব্যবস্থা গ্রহন করা প্রয়োজন। এই গবেষণায় আমরা বাংলাদেশের সেরেব্রাল পালসিতে আক্রান্ত শিশু আছে এমন অতি দরিদ্র পরিবারে আয় বৃদ্ধিমূলক ও সমাজভিত্তিক পূনর্বাসন কর্মসূচীর প্রভাব মূল্যায়ন ও নিরীক্ষা করবো। আমাদের গবেষণার মূল লক্ষ্য হলো বাংলাদেশের প্রত্যন্ত গ্রামে সেরেব্রাল পালসিতে আক্রান্ত শিশুর স্বাস্থ্যের ও তাদের অতি দরিদ্র পরিবারের অর্থনৈতিক/সামাজিক পুঁজির উন্নতিতে সমাজ-ভিত্তিক পূনর্বাসন এবং আয়বৃদ্ধিমূলক কর্মসূচির প্রভাব মূল্যায়ন করা।

গবেষণার পদ্ধতিঃ

গবেষনাটিতে অংশগ্রহনকারীদের নির্বাচন করা হবে দৈবচয়ন ভিত্তিতে এবং অংশগ্রহনকারীদের তিনটি আলাদা দলে ভাগ করা হবে; দল-এ, দল-বি এবং দল-সি। গবেষণায় অংশগ্রহনকারীদের মধ্যে দল-এ এর সদস্যদের জন্য ১২ মাস ব্যাপী গবেষণাকালীন সময়ে প্রতি সপ্তাহে সাপ্তাহিক দলীয় সেশন আয়োজন করা হবে। এই সেশনে সমাজ-ভিত্তিক পুনর্বাসন সেবা প্রদান করা হবে (যেখানে সেরেব্রাল পালসির ব্যবস্থাপনা ও থেরাপির কৌশল শেখানো হবে) এবং পশুপালন ও অন্যান্য আয়বৃদ্ধিমূলক কার্যক্রম সম্পর্কিত আলোচনা করা হবে (আর্থিক অবস্থান উন্নয়নের জন্যে প্রদানকৃত পশু বা গাছের সঠিক পালন ও পরিচর্যা পদ্ধতি, এ সম্পর্কিত সমস্যা, আয় ইত্যাদি)। দল-বি এর সদস্যদের শুধুমাত্র সমাজ-ভিত্তিক পূনর্বাসন সেবা (দল-এ এর মতো করে) প্রদান করা হবে। দল-সি এর সদস্যদের শুধুমাত্র চিকিৎসা ও পুনর্বাসন সম্পর্কিত সাধারণ তথ্য প্রদান করা হবে। দৈবচয়নভিত্তিতে তৈরিকৃত তিনটি দলের সেরেব্রাল পালসিতে আক্রান্ত শিশুর স্বাস্থ্য ও তাদের পরিবারের অর্থনৈতিক/সামাজিক পুঁজি সম্পর্কিত তথ্য গবেষনার শুরুতে, ৬ মাস এবং ১২ মাসকালীন সময়ে মূল্যায়ন করা হবে।

সম্ভাব্য ঝুঁকিঃ

এই গবেষণায় অংশগ্রহনকারীদের জ্ঞাত বা ধারণাকৃত কোন শারীরিক বা আর্থিক ঝুঁকি নেই।

গোপনীয়তাঃ

- যে কোন সময় আপনি নিজেকে এই গবেষণা প্রকল্প থেকে প্রত্যাহার করতে পারবেন। এতে আপনি কোনভাবে ক্ষতিগ্রস্থ হবেন না। প্রধান গবেষণাকারী আপনার দেয়া সকল উত্তরের গোপনীয়তা নিশ্চিত করবেন। যখন এই গবেষণার ফলাফল প্রকাশিত হবে অথবা কনফারেন্সে উপস্থাপিত হবে, তখন আপনার নাম বা আপনাকে চিহ্নিত করা যায় এমন কোন তথ্য ব্যবহার করা হবে না। গবেষণাকাজে অংশগ্রহণের জন্য সম্মতিপত্র পৃথক ভাবে সংরক্ষণ করা হবে।
- পাসওয়ার্ড সুরক্ষিত কম্পিউটারে সকল তথ্য সংরক্ষণ করা হবে এবং তথ্য সংরক্ষণের ফাইল সমূহ পাসওয়ার্ড সুরক্ষিত হবে। তথ্য সম্বলিত সকল নথিপত্র তালাবদ্ধ ক্যাবিনেটে সংরক্ষণ করা হবে। চূড়ান্ত রিপোর্ট জমা দেয়ার পর ছয় বছর পর্যন্ত সংগৃহীত তথ্য সংরক্ষণ করা হবে। প্রকল্প শেষে সকল তথ্য কম্পিউটার থেকে মুছে ফেলা হবে এবং তথ্য সম্বলিত সকল নথিপত্র নষ্ট করে ফেলা হবে।

প্রত্যাহারের অধিকারঃ

- এই গবেষণায় আপনার অংশগ্রহণ সম্পূর্ণ রূপে ঐচ্ছিক। যে সব প্রশ্নের উত্তর প্রদানে আপনি স্বাচ্ছন্দ্য বোধ করবেন, আপনি শুধু মাত্র সেসব প্রশ্নের উত্তর প্রদান করবেন। কোন কারন দর্শানো ছাড়াই যে কোন সময় আপনি নিজেকে এই গবেষণা প্রকল্প থেকে প্রত্যাহার করতে পারবেন।

প্রশ্নঃ

অংশগ্রহণকারী হিসেবে আপনার অধিকার আরও বিস্তারিত ভাবে জানতে, আপনি নিম্নল্লিখিত গবেষকদ্বয়ের সাথে যোগাযোগ করতে পারেন।

গবেষকদ্বয়ের নামঃ


- মাহমুদুল হাসান আল ইমাম
  পদবীঃ রিসার্চ ফিজিওথেরাপিষ্ট,
  ঠিকানাঃ সিএসএফ গ্লোবাল,রোডঃ ২/১,
  বাড়িঃ ৯,ফ্ল্যাট এ-৫ ও বি-৩,বনানী,ঢাকা-১২১৩
  মোবাইলঃ ০১৭৬২০৩২২২৭, ফোনঃ +৮৮০২ ৯৮৫৫৭৩১
  ইমেইলঃ physiomahmud@yahoo.com

- ডা. মানিক চন্দ্র দাস
  পদবীঃ রিসার্চ ফিজিশিয়ান
  ঠিকানাঃ সিএসএফ গ্লোবাল,রোডঃ ২/১,
  বাড়িঃ ৯,ফ্ল্যাট এ-৫ ও বি-৩,বনানী,ঢাকা-১২১৩
  মোবাইলঃ ০১৭৯২৫৯৬৯৪৮, ফোনঃ +৮৮০২ ৯৮৫৫৭৩১
  ইমেইলঃ drmcd19@gmail.com

Appendix-4 Consent Form

Version: 1.0 Date: 12 June 2019

## গবেষণায় অন্তর্ভূক্তির সম্মতিপত্র

গবেষণার বিষয়ঃ বাংলাদেশের গ্রামে বসবাসরত সেরেব্রাল পালসিতে আক্রান্ত শিশু ও তাদের অতি দরিদ্র পরিবারের উপর সমাজ-ভিত্তিক পূনর্বাসন এবং আয়বৃদ্ধিমূলক কর্মসূচির প্রভাব নিরীক্ষা (সাপোর্ট সিপি ট্রায়াল)

আমি, ______________________ (আপনার নাম লিখুন)

______________________ (শিশুর পুরো নাম লিখুন)

সেরেব্রাল পালসিতে আক্রান্ত শিশুর প্রাথমিক পরিচর্যাকারী হিসেবে আমার শিশুকে সাপোর্ট সিপি ট্রায়ালে অন্তর্ভুক্ত করার জন্যে সম্মতি প্রদান করছি।

(পুরো ফর্মে উত্তরের বাক্সে টিক চিহ্ন প্রদান করুন)

আমি সকল তথ্যাবলী স্বজ্ঞানে ও স্বেচ্ছায় পড়েছি এবং বুঝেছি এবং আমার সকল প্রশ্নের উত্তর সন্তোষজনক ভাবে পেয়েছি। আমি বুঝতে পেরেছি যে উল্লেখিত গবেষণা প্রকল্পে আমি ও আমার শিশুর অংশগ্রহন সম্পূর্ণ ঐচ্ছিক এবং গবেষণাকালীন সময়ে যে কোন মুহূর্তে আমি ও আমার শিশু প্রকল্প থেকে নিজেদের নাম প্রত্যাহার করতে পারবো। আমি এই সম্পর্কে সচেতন যে,সমস্ত কিছু পূরণ করার পর এই সম্মতিপত্র তথ্যপত্রের একটি কপি আমার ব্যক্তিগত সংরক্ষনের জন্যে রাখা উচিৎ।

আমি সম্মতি জ্ঞাপন করছি যে,

| | | | | |
|---|---|---|---|---|
| হ্যাঁ | □ | না | □ | সাপোর্ট সিপি ট্রায়ালে আমার/আমার শিশুর/ ব্যক্তি সম্পর্কিত তথ্য সংগ্রহ, রেকর্ড করা এবং স্থায়ী ভাবে সংরক্ষনের জন্যে প্রয়োজন হতে পারে। |
| হ্যাঁ | □ | না | □ | আমার নাম বা চিহ্নিত করা যায় এমন তথ্য গোপন রেখে অন্যান্য তথ্যাবলী সেরেব্রাল পালসি অ্যালায়েন্স রিসার্চ ইনস্টিটিউট কে সরবরাহ বা প্রদান করা হবে। |
| হ্যাঁ | □ | না | □ | সিএসএফ গ্লোবাল এর প্রজেক্ট কর্মকর্তা বা কর্মচারীদের কাছ থেকে ভবিষ্যতে অন্য গবেষণা প্রকল্পে অংশগ্রহনের জন্যে আমন্ত্রন প্রাপ্তির ক্ষেত্রে আমার কোন আপত্তি নেই। |

স্বাক্ষর ______________________ তারিখ ____/____/________

শিশুর সাথে স্বাক্ষরকারীর সম্পর্ক ______________________

---

শুধুমাত্র শিক্ষা বিষয়ক পেশাদারদের জন্য প্রযোজ্য

আমি একজন শিক্ষা বিষয়ক পেশাদার হিসেবে এই মর্মে প্রত্যয়ন করছি যে, আমি প্রাপ্তবয়স্ক/ প্রাথমিক পরিচর্যাকারী এবং/ অথবা দায়িত্বপ্রাপ্ত ব্যক্তিকে পুরো গবেষণা প্রকল্পটি পরিপূর্নভাবে ব্যাখ্যা করেছি এবং আশা করছি তিনি প্রকল্পটি বুঝে সম্পূর্ণ সজ্ঞানে ও স্বাধীনভাবে নিজেকে প্রকল্পে অন্তর্ভূক্তির সম্মতি জ্ঞাপন করেছেন।

স্বাক্ষর ______________________ তারিখ ____/____/________

নাম ______________________ পদবী ______________________
