# Supplementary material for: Supporting Ultra Poor People with Rehabilitation and Therapy among families of children with Cerebral Palsy in rural Bangladesh (SUPPORT CP): Protocol of a randomised controlled trial
Source: PLoS One. 2021 Dec 31;16(12):e0261148. doi: 10.1371/journal.pone.0261148 (PMC8719685; doi:10.1371/journal.pone.0261148)
Supplement: S1 Table — (DOCX) [file pone.0261148.s002.docx]

S1 Table. Schedule of study activities

| **Activity/Group** | **Timeline** | **Integrated Microfinance/livelihood and Community-Based Rehabilitation (IMCBR)** | **Community-Based Rehabilitation (CBR) only** | **Care-as-usual** |
| --- | --- | --- | --- | --- |
| Sampling from existing BCPR cohort | Week 1 | Not assigned | Not assigned | Not assigned |
| Randomisation & enrolment | Week 2 | Yes | Yes | Yes |
| Baseline information (Blinded to outcome assessor) - T0 | Week 3 | Detailed assessment on HRQoL, motor function, communication, and nutritional status of children with CP; mental health, HRQoL, and social capital of their primary caregivers; and socio-economic characteristics, household asset and characteristics, food security, and income and expenditure of families. | Detailed assessment on HRQoL, motor function, communication, and nutritional status of children with CP; mental health, HRQoL, and social capital of their primary caregivers; and socio-demographic characteristics, household asset and characteristics, food security, and income and expenditure of families. | Detailed assessment on HRQoL, motor function, communication, and nutritional status of children with CP; mental health, HRQoL, and social capital of their primary caregivers; and socio-economic characteristics, household asset and characteristics, food security, and income and expenditure of families. |
| Intervention start | Week 4 | IMCBR | CBR only | Care-as-usual (no intervention) |
| Weekly visit | Week 5 to 51 | Yes | Yes | Nil |
| Interim F/U (Blinded outcome assessor) - T1  [6 months] | Week 26 to 29 | Same as previous assessment | Same as previous assessment | Same as previous assessment |
| End of Intervention F/U (Blinded outcome assessor) - T2[12 months] | Week 52 to 53 | Same as previous assessment | Same as previous assessment | Same as previous assessment |
| Long-term F/U (Blinded outcome assessor) - T3  [18 months] | End of intervention F/U + 26  weeks | Same as previous assessment | Same as previous assessment | Same as previous assessment |
