# Supplementary material for: Supporting Ultra Poor People with Rehabilitation and Therapy among families of children with Cerebral Palsy in rural Bangladesh (SUPPORT CP): Protocol of a randomised controlled trial
Source: PLoS One. 2021 Dec 31;16(12):e0261148. doi: 10.1371/journal.pone.0261148 (PMC8719685; doi:10.1371/journal.pone.0261148)
Supplement: S1 Protocol — (PDF) [file pone.0261148.s003.pdf]

Research Expression of Interest
To Asian Institute of Disability and Development (AIDD) Research Committee

(Electronic Format Only)

Submit to: ***disabilityasia@gmail.com***

Date:	02 /05 /2019

**Project Title:**	**Supporting Ultra-Poor People with Rehabilitation and Therapy - a randomized controlled trial among families of children with Cerebral Palsy in rural Bangladesh (SUPPORT CP trial)**
**Person(s) Submitting:** *[add rows if necessary]*	
**Name:**	Professor Gulam Khandaker
**Position:**	Director and Public Health Physician, Central Queensland Public Health Unit
**Contact Details:**	Email: gulam.khandaker@health.nsw.gov.au
**Explain the benefit of this project to people with cerebral palsy or other disabilities:**	This study aims to test a novel integrated intervention to improve health and economic outcomes for children with cerebral palsy (CP) and their families living in poverty. This is the first Randomized Controlled Trial (RCT) of an integrated microfinance and physical rehabilitation program for CP in a low-and middle-income country (LMIC) setting. An integrated health and economic approach to sustainable development is a key focus of our study. We predict that the new intervention will improve the health-related quality of life (HRQoL), motor function, communication and nutritional status of children with CP; mental health, HRQoL and social capital of their parents; and socio-economic status and food security of their families.
**What evidence is available to support the need for this research?** *e.g. discussions with peers/service users, results of other research, literature*	Bangladesh CP Register research findings confirm that poverty is a key contributor to late diagnosis and limited access to early intervention and rehabilitation for children with CP in rural Bangladesh [1]. We also found that even when rehabilitation programs were available access to care was negatively impacted by poverty [1, 2]. In Bangladesh, 97% of families of children with CP live below the poverty line [1]. These families struggle to meet basic needs and their child's rehabilitation often does not feature high on the agenda. Therefore, an integrated approach combining the physical rehabilitation of children with CP and the economic empowerment of their family is required for tangible long-term improvements. Microfinance/livelihood support is an effective tool for improving economic, human (including non-cognitive skills), and social capital of disadvantaged people in LMICs particularly vulnerable groups such as women and children [3]. Microfinance/livelihood support programs can improve health by increasing financial access and service utilization. Combining microfinance with health interventions has yielded promising results in the fields of HIV, malaria and breastfeeding in Africa [4]. We propose a randomized controlled trial to evaluate the effectiveness of an integrated microfinance/livelihood and community-based rehabilitation (IMCBR) program for ultra-poor families of children with CP in rural Bangladesh. We hypothesize that IMCBR will facilitate improved access to capital leading to better income and thus increase the family's investment in physical health overall. Moreover, community-based rehabilitation will provide an opportunity for sharing ideas, information, and developing important non-cognitive skills, such as self-confidence of primary caregivers.
**What impact will this project have on the academic world?**	This will be the first RCT of an integrated microfinance/livelihood and CBR program for children with CP in LMIC settings. Evidence from the study could

	transform approaches to improving wellbeing of children with CP and their families living in extreme poverty.
**Which part of AIDD's research agenda does this project address?**	This project addresses AIDD's objective of producing innovative and scalable social interventions to increase opportunities for children with disabilities to achieve their own goals by leading healthy and economically independent lives.
**Why should AIDD commit to this project?**	CSF Global (www.csf-global.org) is a non-profit pioneering organization in conducting research into childhood disabilities. The organization aims to eliminate preventable causes of childhood impairment in developing countries and ensure that children with disabilities have access to high-quality health, rehabilitation, education, and social inclusion programs to enable their equal participation in society. CSF Global has an unparallel track record in CP research in LMICs, particularly in Bangladesh. CSF Global has established the first CP register in LMIC setting (i.e. the Bangladesh CP Register) and has been leading a comprehensive community-based participatory CP research program in Bangladesh for the past 5 years.
**How do you propose to address this issue? (methodology)**	This will be a cluster RCT comparing three arms: (a) integrated microfinance/livelihood and community-based rehabilitation (IMCBR); (b) community-based rehabilitation (CBR) alone; and (c) care-as-usual (i.e. no intervention). Seven clusters will be recruited within each arm. Each cluster will consist of 10 child-caregiver dyads totaling 21 clusters with 210 dyads. Parents recruited in the IMCBR arm will take part in a microfinance/livelihood program and CP Parent Training Module (PTM), their child with CP will take part in a Goal Directed Training (GDT) program. The programs will be facilitated by specially trained Community Rehabilitation Officers. The CBR arm includes the same PTM and GDT interventions excluding the microfinance/livelihood program. The care-as-usual arm will be provided with information about early intervention and rehabilitation. The assessors will be blinded to group allocation. The duration of the intervention will be 12 months; outcomes will be measured at baseline, and at 6, 12, and 18 months.
**How will this project and its resources and its outcome be funded?**	This study is funded by Cerebral Palsy Alliance Research Foundation (PG02218)
**What is the proposed time frame for this project?**	The duration of this proposed research project is 24 months.
**Who would supervise this project?** *(Provide name & contact details)*	Professor Gulam Khandaker, Director and Public Health Physician, Central Queensland Public Health Unit, Rural and District Wide Service, 82-86 Bolsover Street, Rockhampton, Qld 4700, Australia<br>Phone: +61 02 9845 3061,<br>Email: gulam.khandaker@health.nsw.gov.au

This research proposal may be submitted to an external reviewer with appropriate expertise in the topic.

Please indicate if you have any objection to this process.

• I do not want this proposal submitted for external review:	☐ Yes (✓) No
• I do not want this proposal reviewed by the following person(s):	Not Applicable

References:

1. Khandaker G, Muhit M, Karim T, Smithers-Sheedy H, Novak I, Jones C, et al. Epidemiology of cerebral palsy in Bangladesh: a population-based surveillance study. Dev Med Child Neurol. 2018;61(5):601-9.

2. Power R, Muhit M, Heanoy E, Karim T, Badawi N, Akhter R, et al. Health-related quality of life and mental health of adolescents with cerebral palsy in rural Bangladesh. PLoS One. 2019;14(6):e0217675.

3. Karlan D, Thuysbaert B, Gray B. Credit with Health Education in Benin: A Cluster Randomized Trial Examining Impacts on Knowledge and Behavior. Am J Trop Med Hyg. 2017;96(2):501-510.

4. Linnemayr S, Buzaalirwa L, Balya J, Wagner G. A Microfinance Program Targeting People Living with HIV in Uganda: Client Characteristics and Program Impact. J Int Assoc Provid AIDS Care. 2017;16(3): 254-260.

Research Approval Application
To Asian Institute of Disability and Development (AIDD) Research Committee

(Electronic Format Only)

Submit to: ***disabilityasia@gmail.com***

Date:	02 / 05 /2019

**Project Title:**	**Supporting Ultra-Poor People with Rehabilitation and Therapy - a randomized controlled trial among families of children with Cerebral Palsy in rural Bangladesh (SUPPORT CP trial)**
**Names(s), Titles(s), Qualifications, Dept/Locations and Contact Details**	
**Principal Investigator:**	Professor Gulam Khandaker, Central Queensland Public Health Unit, Central Queensland Hospital and Health Service, Rockhampton, Queensland, Australia.<br>Email: gulam.khandaker@health.nsw.gov.au
**Associates and Co-Investigator:**	Professor Mohammad Muhit, CSF Global, Dhaka, Bangladesh<br><br>Ms Israt Jahan, CSF Global, Dhaka, Bangladesh<br><br>Dr Manik Chandra Das, CSF Global, Dhaka, Bangladesh<br><br>Mr Mahmudul Hassan Al Imam, CSF Global, Dhaka, Bangladesh<br><br>Mx Rosalie Power, Discipline of Child and Adolescent Health, Sydney Medical School, The University of Sydney, Sydney, Australia<br><br>Dr Arifuzzaman Khan, School of Public Health, The University of Queensland, Brisbane, Australia<br><br>Associate Professor Delwar Akbar, School of Business and Law, Central Queensland University, Rockhampton, Queensland, Australia<br><br>Professor Nadia Badawi, Discipline of Child and Adolescent Health, Sydney Medical School, The University of Sydney, Sydney, Australia
**Proposed Date of project commencement:**	Upon HREC approval
**Proposed Duration of Project:**	24 months (Annexure-1)
**Summary of Project:**<br>*(Including impact on people with cerebral palsy/other disabilities and academic world)*	Bangladesh Cerebral Palsy (CP) Register (BCPR) research findings confirm that poverty is a key contributor to late diagnosis and limited access to early intervention and rehabilitation for children with CP in rural Bangladesh. 97% families of children with CP in the country live below the poverty line. Therefore, in low and middle-income countries (LMICs), efforts to improve outcomes for children with CP (including motor, communication and nutritional attainments) should include measures that improve access to health care services along with family economic/social capital.<br><br>We propose a randomized controlled trial (RCT) to evaluate the effectiveness of an integrated microfinance/livelihood and community-based rehabilitation (IMCBR) program for ultra-poor families of children with CP in rural Bangladesh. We predict that IMCBR will facilitate improved access to capital leading to better income and thus increase the family's investment in physical health overall. Moreover, community-based rehabilitation (CBR) will provide an opportunity for sharing ideas, information, and developing important non-cognitive skills, such as self-confidence of primary caregivers.<br><br>We also expect that the research findings will give crucial evidence regarding the effectiveness of such an integrated program in improving wellbeing of children with CP and

	their caregivers, health, and economic outcomes of the families. This will eventually guide the implementing partners to scale up the program using in Bangladesh, and in other LMICs.
**Aims & Significance**<br>*(Include Research Question/s and Hypothesis)*	**Study aims and objectives**<br>The aim of this study is to test the effectiveness of an "Integrated Microfinance/livelihood and CBR program" (IMCBR) targeted to children with CP and their parents from ultra-poor families in rural Bangladesh. The program aims to improve the health-related quality of life (HRQoL), motor function, communication and nutritional status of children with CP; mental health, HRQoL and social capital of their parents; and socio-economic status and food security of their families.<br><br>Our specific objectives are;<br>1. To conduct an RCT with three parallel arms comparing (a) IMCBR, (b) CBR alone, and (c) care-as-usual (i.e. no intervention).<br>2. To measure the effectiveness of IMCBR in improving the HRQoL, motor function, communication, and nutritional status of children with CP from ultra-poor families living in rural Bangladesh.<br>3. To measure the effectiveness of IMCBR in improving mental health, HRQoL, and social capital of parents of children with CP living in rural Bangladesh.<br>4. To measure the effectiveness of IMCBR in improving the socio-economic status of ultra-poor families of children with CP living in rural Bangladesh.<br><br>**Hypothesis**<br>We hypothesize that compared to care-as-usual and CBR alone, the IMCBR program will be more effective in improving HRQoL, motor function, communication, and nutritional outcomes of children with CP from ultra-poor families; and the mental health, HRQoL, and social capital of their primary caregivers; and overall improvement in the socio-economic status of the ultra-poor families of children with CP in rural Bangladesh.
**Justification**<br>*(including literature review and background)*	CP is a group of non-progressive neurological disorders caused by damage to the developing brain [1]. The prevalence and severity of CP are considerably higher in LMICs compared with high-income countries (HICs) [2-4] and diagnosis is likely to be delayed [3]. Early diagnosis of children with CP and access to evidence-based early interventions such as CBR are key to improving the long-term HRQoL [5], motor function [6], cognitive [7], and other health outcomes in children with CP. However, the majority of evidence in this area represents findings from HICs [8, 9]. RCTs testing the effectiveness of CBR programs for children with CP in LMICs are relatively scarce. Moreover, these interventions rarely consider issues pertinent in the lives of children with CP and their families in LMICs such as the impact of living in extreme poverty.<br><br>In LMICs, many families of children with CP live in extreme poverty, which contributes to poor health care access, delayed diagnosis, delayed intervention, overall poor health and wellbeing, and long-term reduced effectiveness of rehabilitation therapies [3, 10-15]. Our last 16 years of research in rural Bangladesh, which led to the development of Bangladesh CP Register (BCPR - first ongoing population-based CP register in LMICs) [16], confirms that in rural Bangladesh diagnosis of CP is delayed and there is limited or no access to evidence-based rehabilitation programs. The average age at diagnosis of CP in Bangladesh is 5 year compared to 1.5 year in HICs [2, 3]. We also found that even when rehabilitation programs were available access to care was negatively impacted by poverty [3, 14]. In Bangladesh, 97% of families of children with CP live below the poverty line [3]. These families struggle to meet basic needs and their child's rehabilitation often does not feature high on the agenda. Therefore, an integrated approach combining the physical rehabilitation of children with CP and the economic empowerment of their family is required for tangible long-term improvements.<br><br>Microfinance/livelihood support is an effective tool for improving economic, human (including non-cognitive skills), and social capital of disadvantaged people in LMICs particularly vulnerable groups such as women and children [17]. Microfinance/livelihood support programs can improve health by increasing financial access and service utilization.

	Combining microfinance with health interventions has yielded promising results in the fields of HIV, malaria, and breastfeeding in Africa [18].<br><br>In addition, non-experimental and quasi-experimental studies testing the effectiveness of integrated health and economic interventions report significant improvements in reproductive and child health, nutrition, and immunization [19, 20]. Non-cognitive skills are considered as important predictors of socio-economic outcomes [21], including the development of small-scale businesses in African context [22, 23]. Moreover, interaction between groups in society reduces prejudice and promotes inter-group cooperation [24-26].<br><br>To be effective, interventions need to be tailored according to the needs of the target population [27]. Influential work by Professor Sir Michael Marmot, Chair of the World Health Organization (WHO) Commission on Social Determinants of Health, and others have demonstrated that socioeconomic factors are important determinants of health [28]. Even in a developed country like the UK, the average life expectancy in poorer areas of Glasgow is about 20 years shorter than that for the rest of the country [29]. This gap can be explained as a direct result of poverty and related social disadvantage. Tangible improvements in overall health status of people living in poverty can only be achieved by focusing on improving both health and economic/social capital. However, to our knowledge, no studies have examined the effectiveness of an integrated health and economic approach for children with CP and their families in LMICs.
**Statement of Outcomes & Benefits**	We believe that this will be the first RCT of an integrated microfinance/livelihood and CBR program for children with CP in LMIC settings. Evidence from the study could transform approaches to improving wellbeing of children with CP and their families living in extreme poverty. The study has been informed by our work on population-based surveillance (i.e. BCPR) and CBR in the local areas, indicating the need for interventions to focus on both health and economic improvement. We will be able to compare the effectiveness of CBR with a new integrated intervention as well as comparing both with standard care practiced in the locality. These data will be scientifically valuable for large scale sustainable program implementation.<br><br>On the other hand, people with disabilities and their families are often excluded from social and economic activities in LMICs. About 97% of the families of children with CP in rural Bangladesh live in extreme poverty [3]; only 31% of ultra-poor families of people with disabilities receive government benefits (in form of social protection) [30]. If the proposed integrated program is proved to be scientifically effective in improving the overall quality of life of children with CP and their caregivers, health, and economic outcomes of the families, the implementing NGO partner plans to scale up the program using existing connections with NGOs and microfinance organizations in Bangladesh, and in other LMICs where CSF Global is research active (e.g., Nepal, Indonesia, and Ghana).
**Method**	
**Design**	**Overview of the study design**<br>This will be a cluster randomized controlled trial comprising three arms. The unit of randomization will be a cluster. Clusters randomized to intervention arms of the trial (i.e. IMCBR and CBR arms) will receive interventions following the protocol outlined in later sections. The interventions will be provided to dyads consisting of children with CP and their primary caregivers. Whereas, clusters randomized to care-as-usual arm of the trial will not receive any active intervention. (Annexure-2)
**Participant Inclusion/ Exclusion Criteria**	**Inclusion/exclusion criteria**<br>Participants will be considered eligible for participation based on the following criteria:<br>1. Children with CP aged ≤5 years, classified as from an ultra-poor family (i.e. per day per capita income <1.90 USD; [31]) and registered in the BCPR. The BCPR registers children with CP following the case definition adopted from the Surveillance of CP in Europe (SCPE) and the Australian CP Register (ACPR) [3].

	2. Primary caregiver (e.g. parent, sibling, grandparent of the child with CP)<br>3. Primary caregiver has the capacity to give informed consent and is willing to take part in the study including microfinance/livelihood arm along with their child with CP.<br><br>Participants will be considered ineligible for participation based on the following criteria:<br>1. Currently in receipt of microfinance/livelihood support from another source.<br>2. Currently participating in any other clinical trial or intervention program.<br><br>**Sample size calculation**<br>The sample size for this cluster RCT has been computed based on methods described in Donner et al. [32]. We will recruit seven clusters in each arm, and each cluster will consist of 10 CP children with CP- primary caregivers dyads totaling 21 clusters of 210 dyads. Based on our pilot data and existing literature we predict 35% improvement of HRQoL in the IMCBR group, 20% improvement in the CBR alone group, and 5% improvement in the care-as-usual group. With a sample size of 210 dyads, this study will have 80% statistical power to detect these effects (two-sided α-value=0.05) [9]. Power calculation takes into account up to 20% sample attrition by the end of the trial. A homogeneous study population will allow us to balance randomization considering intra-cluster correlation of 0.5 and coefficient of variation in cluster size of 0.5.<br><br>**Cluster formation and randomization**<br>The study will include 21 clusters randomized to three arms (7 clusters each) and allocated by a 1:1:1 ratio. Each cluster will come from a 'Mouza', the smallest public administrative unit in Bangladesh comprising of approximately five villages (~8,250 people) and will include 10 CP child-primary caregiver dyads. In order to minimize 'contamination' of the intervention types, clusters will be separated from each other by buffering areas comprising villages not taking part in the study. Cluster margins will be configured so that they align with natural divisions that separate residents in the community (e.g., rivers). The randomization process will be executed following the standard process.
**Recruitment**	The study will utilize the Bangladesh CP Register (BCPR) as a sampling frame for participant recruitment. The BCPR is an ongoing surveillance of children with CP commenced in 2015 [3], and currently being operated in four districts of Bangladesh. Between 2015 and 2019, 1125 children with CP have been registered into the BCPR from Shahjadpur (i.e. the study site). As part of this RCT, dyads of children with CP and their primary caregivers who meet the inclusion criteria will be recruited and assigned to different arms of the study.<br>The BCPR findings show that 34.2% of the children registered are aged <5 years and 97% of the families are ultra-poor (i.e. per day per capita income <1.90 USD) [3]. Considering the number of registrants from Shahjadpur (i.e.~1125), there are ~373 children eligible to participate in the study. Therefore, recruitment of 210 children and their primary caregiver in the trial (<60% of the available pool) is feasible. Sociodemographic, economic, and health data of these children and their families are already recorded in the BCPR allowing quick identification and recruitment. All families enrolled in the BCPR have also been mapped using Geographic Information System (GIS). (Annexure-3)
**Intervention**	**Arm A- Integrated Microfinance/livelihood and Community-Based Rehabilitation (IMCBR)**<br><br>Participants randomized to the IMCBR arm will be supported to create microfinance/livelihood groups (10 participant-pairs per group). The groups will be formed voluntarily along geographical boundaries to facilitate participation, retention, and meeting logistics. Each cluster will meet weekly to discuss microfinance/livelihood activities (e.g. weekly credit collection and troubleshooting) (90 minutes) and for CBR with children with CP comprising early intervention and primary caregiver's education (90 minutes).<br><br>*A.1 Microfinance/livelihood program details*<br>Group meetings will be organized with members of each cluster to discuss (i) details of the program, (ii) potential benefits and challenges of participation in the program, and (iii) motivations for participation. Participants of each cluster can then apply for a loan/livelihood

	support; a minimum 10% deposit of the requested loan/livelihood support amount in the form of savings is required and is admissible immediately after cluster formation. Once the application is completed, loan approval and disbursement of the loan will occur approximately within one week.<br>Amount, return cycle of loan and investment areas: Each of the ultra-poor families will receive a loan/livelihood support amounting/equivalent to ~100-300AUD at 12% flat interest rate. The return cycle will be one year with a weekly repayment schedule. Common investment areas for the ultra-poor loan will be for goat or cattle rearing, seeds for agriculture, home-based weaving, and handicraft business [33]. Using a structured tool (Annexure-4) a comprehensive needs assessment will be conducted to guide the decision of livelihood support to be provided.<br><br>*A.2 CBR*<br>There will be two major components of the CBR program, which will occur during cluster meetings following the microfinance/livelihood portion.<br><br>a. Goal Directed Training (GDT): Community-based GDT focused on motor learning will be conducted with children with CP and their primary caregivers. GDT is an activity-based approach to therapy where meaningful, client-selected (i.e. caregivers of children with CP) goals are used to provide opportunities for problem-solving and to indirectly drive the movements required to successfully meet task demands [34]. Evidence from a meta-analysis shows that GDT based interventions are highly effective and should be the gold standard treatment for CP [35]. In this study, GDT will be delivered by child's primary caregiver (participating parent).<br><br>*b. Parents Training Module (PTM):* Primary caregivers will participate in PTM to learn basic therapeutically correct skills for the day-to-day care and support of their child with CP embedded in the principles of GDT. This study will follow the PTM 'Getting to know cerebral palsy' which includes 10 modules and covers topics; introduction to CP, evaluating your child, positioning and carrying, communication, everyday activities, feeding your child, play, disability in your local community, running your own parent support group, and assistive devices and resources [36].<br><br>Specially trained Community Rehabilitation Officers (CRO's) will facilitate each of the cluster meetings (both microfinance/livelihood and CBR activities). The CROs will facilitate microfinance/livelihood discussions and lead the GDT and PTM sessions with the aim to upskill primary caregivers so that they can continue to deliver GDT independently at home. Prior to implementation of the RCT, CROs will take part in a 5-day training by Research Physiotherapist. The CRO training will cover the following areas; (i) socio-cultural considerations in working with primary caregivers of children with CP, (ii) introduction to microfinance/livelihood support program management, (iii) developmental milestones and development in children with CP, (iv) therapeutic principles, (v) GDT, (vi) activity focused therapies, (vii) basic speech development strategies, (viii) contraindications of therapies, (ix) research ethics, and (x) child rights.<br><br>**Arm B- CBR alone**<br>Participants from clusters randomized to this arm will attend a weekly rehabilitation session at a local focal point (preferably one of the group members' home). Each session will last for 90 minutes and will be identical to the CBR component of IMCBR (discussed above); however, the microfinance/livelihood component will not be provided.<br><br>**Arm C- Care-as-usual (i.e. no intervention)**<br>This group will not receive any active intervention. Once children with CP are identified and randomized into clusters, the 'care-as-usual' participants will be provided with basic education on early intervention and rehabilitation and will be encouraged to access healthcare via usual routes, which typically include treatment in government hospitals. The proposed intervention schedules for all three arms have been summarized in Annexure-5.

	**Concurrent interventions**<br>Children with CP from all three study arms will be able to continue accessing need-based medical and therapy support from other sources as per their family's preferences. Frequency and duration of access to local medical/therapy services will be recorded during follow-up assessments and included in analysis.
**Outcome measures**	**Outcome measures**<br>The following outcomes will be measured at baseline, at 6 months, 12 months, and 18 months. The tools that will be used to measure outcomes have been outlined in Annexure-6.<br><br>***Primary outcomes***<br>1. HRQoL of children with CP<br><br>***Secondary and exploratory outcomes***<br>1. Motor function of children with CP<br>2. Communication function of children with CP<br>3. Nutritional status of children with CP<br>4. Mental health of primary caregivers of children with CP<br>5. HRQoL of primary caregivers of children with CP<br>6. Social capital of primary caregivers of children with CP<br>7. Family socio-economic status and food security
**Data management and analysis**	Data will be collected using paper-based forms. Research data will be anonymized and stored securely and separately for participant identifiable information. This will include monitoring secure data transfer from field to the central office, data entry and quality control of completed forms, querying of missing or invalid data, and archiving of physical forms. Data will be collected using validated questionnaires. Data will be entered into PCs using Microsoft Access or SQL Server as the relational database engine. Any error identified during data entry or in data cleaning will be logged for field supervisor assessment and will be resolved after proper field verification. The physical data will be stored for 7 years in a locked cabinet at head office of CSF Global based in Bangladesh as per national and international guidelines.<br><br>Intention-to-treat analysis will compare improvements in primary and other outcomes between groups controlling for baseline measures. Descriptive statistics (frequencies, means and 95% confidence intervals) will be used to describe the sample at baseline and post-intervention. Hypothesis testing will be done using appropriate statistical procedures (e.g., Chi-square test, Fisher's exact test, paired t test, ANOVA) based on the distribution nature and type of data. To account for the intra-cluster correlation in calculating 95% CI and p-value, we will use Sandwich estimate of standard error. Baseline characteristics will also be compared and adjusted using appropriate regression models. All analyses will be conducted using STATA 15, with the significance level set at $p<0.05$. Data visualization will be done using R studio/GraphPad Prism 7.
**Dissemination of Results & Recommendation**	The study findings will be shared with local and national Micro Finance Institutions and non-governmental organizations. We also aim to publish the trial findings in peer-reviewed journals and present at national and international conferences/workshops. Findings from this study, including key learnings, will be shared with stakeholders including rehabilitation practitioners working with children with CP in Bangladesh and other LMICs. The findings will also be shared with the participating parents of children with CP in the proposed study sites.
**Allocation of Resources (AIDD Staff Only)**	
**Staff Time**	
**Other – give details**	
This research proposal may be submitted to an external reviewer with appropriate expertise in the topic.<br><br>Please indicate if you have any objection to this process.	

• I do not want this proposal submitted for external review:	☐ Yes ☑ No
• I do not want this proposal reviewed by the following person(s):	Not applicable

References:

1. Rosenbaum P, Paneth N, Leviton A, Goldstein M, Bax M. A report: the definition and classification of cerebral palsy April 2006. Dev Med Child Neurol Suppl. 2006;109:8-14.

2. Australian Cerebral Palsy Register. Report of the Australian Cerebral Palsy Register, birth years 1995–2012 [Internet]. Sydney: Australian Cerebral Palsy Register; 2018 p.18-21. Available from: https://cpregister.com/wp-content/uploads/2019/02/Report-of-the-Australian-Cerebral-Palsy-Register-Birth-Years-1995-2012.pdf

3. Khandaker G, Muhit M, Karim T, Smithers-Sheedy H, Novak I, Jones C, et al. Epidemiology of cerebral palsy in Bangladesh: a population-based surveillance study. Dev Med Child Neurol. 2018;61(5):601-9.

4. Kakooza-Mwesige A, Andrews C, Peterson S, Wabwire Mangen F, Eliasson AC, Forssberg H. Prevalence of cerebral palsy in Uganda: a population-based study. Lancet Glob Health. 2017;5:e1275–82.

5. Bongo PP, Dziruni G, Muzenda-Mudavanhu C. The effectiveness of community-based rehabilitation as a strategy for improving quality of life and disaster resilience for children with disability in rural Zimbabwe. Jamba. 2018;10(1):1-0.

6. Dambi JM, Jelsma J. The impact of hospital-based and community based models of cerebral palsy rehabilitation: a quasi-experimental study. BMC Pediatr. 2014;14 (1):301.

7. Bao X, Sun S, Yu R, Sun J. Early intervention improves intellectual development in asphyxiated newborn infants. Intervention of Asphyxiated Newborn Infants Cooperative Research Group. Chin Med J (Eng).1997;110:875–878.

8. Novak I, Morgan C, Adde L, Blackman J, Boyd RN, Brunstrom-Hernandez J, et al. Early, accurate diagnosis and early intervention in cerebral palsy: advances in diagnosis and treatment. JAMA pediatr. 2017;171(9):897-907.

9. Tsoi WS, Zhang LA, Wang WY, Tsang SK, Lo SK. Improving quality of life of children with cerebral palsy: a systematic review of clinical trials. Child Care Health Dev. 2012;38(1): 21-31.

10. Jahan I, Muhit M, Karim T, Smithers-Sheedy, Novak I, Jones C, et al. What makes children with cerebral palsy vulnerable to malnutrition? Findings from the Bangladesh cerebral palsy register (BCPR). Disabil Rehabil. 2018;41(19):2247-54.

11. Akhter R, Hassan NM, Martin EF, Muhit M, Haque MR, Smithers-Sheedy H, et al. Risk factors for dental caries among children with cerebral palsy in a low-resource setting. Dev Med Child Neurol. 2017;59(5):538-543.

12. May P, Smithers-Sheedy H, Muhit M, Cumming R, Jones C, Booy R, et al. Immunisation status of children with cerebral palsy in rural Bangladesh: results from the Bangladesh Cerebral Palsy Register (BCPR). Infect Disord Drug Targets. 2018;18:(1).

13. Khan A, Ashher F, Karim T, Fatema A, Jahan I, Muhit M, et al. Immunization status of mothers of children with cerebral palsy in rural Bangladesh. Infect Disord Drug Targets. 2018;18:1-6.

14. Power R, Muhit M, Heanoy E, Karim T, Badawi N, Akhter R, et al. Health-related quality of life and mental health of adolescents with cerebral palsy in rural Bangladesh. PLoS One. 2019;14(6):e0217675.

15. Khandaker G, Muhit M, Rashid H, Khan A, Islam J, Jones C, et al. Infectious causes of childhood disability: results from a pilot study in rural Bangladesh. J Trop Pediatr. 2014;60(5):363-9.

16. Khandaker G, Smithers-Sheedy H, Islam J, Alam M, Jung J, Novak I, et al. Bangladesh Cerebral Palsy Register (BCPR): a pilot study to develop a national cerebral palsy (CP) register with surveillance of children for CP. BMC Neurol. 2015;15(175):173.

17. Karlan D, Thuysbaert B, Gray B. Credit with Health Education in Benin: A Cluster Randomized Trial Examining Impacts on Knowledge and Behavior. Am J Trop Med Hyg. 2017;96(2):501-510.

18. Linnemayr S, Buzaalirwa L, Balya J, Wagner G. A Microfinance Program Targeting People Living with HIV in Uganda: Client Characteristics and Program Impact. J Int Assoc Provid AIDS Care. 2017;16(3): 254-260.

19. Saggurti N, Atmavilas Y, Porwal A, Schooley J, Das R, Kande N, et al., Effect of health intervention integration within women's self-help groups on collectivization and healthy practices around reproductive, maternal, neonatal and child health in rural India. PLoS One. 2018;13(8):e0202562.

20. Hamad R, Fernald LC. Microcredit participation and nutrition outcomes among women in Peru. J Epidemiol Community Health. 2012;66(6):e1.

21. Heckman JJ, Kautz T. Hard evidence on soft skills. Labour Econ. 2012;19(4):451-464.

22. Berge LI, Bjorvatn K, Pires AJ, Tungodden B. Competitive in the lab, successful in the field? J Econ Behav Organ. 2015;118:303-317.

23. Campos F, Frese M, Goldstein M, Lacovone L, Johnson HC, McKenzie D, et al. Teaching personal initiative beats traditional training in boosting small business in West Africa. Science. 2017;357(6357):1287-1290.

24. Rao G. Familiarity Does Not Breed Contempt: Diversity, Discrimination and Generosity in Delhi Schools. Am Econ Rev. 2019; 103(3):774-809.

25. Boisjoly J, Duncan GJ, Kremer M, Levy DM, Eccles J. Empathy or Antipathy? The Impact of Diversity. Am Econ Rev. 2006;96(5):1890-1905.

26. Burns J, Corno l, La Ferrea E. Interaction, prejudice and performance. Evidence from South Africa. Working Paper. 2015. Available from http://www.ne.su.se/polopoly_fs/1.226646.1425460291!/menu/standard/file/BurnsCornoLaFerrara_BREAD.pdf

27. Kreuter MW, Strecher VJ, Glassman B. One size does not fit all: the case for tailoring print materials. Ann Behav Medicine. 1999;21(4):276.

28. Marmot M, Friel S, Bell R, Houweling TAJ, Taylor S, Commission on Social Determinants of Health. Closing the gap in a generation: health equity through action on the social determinants of health. Lancet. 2008;372:1661-1669.

29. Whyte B. Glasgow: health in a changing city. A descriptive study of changes in health, demography, housing, socioeconomic circumstances and environmental factors in Glasgow over the last 20 years [Internet]. Glasgow: Glasgow Centre for Population Health; 2016. p.17-51. Available from: https://www.bl.uk/collection-items/glasgow-health-in-a-changing-city-a-descriptive-study-of-changes-in-health-demography-housing-socioeconomic-circumstances-and-environmental-factors-over-the-last-20-years

30. MIX [Internet]. Washington DC: Microfinance Information Exchange; C2014 [cited 2019 Aug 27]. Available from: http://www.themix.org

31. BRAC. An end in sight for ultra-poverty: Scaling up BRAC's graduation model for the poorest. Briefing note 1: Ending extreme poverty. Dhaka: BRAC; 2013. p.1-8. Available from: http://www.brac.net/sites/default/files/BRAC%20Briefing%20-%20TUP.pdf

32. Donner A, Klar N. Design and analysis of cluster randomization trials in health research. Hoboken, NJ, USA: John Wiley & Sons, Inc; 2010.

33. Hossain MK. Measuring the impact of BRAC microfinance operations: A case study of a village. Int Bus Res. 2012;5(4):112-23. doi:10.5539/ibr.v5n4p112

34. Mastos M, Miller K, Eliasson AC, Imms C. Goal-directed training: linking theories of treatment to clinical practice for improved functional activities in daily life. Clin Rehabil. 2007;21(1):47-55.

35. Sakzewski L, Ziviani J, Boyd RN. Efficacy of upper limb therapies for unilateral cerebral palsy: a meta-analysis. Pediatr. 2014;133(1):e175-204.

36. Zuurmond M, O'Banion D, Gladstone M, Carsamar S, Kerac M, Baltussen M, et al. Evaluating the impact of a community-based parent training programme for children with cerebral palsy in Ghana. PLoS One. 2018;13(9):e0202096.

Annexure-1: Operational plan and timeline of SUPPORT CP Trial

Phases	Sr No.	Activity Name	Month1-Month2	Starting date (proposed): Month3-Month4	Month5-Month8	Month9-Month10	Month11-Month14	Month15-Month16	Month17-Month20	Month21-Month22	Month23-Month24	Designated Person
Preparatory	1	Ethics application										Trial Coordinator
	2	Trial registration										Senior Research Fellow
	3	Data collection materials (Questionnaire, PIS, Consent form)										Research Program Manager, Research Physiotherapist, Research Physician
	4	Project menus development										Project Officer
	5	Reporting/monitoring template										Research Program Manager, Trial Coordinator
	6	SUPPORT CP field team formation										Principal Investigator
	7	Eligible participant selection for all 3 arms										Field Project Coordinator
	8	Groups formation and groups' leaders selection										Field Project Coordinator
	9	Infrastructural set up for community-based rehabilitation (CBR)										Field Project Coordinator
	10	Needs assessment for Arm A										Project Officer
	11	Budgeting										Research Program Manager, Trial Coordinator
	12	Goal Directed Training for Community Rehabilitation Officer										Research Physiotherapist, Research Speech and Language Therapist
	13	Training to data collection team										Research Program Manager, Research Physiotherapist

Annexure-1: Operational plan and timeline of SUPPORT CP Trial

Phases	Sr No.	Activity Name	Month1-Month2	Starting date (proposed): Month3-Month4	Month5-Month8	Month9-Month10	Month11-Month14	Month15-Month16	Month17-Month20	Month21-Month22	Month23-Month24	Designated Person
	14	Good Clinical Practice training for study team members										Senior Research Fellow
Implementation	15	Medical assessment		Baseline (BL)-Arm A: 13-15 Dec 2019		Midline (ML)-Arm A: 13-16 Jun 2020		Endline (EL)-Arm A: 12-14 Dec 2020		6m follow up (FU) -Arm A: 13-16 Jun 2021		Research Physician, Research Physiotherapist, Research Speech and Language Therapist
				BL-Arm B: 21-24 Dec 2019		ML-Arm B: 20-23 Jun 2020		EL-Arm B: 19-21 Dec 2020		6m FU--Arm B: 20-23 Jun 2021		
				BL-Arm C: 11-15 Jan 2020		ML-Arm C: 11-15 Jul 2020		EL-Arm C: 9 -12 Jan 2021		6m FU-Arm C: 11-15 Jul 2021		
	16	Product purchase and distribution		1-9 Jan 2020								Project Officer, Office Assistant
	17	Data entry and record keeping		BL: 16-22 Jan 2020		ML: 16-22 Jul 2020		EL: 13-19 Jan 2021		6m FU: 16-22 Jul 2021		Data Management Officer
	18	Weekly CBR session		Commencement date for Arm A & B: 18 Jan 2020								Community Rehabilitation Officer
	19	Monitoring & record keeping-CBR session			Weekly & Monthly	Weekly & Monthly	Weekly & Monthly					Community Rehabilitation Officer, Field Project Officer, Trial Coordinator
	20	Monitoring & record keeping-Livelihood impact			Fortnightly & Monthly	Fortnightly & Monthly	Fortnightly & Monthly					Community Rehabilitation Officer, Field Project Officer, Project Officer, Trial Coordinator

Annexure-1: Operational plan and timeline of SUPPORT CP Trial

Phases	Sr No.	Activity Name	Month1-Month2	Starting date (proposed): Month3-Month4	Month5-Month8	Month9-Month10	Month11-Month14	Month15-Month16	Month17-Month20	Month21-Month22	Month23-Month24	Designated Person
**Reporting**	21	Data analysis and reporting										Research Program Manager, Trial Coordinator
	22	Donor reporting										Research Program Manager

Anexure-2: Study design (Consolidated Standards of Reporting Trials diagram)

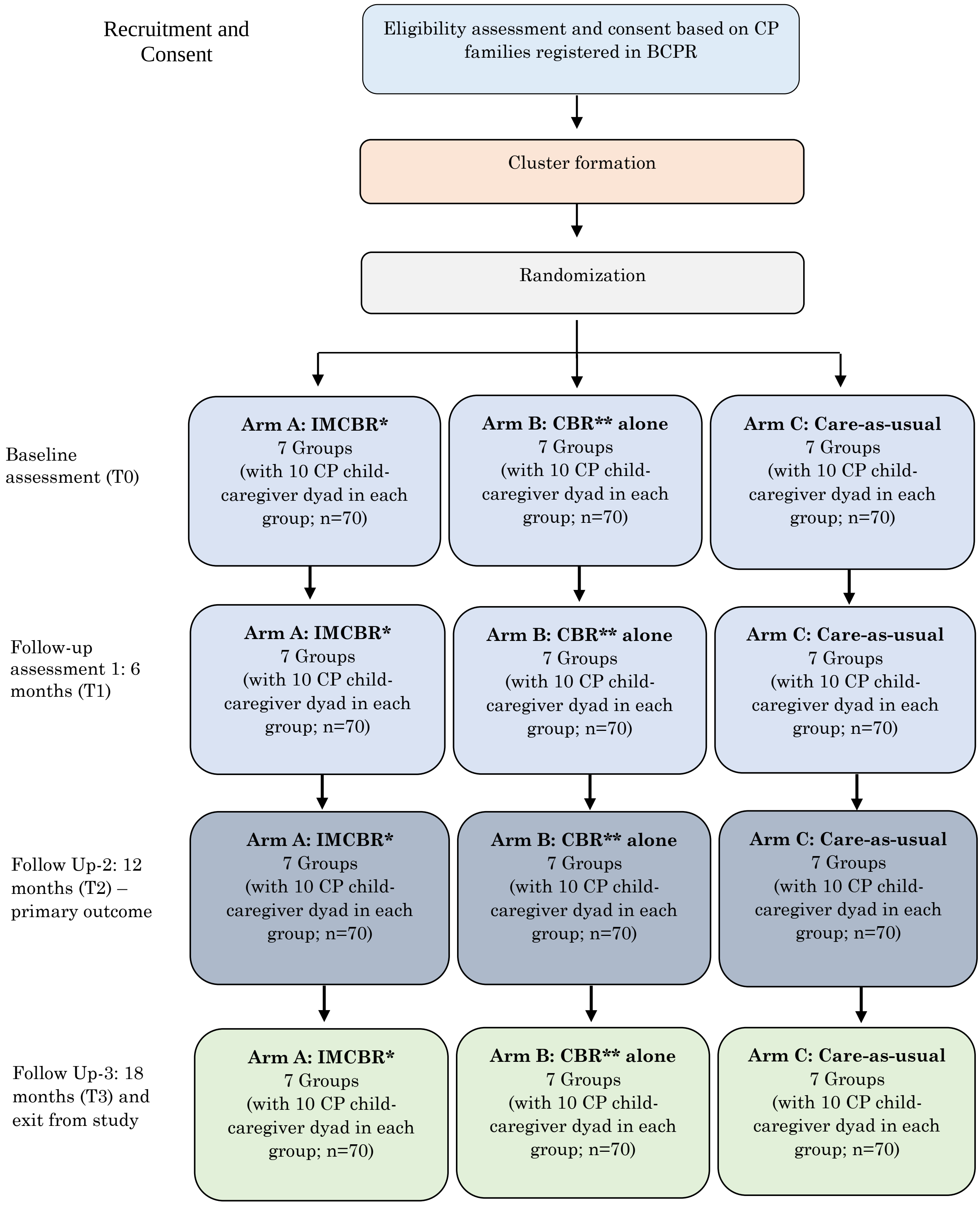

*IMCBR stands for integrated microfinance/livelihood and community-based rehabilitation
**CBR stands for community-based rehabilitation

Annexure-3: Geographic Information System (GIS) map of the study area and eligible clusters

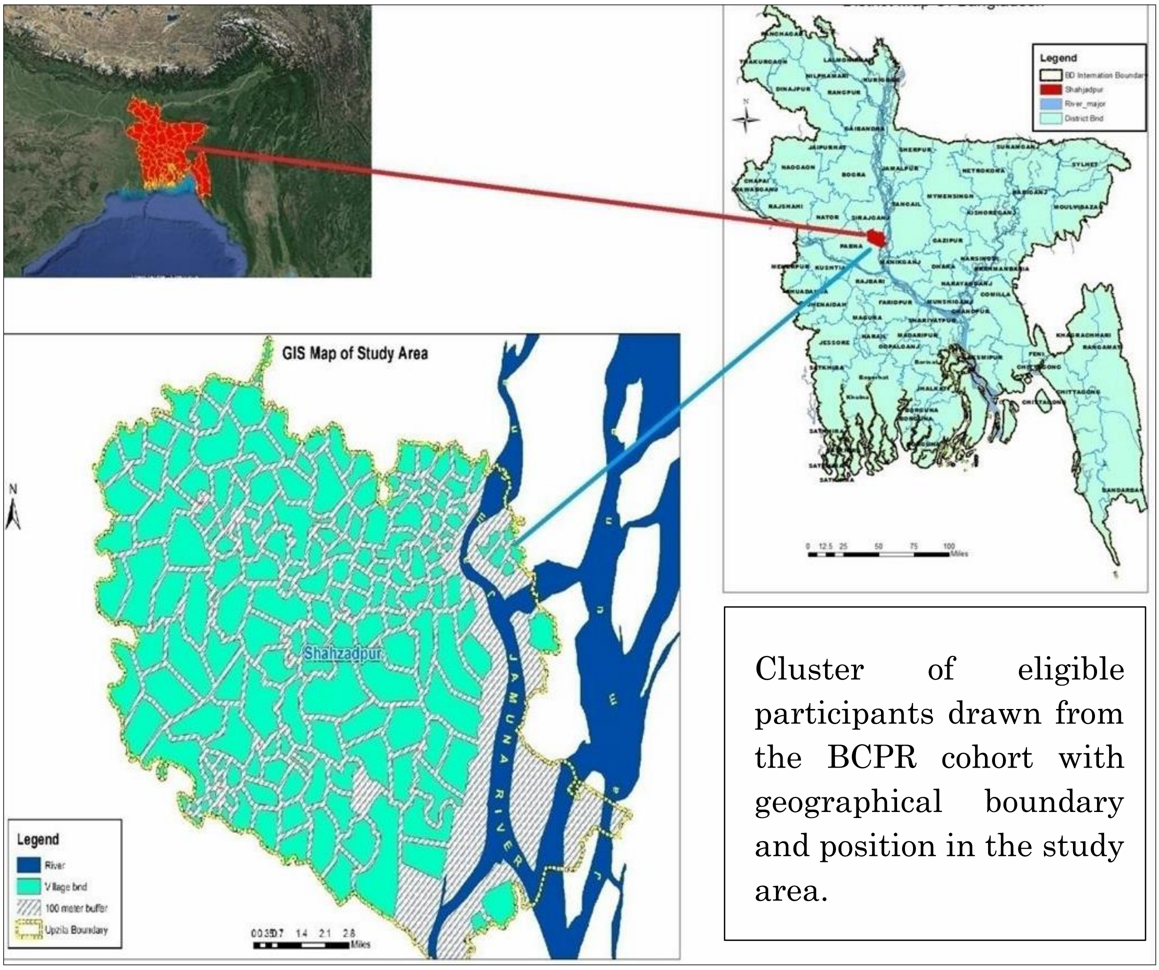

Cluster of eligible participants drawn from the BCPR cohort with geographical boundary and position in the study area.

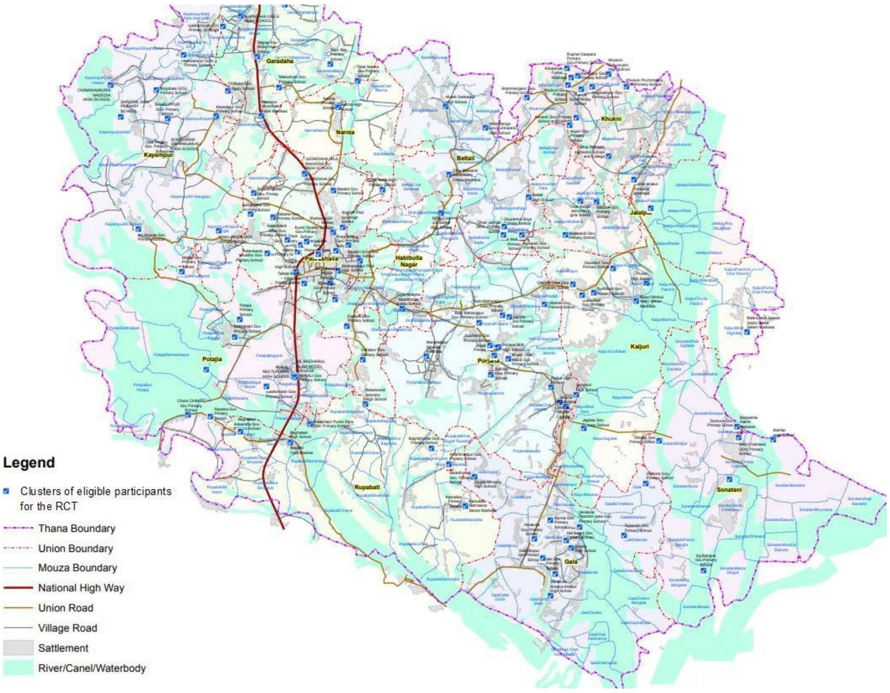

Annexure-4: Needs Assessment Form

CSF Global
Support CP Trial
<u>Needs Assessment Form</u>

1. Participant name:

2. Father name:

3. Mother name:

4. Spouse name:

5. Name of child with CP: Age: Year:

6. Types of CP:

7. BCPR Registration number:

8. Nationality: Religion:

9. Occupation:

10.Telephone/Mobile:

11.Permanent Address: Village: Post office:

Union: Thana/ Upazila:

District:

12.Present Address: Village: Post office:

Union: Thana/ Upazila:

District:

13. National Identity Card Number:

14. Client's educational qualifications: a) Primary b) Secondary c) Higher secondary d) Graduation e) Masters

15. Analysis of participant's family and social status:

- Population in the family: Male: Female:
- Number of employed men: ; Number of employed women:
- Number of children: boys: girls:
- Number of children with CP:
- Whether there is a police case in the name of any family member: Yes / No (Tick)
  If the answer is yes or if there is a case, describe it:
- Whether any family member is involved in any illegal activity: Yes/ No (Tick)

Assessment of family status:

SL	Subject	Evaluation
1	Joint family / nuclear family	
2	Total number of family members	
3	Number of emale	
4	Polygamy	Yes/No
5	Child marriage	Yes/No
6	Number of incurable patients	
7	Number of pregnant women	
8	Number of retired old people	
9	Primary caregiver of the CP child	
10	Whether women of the family have control over wealth/money?	Yes/No
11	Whether woman can make decisions?	Yes/No
12	Number of women leading any business	
13	Number of oppressed and abused women	
14	Number of women can travel independently	

Assessment of economic status:

SL	Subject	Evaluation
15	Source of income	
16	Total number of earners	
17	Daily income	Tk
18	Daily expenses	Tk
19	Monthly expenses (for food)	Tk
20	Monthly expenses (for cloth)	Tk
21	Monthly expenses (for education)	Tk
22	Monthly expenses (for medical/treatment)	Tk
23	Monthly expenses (for purchase of land)	Tk
24	Monthly expenses (others)	Tk
25	Monthly savings	Tk
26	Do you have bank savings?	Yes/No
27	Do you have savings in a cooperative society?	Yes/No
28	Have you taken any microcredit?	Yes/No

Assessment of eating habit:

SL	Subject	Evaluation
29	How many meals do you take in a day?	
30	How many times do you eat rice in a day?	
31	How often do you eat fish (per day)?	
32	How often do you eat meat (per day)?	
33	How often do you eat vegetables (per day)?	
34	How often do you eat pulses (per day)?	
35	How often do you eat eggs (per day)?	
36	Do you use water from tube-wells?	Yes/No
37	Do you drink water from tube-wells?	Yes/No
38	What type of stove do you use?	Gas/Wood

Assessment of educational status:

SL No.	Subject	Evaluation
39	Number of non-formal learners	
40	Number of primary school going students	
41	Number of secondary school going students	
42	Number of college going students	
43	Number of university going students	
44	Number of people without education	

Assessment of real-estate, land and other resources

SL	Subject	Evaluation
45	Number of straw houses	
46	Number of tin houses	
47	Number of pucca houses	
48	Number of rooms	
49	Amount of abode	
50	Amount of abode land	
51	Amount of cultivable land	
52	Amount of uncultivable land	
53	Do you have a personal/shared pond?	Yes/No
54	Do you have a personal tube-well?	Yes/No
55	Number of trees (fruit / wood tree)	
56	Number of cows/goats	
57	Number of ducks/chickens/turkeys	
58	Number of pigeons	
59	Do you have a commercial animal farm?	Yes/No
60	Do you have a separate barn?	Yes/No
61	Do you have a separate house for duck / chicken?	Yes/No
62	Do you have a separate house for pigeons?	Yes/No

Assessment of social status:

SL	Subject	Evaluation
63	Do you get invitations to social events regularly?	Yes/No
64	Do you get entertained by neighbours?	Yes/No
65	Do neighbours visit your home regularly?	Yes/No
66	Is your opinion taken into account in social arbitration?	Yes/No

Annexure-5: Schedule of study activities

Activity/Group	Timeline	Integrated Microfinance/livelihood and Community-Based Rehabilitation (IMCBR)	Community-Based Rehabilitation (CBR) only	Care-as-usual
Sampling from existing BCPR cohort	Week 1	Not assigned	Not assigned	Not assigned
Randomization & enrolment	Week 2	Yes	Yes	Yes
Baseline information (Blinded to outcome assessor) - T0	Week 3	Detailed assessment on HRQoL, motor function, communication, and nutritional status of children with CP; mental health, HRQoL, and social capital of their primary caregivers; and socio-economic characteristics, household asset and characteristics, food security, and income and expenditure of families.	Detailed assessment on HRQoL, motor function, communication, and nutritional status of children with CP; mental health, HRQoL, and social capital of their primary caregivers; and socio-demographic characteristics, household asset and characteristics, food security, and income and expenditure of families.	Detailed assessment on HRQoL, motor function, communication, and nutritional status of children with CP; mental health, HRQoL, and social capital of their primary caregivers; and socio-economic characteristics, household asset and characteristics, food security, and income and expenditure of families.
Intervention start	Week 4	IMCBR	CBR only	Care-as-usual (no intervention)
Weekly visit	Week 5 to 51	Yes	Yes	Nil
Interim F/U (Blinded outcome assessor) - T1 [6 months]	Week 26 to 29	Same as previous assessment	Same as previous assessment	Same as previous assessment
End of Intervention F/U (Blinded outcome assessor) - T2[12 months]	Week 52 to 53	Same as previous assessment	Same as previous assessment	Same as previous assessment
Long-term F/U (Blinded outcome assessor) - T3 [18 months]	End of intervention F/U + 26 weeks	Same as previous assessment	Same as previous assessment	Same as previous assessment

Annexure-6: Case Record Form

**A. Basic information**	**Study ID:**								

**Stage of data collection;**	**Study Arm**	
☐ Baseline<br>☐ Midline<br>☐ End line	☐ Arm A<br>☐ Arm B<br>☐ Arm C	**Name of the child:**<br>**Sex of the child:** Male=1, Female=2<br>**Date of Birth:** __ __/__ __/__ __ __ __(dd/mm/yyyy)<br>**Date of Assessment:** __ __/__ __/__ __ __ __(dd/mm/yyyy)

B. Contact details of primary caregiver

1. Name:		2.Relationship with child:	
3. District:	4. Subdistrict:	5. Union/Ward:	6. Village:
7. Postcode:	8. Nearest geographical landmark:		
9. Phone/Mob:		10. Phone/Mob (alternate):	

C. Birth parent details

**Mother**		
1. Name:	2. Age at birth of the child (years):	
3. **Educational level:**<br>No education=1, Primary incomplete=2, Completed primary=3, Secondary incomplete=4, Completed secondary= 5, Higher secondary completed= 6, More than higher secondary=7, Other=8, specify______		
4. **Occupation:**<br>Professional/technical=1, Business=2, Factory worker=3, Skilled labour=4, Unskilled labour=5, Farmer/agricultural worker=6, Poultry, cattle raising=7, Home based manufacturing=8, Domestic servant= 9, Housewife=10, Other=11 **(specify)**		
**Father**		
5. Name:	6. Age at birth of the child (years):	
7. **Educational level:**<br>No education=1, Primary incomplete=2, Completed primary=3, Secondary incomplete=4, Completed secondary= 5, Higher secondary completed= 6, More than higher secondary=7, Other=8 **(specify)**		
8. **Occupation:**<br>Professional/technical=1, Business=2, Factory worker=3, Skilled labour=4, Unskilled labour=5, Farmer/agricultural worker=6, Poultry, cattle raising=7, Home based manufacturing=8, Domestic servant= 9, Other =10 **(specify)**		

D. Household information

D1. Housing characteristics

1.1 **Main material of the floor:**<br>Earth/Sand=1, Wood Planks=2, Palm/Bamboo=3, Parquet Or Polished Wood=4, Ceramic Tiles=5, Cement=6, Carpet=7, Other=8 **(specify)**	
1.2 **Main Material of The Roof:**<br>No Roof =1, Thatch/Palm Leaf =2, Palm/Bamboo=3, Wood Planks =4, Cardboard=5, Tin=6, Wood=7, Ceramic Tiles=8, Cement=9, Roofing Shingles=10, Other=11 **(specify)**	

1.3 **Main material of the exterior walls:**<br>No walls=1, Cane/palm/trunks=2, Dirt=3, Bamboo with mud=4, Stone with mud=5, Plywood=6, Cardboard=7, Finished walls=8, Tin=9, Cement=10, Stone with lime/cement=11, Bricks=12, Wood planks/shingles=13, Other=14 **(specify)**	
1.4 How many rooms in this household are used for sleeping?	
1.5 Number of people living in the household:	
1.6 Number of people involved in income generating activities:	
1.7 **Water, Sanitation, Hygiene practices (WASH)**	
1.8 **What is the main source of drinking water for members of your household?**<br>Piped water=1, Tube well or borehole=2, Protected dug well=3, Unprotected dug well=4, Rainwater=5, Tanker truck=6, Cart with small tank=7, Surface water (river/dam/lake/pond/ stream/canal/irrigation channel)=8, Bottled water=9, Others=10 **(specify)**	
1.9 **Where is that water source located?**<br>In own dwelling= 1, In own yard/plot=2, Elsewhere=3	
1.10 **Do you do anything to the water to make it safer to drink?** Yes=1, No= 2, Unknown=9999	
1.11 ***If, Yes;* What do you usually do to make the water safer to drink**?<br>Boil=1, Add bleach/chlorine= 2, Strain through a cloth= 3, Use water filter (ceramic/sand/ composite/etc.)=4, Solar disinfection=5, let it stand and settle=6, other=7 **(specify),** Unknown=9999	
1.12 **What kind of toilet facility do members of your household usually use?**<br>Flush to piped sewer system=1, flush to septic tank=2, flush to pit latrine=3, flush to somewhere else=4, flush, don't know where=5, Ventilated improved pit latrine=6, pit latrine with slab=7, pit latrine without slab/open pit= 8, composting toilet=9, bucket toilet=10, hanging toilet/hanging=11, latrine=12, no facility/bush/field=13, others=14 **(specify)**	
1.13 **Do you share this toilet facility with other households?** Yes=1, No= 2	
1.14 ***If Yes,* how many households use this toilet facility?**	

D2. Asset score

2.1 **Does your household have:**<br>Electricity=1, Solar electricity=2, Radio=3, Television=4, Mobile telephone=5, Non-mobile telephone=6, Refrigerator=7, Almirah/wardrob=8, Electric fan=9, DVD/VCD player=10, Water pump=11, IPS/generator=12, Air conditioner=13, Computer/laptop=14, None of the mentioned=15	
2.2 **What type of fuel does your household mainly use for cooking?**<br>Electricity=1, Lpg =2, Natural gas=3, Biogas =4, Kerosene=5, Coal, lignite =6, Charcoal=7, Wood =8, Straw/shrubs/grass=9, Agricultural crop =10, Animal dung =11, None =12, Other=13 (specify)	
2.3 **Is the cooking usually done in the house, in a separate building, or outdoors?**<br>In the house=1, In a separate building =2, Outdoors=3, Other=4 (specify)	
2.4 **Does any member of this household own:**<br>Car/truck/microbus= 1, Autobike/tempo/CNG=2, Rickshaw/van=3, Bicycle=4, Motorcycle/scooter=5	
2.5 **Does this household own any livestock, herds, other farm animals, or poultry?** Yes=1, No=2	
2.6 If Yes, how many of the following animals does this household own?	
i. Buffaloes	
ii. Milk cows/bulls	
iii. Goat/sheep	
iv. Chickens/ducks	
v. Other farm animals	
2.7 **Does your household own any homestead?** IF 'NO' PROBE: Does your household own homestead in any other places? Yes=1, No=2	

2.8 **Does your household own any land (other than the homestead land)?** Yes=1, No=2	
2.9 If Yes, **how much land does your household own** (other than the homestead land)?	Amount<br>___________<br>Specify Unit<br>___________
2.10 **Does any member of this household have a bank account?** Yes=1, No=2	
2.11 **Monthly family income (BDT)**	
2.12 **Monthly family expenditure (BDT)**	
2.13 **Details about monthly family expenditure (BDT)**	
a. Total food expenditure in last month (BDT)	
b. Total health expenditure in last month (BDT)	
c. Total amount spent for treatment of child in last month (BDT)	

D3. Household food security

**Food items**<br><br>*[Do NOT count small quantities (less than 1 teaspoon)]*		**How many days was the food item eaten in previous 7 days?**<br>0 = Not eaten 1= 1 day 2= 2 days 3= 3 days, 4= 4 days 5= 5 days 6= 6 days 7= 7 days	**Sources of food (see codes below)**
3.1	**Rice/wheat and their products** *e.g. bread/ata/maida/muri/chira/khoi;* **Starchy roots** *e.g. potato/ sweet potato/kochu gati etc.*		
3.2	**Daal** *e.g. moshur/mung/kheshari/ chhola/koloi/ motor/soya;* **Beans** *e.g. sheem, cowpeas;* **Nuts**		
3.3	**Milk and milk products** *e.g. cow/goat milk (fresh)/dried milk; yogurt/cheese*		
3.4.1	**Flesh meat** e.g. *beef, lamb, goat, chicken, duck, other birds*		
3.4.2	**Organ meat** e.g. Liver, kidney, heart/ others		
3.4.3	**Fish (any type)**		
3.4.4	**Eggs**		
3.5.1	**Yellow/orange vegetables** *e.g. carrot/pumpkin/orange sweet potatoes/red spinach etc.*		
3.5.2	**Dark green leafy vegetables** *e.g. spinach, broccoli, amaranth and / or other dark green leaves, cassava leaves*		
3.5.3	**All other vegetables**		
3.6.1	**Yellow and orange colored fruits** *e.g. Mango, papaya, jackfruit, berry, apricot, peach. (NB: do not included oranges)*		
3.6.2	**All other fruits** e.g. Apple/Grapes/Orange/Banana/lichi etc.		
3.7.1	**Oil/butter/ghee**		
3.8	Sugar/honey/jam/crackers/candy/cookies/ *pastries, cakes and other sweet (sugary drinks)*		
3.9	**Condiments/Spices/Tea/salt/sauce**		

Food source codes: Purchase =1, Own production =2, Traded goods/services, barter =3 Borrowed = 4, Received as gift= 5, Food aid =6, Other (specify) =7

D4. **Household Food Insecurity Access Scale (HFIAS) Measurement Tool**

SI	Question	No=0, Yes=1	If 'Yes', How often did this happen?<br>**1=** Rarely (≤2 days in the last month)<br>**2=** Sometimes (3-10 days in last month)<br>**3=** Often (>10 days in last month)
4.1	In the past four weeks, did you worry that your household would not have enough food?		
4.2	In the past four weeks, were you or any household member not able to eat the kinds of foods you preferred because of a lack of resources?		
4.3	In the past four weeks, did you or any household member have to eat a limited variety of foods due to a lack of resources?		
4.4	In the past four weeks, did you or any household member have to eat some foods that you really did not want to eat because of a lack of resources to obtain other types of food?		
4.5	In the past four weeks, did you or any household member have to eat a smaller meal than you felt you needed because there was not enough food?		
4.6	In the past four weeks, did you or any other household member have to eat fewer meals in a day because there was not enough food?		
4.7	In the past four weeks, was there ever no food to eat of any kind in your household because of lack of resources to get food?		
4.8	In the past four weeks, did you or any household member go to sleep at night hungry because there was not enough food?		
4.9	In the past four weeks, did you or any household member go a whole day and night without eating anything because there was not enough food?		

E. Information about Primary Caregiver

E1. Social Capital (SAS-CAT)

Shortened and adapted Social Capital Assessment Tool for use in Bangladesh (SASCAT-B)

Structural social capital

Group membership

1a. In the last 12 months, have you been a member of the following types of groups in your area?

- Vocational training group
- Microcredit program
- Savings groups/community cooperative
- Sports club
- Political group
- Youth/student club
- Religious group
- Other: specify

1b. In the last 12 months, how would you describe your involvement in the groups in which you are a member?

- Received a loan or other form of financial support
- Participated in decision making
- Attended meetings
- Served as a leader of the group
- Attended trainings
- Other: specify

Social support

2a. Suppose you had something unfortunate happen to you, such as a father's sudden death. Who would help you in this situation?

Immediate family	Politicians
Relatives	Government officials/civil service
Neighbors	Person from NGO
Friends who are not neighbors	A group in which I am a member
Community leaders	A group in which I am not a member
Religious leaders	Other: specify

2b. Suppose you suffered an economic loss, such as job loss (URBAN) / crop failure (RURAL). In that situation, who do you think would assist you financially[a]?

2c. Suppose you are (FEMALE) / your wife is (MALE) preparing to give birth to your (FEMALE) / her (MALE) first child. Who do you think would provide you (FEMALE) / her (MALE) advice or assistance in this situation[a]?

Collective action

3. In the last 12 months, have you joined together with others in your area to address important issues?

    Yes

    No

4. In the last 12 months, have you talked with a local leader, chairman, or governmental organization about the development of your area?

    Yes

    No

Cognitive social capital

Trust

5a. Can your neighbors be trusted?

    Yes

    Sometimes

    No

5b. Can leaders in this area be trusted?

    Yes

    Sometimes

    No

6. Do you think that the majority of people in this area would try to take advantage of you if they got the chance?

    Yes

    Sometimes

    No

Social cohesion

7. Do the majority of people in this area generally have good relationships with each other?

    Yes

    Sometimes

    No

8. Do you feel that this area is yours?

    Yes

    Sometimes

    No

E2. Depression Anxiety and Stress scale (DASS 21)

Please read each statement and circle a number 0, 1, 2 or 3 which indicates how much the statement applied to you over the past week. There are no right or wrong answers. Do not spend too much time on any statement.

The rating scale is as follows:

0 Did not apply to me at all - NEVER

1 Applied to me to some degree, or some of the time - SOMETIMES

2 Applied to me to a considerable degree, or a good part of time - OFTEN

3 Applied to me very much, or most of the time - ALMOST ALWAYS

		N	S	O	AA
1	I found it hard to wind down	0	1	2	3
2	I was aware of dryness of my mouth	0	1	2	3
3	I couldn't seem to experience any positive feeling at all	0	1	2	3
4	I experienced breathing difficulty (eg, excessively rapid breathing, breathlessness in the absence of physical exertion)	0	1	2	3
5	I found it difficult to work up the initiative to do things	0	1	2	3
6	I tended to over-react to situations	0	1	2	3
7	I experienced trembling (eg, in the hands)	0	1	2	3
8	I felt that I was using a lot of nervous energy	0	1	2	3
9	I was worried about situations in which I might panic and make a fool of myself	0	1	2	3
10	I felt that I had nothing to look forward to	0	1	2	3
11	I found myself getting agitated	0	1	2	3
12	I found it difficult to relax	0	1	2	3
13	I felt down-hearted and blue	0	1	2	3
14	I was intolerant of anything that kept me from getting on with what I was doing	0	1	2	3
15	I felt I was close to panic	0	1	2	3
16	I was unable to become enthusiastic about anything	0	1	2	3
17	I felt I wasn't worth much as a person	0	1	2	3
18	I felt that I was rather touchy	0	1	2	3
19	I was aware of the action of my heart in the absence of physicalexertion (eg, sense of heart rate increase, heart missing a beat)	0	1	2	3
20	I felt scared without any good reason	0	1	2	3
21	I felt that life was meaningless	0	1	2	3

E3. Health related Quality of Life (SF 12)

SF-12 Health Survey

This survey asks for your views about your health. This information will help keep track of how you feel and how well you are able to do your usual activities. **Answer each question by choosing just one answer.** If you are unsure how to answer a question, please give the best answer you can.

1. In general, would you say your health is:

☐1 Excellent ☐2 Very good ☐3 Good ☐4 Fair ☐5 Poor

The following questions are about activities you might do during a typical day. Does your health now limit you in these activities? If so, how much?

	YES, limited a lot	YES, limited a little	NO, not limited at all
2. **Moderate activities** such as moving a table, pushing a vacuum cleaner, bowling, or playing golf.	☐1	☐2	☐3
3. Climbing **several** flights of stairs.	☐1	☐2	☐3

During the past 4 weeks, have you had any of the following problems with your work or other regular daily activities as a result of your physical health?

	YES	NO
4. **Accomplished less** than you would like.	☐1	☐2
5. Were limited in the **kind** of work or other activities.	☐1	☐2

During the past 4 weeks, have you had any of the following problems with your work or other regular daily activities as a result of any emotional problems (such as feeling depressed or anxious)?

	YES	NO
6. **Accomplished less** than you would like.	☐1	☐2
7. Did work or activities **less carefully than usual**.	☐1	☐2

8. During the past 4 weeks, how much did pain interfere with your normal work (including work outside the home and housework)?

☐1 Not at all ☐2 A little bit ☐3 Moderately ☐4 Quite a bit ☐5 Extremely

These questions are about how you have been feeling during the past 4 weeks.
For each question, please give the one answer that comes closest to the way you have been feeling.

How much of the time during the past 4 weeks…

	All of the time	Most of the time	A good bit of the time	Some of the time	A little of the time	None of the time
9. Have you felt calm & peaceful?	☐1	☐2	☐3	☐4	☐5	☐6
10. Did you have a lot of energy?	☐1	☐2	☐3	☐4	☐5	☐6
11. Have you felt down-hearted and blue?	☐1	☐2	☐3	☐4	☐5	☐6

12. During the past 4 weeks, how much of the time has your physical health or emotional problems interfered with your social activities (like visiting friends, relatives, etc.)?

☐1 All of the time ☐2 Most of the time ☐3 Some of the time ☐4 A little of the time ☐5 None of the time

F. Assessment for child with CP

F1. TNO-AZL Preschool quality of life (TAPQoL)

TAPQOL scales and items	First response	Second response [If 'first response is 'Occasionally/often']
**PHYSICAL FUNCTIONING**		
**Sleeping:** How did your child sleep...		
1. did your child sleep restlessly	3=never<br>2=occasionally<br>1=often	3=Fine<br>2=Not so good<br>1=Quite bad<br>0=Bad
2. was your child awake at night	3=never<br>2=occasionally<br>1=often	3=Fine<br>2=Not so good<br>1=Quite bad<br>0=Bad
3. did your child cry at night	3=never<br>2=occasionally<br>1=often	3=Fine<br>2=Not so good<br>1=Quite bad<br>0=Bad
4. did your child have difficulty sleeping through the night	3=never<br>2=occasionally<br>1=often	3=Fine<br>2=Not so good<br>1=Quite bad<br>0=Bad
**Appetite:** How did your child eat and drink...		
5. was your child's appetite poor	3=never<br>2=occasionally<br>1=often	3=Fine<br>2=Not so good<br>1=Quite bad<br>0=Bad
6. did your child have difficulty eating enough	3=never<br>2=occasionally<br>1=often	3=Fine<br>2=Not so good<br>1=Quite bad<br>0=Bad
7. did your child refuse to eat	3=never<br>2=occasionally<br>1=often	3=Fine<br>2=Not so good<br>1=Quite bad<br>0=Bad
**Lungs:** Has your child had.../Has your child been...		
8. Bronchitis	3=never<br>2=occasionally<br>1=often	3=Fine<br>2=Not so good<br>1=Quite bad<br>0=Bad
9. difficulty breathing or lung problems	3=never<br>2=occasionally<br>1=often	3=Fine<br>2=Not so good<br>1=Quite bad<br>0=Bad
10. short of breath	3=never<br>2=occasionally<br>1=often	3=Fine<br>2=Not so good<br>1=Quite bad<br>0=Bad
**Stomach:** Has your child had.../Has your child been...		

11. stomachache or abdominal pain	3=never<br>2=occasionally<br>1=often	3=Fine<br>2=Not so good<br>1=Quite bad<br>0=Bad
12. colic	3=never<br>2=occasionally<br>1=often	3=Fine<br>2=Not so good<br>1=Quite bad<br>0=Bad
13. nauseous	3=never<br>2=occasionally<br>1=often	3=Fine<br>2=Not so good<br>1=Quite bad<br>0=Bad
**Skin:** Has your child had...		
14. Eczema	3=never<br>2=occasionally<br>1=often	3=Fine<br>2=Not so good<br>1=Quite bad<br>0=Bad
15. Itchiness	3=never<br>2=occasionally<br>1=often	3=Fine<br>2=Not so good<br>1=Quite bad<br>0=Bad
16. dry child	3=never<br>2=occasionally<br>1=often	3=Fine<br>2=Not so good<br>1=Quite bad<br>0=Bad
**Motor functioning:** Did your child have...		
1. difficulty with walking	3=never<br>2=occasionally<br>1=often	3=Fine<br>2=Not so good<br>1=Quite bad<br>0=Bad
2. difficulty with running	3=never<br>2=occasionally<br>1=often	3=Fine<br>2=Not so good<br>1=Quite bad<br>0=Bad
3. difficulty with walking upstairs without help	3=never<br>2=occasionally<br>1=often	3=Fine<br>2=Not so good<br>1=Quite bad<br>0=Bad
4. difficulty with balance	3=never<br>2=occasionally<br>1=often	3=Fine<br>2=Not so good<br>1=Quite bad<br>0=Bad
**SOCIAL FUNCTIONING**		
**Social functioning:** How was your child's behaviour with older children		
1. my child was able to play happily with other children	3=never<br>2=occasionally<br>1=often	Not Applicable
2. my child was at ease with other children	3=never<br>2=occasionally<br>1=often	Not Applicable
3. my child was confident with other children	3=never<br>2=occasionally<br>1=often	Not Applicable

**Problem behavior:** Your child's behaviour...		
4. my child was short-tempered	3=never<br>2=occasionally<br>1=often	Not Applicable
5. my child was aggressive	3=never<br>2=occasionally<br>1=often	Not Applicable
6. my child was irritable	3=never<br>2=occasionally<br>1=often	Not Applicable
7. my child was angry	3=never<br>2=occasionally<br>1=often	Not Applicable
8. my child was restless or impatient with me	3=never<br>2=occasionally<br>1=often	Not Applicable
9. my child was defiant/awkward with me	3=never<br>2=occasionally<br>1=often	Not Applicable
10. I could not manage my child	3=never<br>2=occasionally<br>1=often	Not Applicable
**COGNITIVE FUNCTIONING**		
**Communication:** Did your child have..		
11. difficulty in understanding what others said	3=never<br>2=occasionally<br>1=often	3=Fine<br>2=Not so good<br>1=Quite bad<br>0=Bad
12. difficulty in talking clearly	3=never<br>2=occasionally<br>1=often	3=Fine<br>2=Not so good<br>1=Quite bad<br>0=Bad
13. difficulty in saying what he/she meant	3=never<br>2=occasionally<br>1=often	3=Fine<br>2=Not so good<br>1=Quite bad<br>0=Bad
14. difficulty in making it clear what he/she wanted	3=never<br>2=occasionally<br>1=often	3=Fine<br>2=Not so good<br>1=Quite bad<br>0=Bad
**EMOTIONAL FUNCTIONING**		
**Anxiety:** How was your child...		
15. **frightened**	3=never<br>2=occasionally<br>1=often	Not Applicable
16. **tense**	3=never<br>2=occasionally<br>1=often	Not Applicable
17. **anxious**	3=never<br>2=occasionally<br>1=often	Not Applicable

F2. GROSS MOTOR FUNCTION MEASURE (GMFM-66)

Check (3) **the appropriate score:** if an item is not tested (NT), circle the item number on the right column

	Item	A: LYING & ROLLING	SCORE				NT
	1.	SUP, HEAD IN MIDLINE: TURNS HEAD WITH EXTREMITIES SYMMETRICAL	0☐	1☐	2☐	3☐	1.
*	2.	SUP: BRINGS HANDS TO MIDLINE, FINGERS ONE WITH THE OTHER	0☐	1☐	2☐	3☐	2.
	3.	SUP: LIFTS HEAD 45°	0☐	1☐	2☐	3☐	3.
	4.	SUP: FLEXES R HIP & KNEE THROUGH FULL RANGE	0☐	1☐	2☐	3☐	4.
	5.	SUP: FLEXES L HIP & KNEE THROUGH FULL RANGE	0☐	1☐	2☐	3☐	5.
*	6.	SUP: REACHES OUT WITH R ARM, HAND CROSSES MIDLINE TOWARD TOY	0☐	1☐	2☐	3☐	6.
*	7.	SUP: REACHES OUT WITH L ARM, HAND CROSSES MIDLINE TOWARD TOY	0☐	1☐	2☐	3☐	7.
	8.	SUP: ROLLS TO PR OVER R SIDE	0☐	1☐	2☐	3☐	8.
	9.	SUP: ROLLS TO PR OVER L SIDE	0☐	1☐	2☐	3☐	9.
*	10.	PR: LIFTS HEAD UPRIGHT	0☐	1☐	2☐	3☐	10.
	11.	PR ON FOREARMS: LIFTS HEAD UPRIGHT, ELBOWS EXT., CHEST RAISED	0☐	1☐	2☐	3☐	11.
	12.	PR ON FOREARMS: WEIGHT ON R FOREARM, FULLY EXTENDS OPPOSITE ARM FORWARD	0☐	1☐	2☐	3☐	12.
	13.	PR ON FOREARMS: WEIGHT ON L FOREARM, FULLY EXTENDS OPPOSITE ARM FORWARD	0☐	1☐	2☐	3☐	13.
	14.	PR: ROLLS TO SUP OVER R SIDE	0☐	1☐	2☐	3☐	14.
	15.	PR: ROLLS TO SUP OVER L SIDE	0☐	1☐	2☐	3☐	15.
	16.	PR: PIVOTS TO R 90° USING EXTREMITIES	0☐	1☐	2☐	3☐	16.
	17.	PR: PIVOTS TO L 90° USING EXTREMITIES	0☐	1☐	2☐	3☐	17.
		**TOTAL DIMENSION A**					

	Item	B: SITTING	SCORE				NT
*	18.	SUP, HANDS GRASPED BY EXAMINER: PULLS SELF TO SITTING WITH HEAD CONTROL	0☐	1☐	2☐	3☐	18.
	19.	SUP: ROLLS TO R SIDE, ATTAINS SITTING	0☐	1☐	2☐	3☐	19.
	20.	SUP: ROLLS TO L SIDE, ATTAINS SITTING	0☐	1☐	2☐	3☐	20.
*	21.	SIT ON MAT, SUPPORTED AT THORAX BY THERAPIST: LIFTS HEAD UPRIGHT, MAINTAINS 3 SECONDS	0☐	1☐	2☐	3☐	21.
*	22.	SIT ON MAT, SUPPORTED AT THORAX BY THERAPIST: LIFTS HEAD MIDLINE, MAINTAINS 10 SECONDS	0☐	1☐	2☐	3☐	22.
*	23.	SIT ON MAT, ARM(S) PROPPING: MAINTAINS, 5 SECONDS	0☐	1☐	2☐	3☐	23.
*	24.	SIT ON MAT: MAINTAIN, ARMS FREE, 3 SECONDS	0☐	1☐	2☐	3☐	24.
*	25.	SIT ON MAT WITH SMALL TOY IN FRONT: LEANS FORWARD, TOUCHESTOY, RE-ERECTS WITHOUT ARM PROPPING	0☐	1☐	2☐	3☐	25.
*	26.	SIT ON MAT: TOUCHES TOY PLACED 45° BEHIND CHILD'S R SIDE, RETURNS TO START	0☐	1☐	2☐	3☐	26.
*	27.	SIT ON MAT: TOUCHES TOY PLACED 45° BEHIND CHILD'S L SIDE, RETURNS TO START	0☐	1☐	2☐	3☐	27.
	28.	R SIDE SIT: MAINTAINS, ARMS FREE, 5 SECONDS	0☐	1☐	2☐	3☐	28.
	29.	L SIDE SIT: MAINTAINS, ARMS FREE, 5 SECONDS	0☐	1☐	2☐	3☐	29.
*	30.	SIT ON MAT: LOWERS TO PR WITH CONTROL	0☐	1☐	2☐	3☐	30.
*	31.	SIT ON MAT WITH FEET IN FRONT: ATTAINS 4 POINT OVER R SIDE	0☐	1☐	2☐	3☐	31.
*	32.	SIT ON MAT WITH FEET IN FRONT: ATTAINS 4 POINT OVER L SIDE	0☐	1☐	2☐	3☐	32.
	33.	SIT ON MAT: PIVOTS 90°, WITHOUT ARMS ASSISTING	0☐	1☐	2☐	3☐	33.
*	34.	SIT ON BENCH: MAINTAINS, ARMS AND FEET FREE, 10 SECONDS	0☐	1☐	2☐	3☐	34.
*	35.	STD: ATTAINS SIT ON SMALL BENCH	0☐	1☐	2☐	3☐	35.
*	36.	ON THE FLOOR: ATTAINS SIT ON SMALL BENCH	0☐	1☐	2☐	3☐	36.
*	37.	ON THE FLOOR: ATTAINS SIT ON LARGE BENCH	0☐	1☐	2☐	3☐	37.
		**TOTAL DIMENSION B**					

	Item	C: CRAWLING & KNEELING	SCORE				NT
	38.	PR: CREEPS FORWARD 1.8m (6')	0☐	1☐	2☐	3☐	38.
*	39.	4 POINT: MAINTAINS, WEIGHT ON HANDS AND KNEES, 10 SECONDS	0☐	1☐	2☐	3☐	39.
*	40.	4 POINT: ATTAINS SIT ARMS FREE	0☐	1☐	2☐	3☐	40.
*	41.	PR: ATTAINS 4 POINT, WEIGHT ON HANDS AND KNEES	0☐	1☐	2☐	3☐	41.
*	42.	4 POINT: REACHES FORWARD WITH R ARM, HAND ABOVE SHOULDER LEVEL	0☐	1☐	2☐	3☐	42.
*	43.	4 POINT: REACHES FORWARD WITH L ARM, HAND ABOVE SHOULDER LEVEL	0☐	1☐	2☐	3☐	43.
*	44.	4 POINT: CRAWLS OR HITCHES FORWARD 1.8m(6')	0☐	1☐	2☐	3☐	44.
*	45.	4 POINT: CRAWLS RECIPROCALLY FORWARD1.8m (6')	0☐	1☐	2☐	3☐	45.
*	46.	4 POINT: CRAWLS UP 4 STEPS ON HANDS AND KNEES/FEET	0☐	1☐	2☐	3☐	46.
	47.	4 POINT: CRAWLS BACKWARDS DOWN 4 STEPS ON HANDS AND KNEES/FEET	0☐	1☐	2☐	3☐	47.
*	48.	SIT ON MAT: ATTAINS HIGH KN USING ARMS, MAINTAINS, ARMS FREE, 10 SECONDS	0☐	1☐	2☐	3☐	48.
	49.	HIGH KN: ATTAINS HALF KN ON R KNEE USING ARMS, MAINTAINS, ARMS FREE, 10 SECONDS	0☐	1☐	2☐	3☐	49.
	50.	HIGH KN: ATTAINS HALF KN ON L KNEE USING ARMS, MAINTAINS, ARMS FREE, 10 SECONDS	0☐	1☐	2☐	3☐	50.
*	51.	HIGH KN: KN WALKS FORWARD 10 STEPS, ARMS FREE	0☐	1☐	2☐	3☐	51.
		**TOTAL DIMENSION C**					

	Item	D: STANDING	SCORE				NT
*	52.	ON THE FLOOR: PULLS TO STD AT LARGE BENCH	0☐	1☐	2☐	3☐	52.
*	53.	STD: MAINTAINS, ARMS FREE, 3 SECONDS	0☐	1☐	2☐	3☐	53.
*	54.	STD: HOLDING ON TO LARGE BENCH WITH ONE HAND, LIFTS R FOOT, 3 SECONDS	0☐	1☐	2☐	3☐	54.
*	55.	STD: HOLDING ON TO LARGE BENCH WITH ONE HAND, LIFTS L FOOT, 3 SECONDS	0☐	1☐	2☐	3☐	55.
*	56.	STD: MAINTAINS, ARMS FREE, 20 SECONDS	0☐	1☐	2☐	3☐	56.
*	57.	STD: LIFTS L FOOT, ARMS FREE, 10 SECONDS	0☐	1☐	2☐	3☐	57.
*	58.	STD: LIFTS R FOOT, ARMS FREE, 10 SECONDS	0☐	1☐	2☐	3☐	58.
*	59.	SIT ON SMALL BENCH: ATTAINS STD WITHOUT USING ARMS	0☐	1☐	2☐	3☐	59.
*	60.	HIGH KN: ATTAINS STD THROUGH HALF KN ON R KNEE, WITHOUT USING ARMS	0☐	1☐	2☐	3☐	60.
*	61.	HIGH KN: ATTAINS STD THROUGH HALF KN ON L KNEE, WITHOUT USING ARMS	0☐	1☐	2☐	3☐	61.
*	62.	STD: LOWERS TO SIT ON FLOOR WITH CONTROL, ARMS FREE	0☐	1☐	2☐	3☐	62.
*	63.	STD: ATTAINS SQUAT, ARMS FREE	0☐	1☐	2☐	3☐	63.
*	64.	STD: PICKS UP OBJECT FROM FLOOR, ARMS FREE, RETURNS TO STAND	0☐	1☐	2☐	3☐	64.
		**TOTAL DIMENSION D**					

	Item	E: WALKING, RUNNING & JUMPING	SCORE				NT
*	65.	STD, 2 HANDS ON LARGE BENCH: CRUISES 5 STEPS TO R	0☐	1☐	2☐	3☐	65.
*	66.	STD, 2 HANDS ON LARGE BENCH: CRUISES 5 STEPS TO L	0☐	1☐	2☐	3☐	66.
*	67.	STD, 2 HANDS HELD: WALKS FORWARD 10 STEPS	0☐	1☐	2☐	3☐	67.
*	68.	STD, 1 HAND HELD: WALKS FORWARD 10 STEPS	0☐	1☐	2☐	3☐	68.
*	69.	STD: WALKS FORWARD 10 STEPS	0☐	1☐	2☐	3☐	69.
*	70.	STD: WALKS FORWARD 10 STEPS, STOPS, TURNS 180°, RETURNS	0☐	1☐	2☐	3☐	70.
*	71.	STD: WALKS BACKWARD 10 STEPS	0☐	1☐	2☐	3☐	71.
*	72.	STD: WALKS FORWARD 10 STEPS, CARRYING A LARGE OBJECT WITH 2 HANDS	0☐	1☐	2☐	3☐	72.
*	73.	STD: WALKS FORWARD 10 CONSECUTIVE STEPS BETWEEN PARALLEL LINES 20cm (8")APART	0☐	1☐	2☐	3☐	73.
*	74.	STD: WALKS FORWARD 10 CONSECUTIVE STEPS ON A STRAIGHT LINE 2cm (3/4") WIDE	0☐	1☐	2☐	3☐	74.
*	75.	STD: STEPS OVER STICK AT KNEE LEVEL, R FOOT LEADING	0☐	1☐	2☐	3☐	75.

*	76.	STD: STEPS OVER STICK AT KNEE LEVEL, L FOOT LEADING	0☐	1☐	2☐	3☐	76.
*	77.	STD: RUNS 4.5m (15'), STOPS & RETURNS	0☐	1☐	2☐	3☐	77.
*	78.	STD: KICKS BALL WITH R FOOT	0☐	1☐	2☐	3☐	78.
*	79.	STD: KICKS BALL WITH L FOOT	0☐	1☐	2☐	3☐	79.
*	80.	STD: JUMPS 30cm (12") HIGH, BOTH FEET SIMULTANEOUSLY	0☐	1☐	2☐	3☐	80.
*	81.	STD: JUMPS FORWARD 30 cm (12"), BOTH FEET SIMULTANEOUSLY	0☐	1☐	2☐	3☐	81.
*	82.	STD ON R FOOT: HOPS ON R FOOT 10 TIMES WITHIN A 60cm (24") CIRCLE	0☐	1☐	2☐	3☐	82.
*	83.	STD ON L FOOT: HOPS ON L FOOT 10 TIMES WITHIN A 60cm (24") CIRCLE	0☐	1☐	2☐	3☐	83.
*	84.	STD, HOLDING 1 RAIL: WALKS UP 4 STEPS, HOLDING 1 RAIL, ALTERNATING FEET	0☐	1☐	2☐	3☐	84.
*	85.	STD, HOLDING 1 RAIL: WALKS DOWN 4 STEPS, HOLDING 1 RAIL, ALTERNATING FEET	0☐	1☐	2☐	3☐	85.
*	86.	STD: WALKS UP 4 STEPS, ALTERNATING FEET	0☐	1☐	2☐	3☐	86.
*	87.	STD: WALKS DOWN 4 STEPS, ALTERNATING FEET	0☐	1☐	2☐	3☐	87.
*	88.	STD ON 15cm (6") STEP: JUMPS OFF, BOTH FEET SIMULTANEOUSLY	0☐	1☐	2☐	3☐	88.

TOTAL DIMENSION E ☐

F3. GMFCS level of the child

☐	Level I
☐	Level II
☐	Level III
☐	Level IV
☐	Level V

F4. Communication Functional Classification System (CFCS)

The following **methods of communication** are used by this individual: *(Please check **all** that apply)*

☐	Speech
☐	Sounds (such as an "aaaah" to get a partner's attention)
☐	Eye gaze, facial expressions, gesturing, and/or pointing (e.g., with a body part, stick, laser)
☐	Manual signs
☐	Communication book, boards, and/or pictures
☐	Voice output device or a speech-generating device
☐	Other

G. CFCS level identification chart

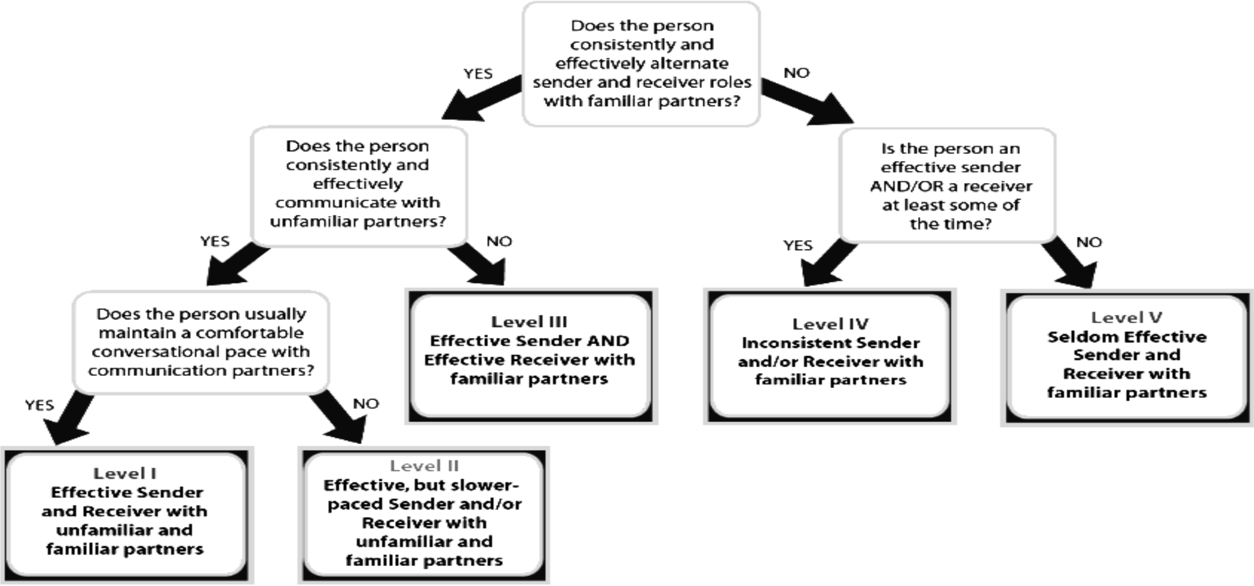

H. Anthropometric measurement of the child

1. Weight (kg):		2. MUAC (cm):	
3.1 Height (cm):		3.2 Knee height (cm):	
[for children with deformities, estimate height using the knee height equation, Height= (2.69 X knee height) + 24.2]			
4. Head circumference (cm):		5.1 Skinfold-thickness (bicep) (mm):	
5.2 Skinfold-thickness (tricep) (mm):		5.3 Skinfold-thickness (subscapular) (mm):	

I. Child's Rehabilitation and educational status

1. Has the child ever received any rehabilitation service or other related support	☐ Yes ☐ No
2. What type of support was received? ☐Assistive device ☐Surgery ☐Therapy exercises ☐ Advice ☐ Other, Specify ____________________	
3. What was the type of location for accessing these rehabilitation services? ☐Home based ☐NGO centre ☐ Hospital ☐ Private clinic	
If 1 is NO, Reason why child NEVER received rehabilitation? ☐ Not aware ☐ No money ☐ Transport problem ☐ Others, please specify	
4. Is the child currently attending any special school? ☐ Yes=1, ☐ No=0	

**Sections**	**Data collected by (signature, date)**
A. Basic information	
B. Contact details of primary caregiver	
C. Birth parent details	
D1. Housing characteristics	
D2. Asset score	
D3. Household food security	
D4. Household Food Insecurity Access Scale (HFIAS) Measurement Tool	
E1. Social Capital (SAS-CAT)	

E2. Depression Anxiety and Stress scale (DASS 21)	
E3. Health related Quality of Life (SF 12)	
F1. TNO-AZL Preschool quality of life (TAPQoL)	
F2. Gross Motor Function Measure (GMFM-66)	
F3. GMFCS level of the child	
F4. Communication Functional Classification System (CFCS)	
G. CFCS level identification chart	
H. Anthropometric measurement of the child	
I. Child's Rehabilitation and educational status	

Ethical Approval Application
To Asian Institute of Disability and Development (AIDD) Ethics Committee

(Electronic Format Only)

Date:	02 /05 /2019

This application must be typewritten. If the space available is not sufficient, attach details on a separate sheet. If this project includes any information of a commercial or patentable nature, this information should be sent separately and marked "Confidential". Please submit in electronic format to ***disabilityasia@gmail.com***

You can submit the approved participant information sheets and consent forms. Please also submit the approval letter in electronic format to ***disabilityasia@gmail.com***

**Project Title:**	**Supporting Ultra-Poor People with Rehabilitation and Therapy - a randomized controlled trial among families of children with Cerebral Palsy in rural Bangladesh (SUPPORT CP trial)**
**Names(s), Titles(s), Qualifications, Dept/Locations and Contact Details**	
**Principal Investigator:**	Professor Gulam Khandaker, Central Queensland Public Health Unit, Central Queensland Hospital and Health Service, Rockhampton, Queensland, Australia.<br>Email: gulam.khandaker@health.nsw.gov.au
**Associates and Co-Investigator:**	Professor Mohammad Muhit, CSF Global, Dhaka, Bangladesh<br><br>Ms Israt Jahan, CSF Global, Dhaka, Bangladesh<br><br>Dr Manik Chandra Das, CSF Global, Dhaka, Bangladesh<br><br>Mr Mahmudul Hassan Al Imam, CSF Global, Dhaka, Bangladesh<br><br>Mx Rosalie Power, Discipline of Child and Adolescent Health, Sydney Medical School, The University of Sydney, Sydney, Australia<br><br>Dr Arifuzzaman Khan, School of Public Health, The University of Queensland, Brisbane, Australia<br><br>Associate Professor Delwar Akbar, School of Business and Law, Central Queensland University, Rockhampton, Queensland, Australia<br><br>Professor Nadia Badawi, Discipline of Child and Adolescent Health, Sydney Medical School, The University of Sydney, Sydney, Australia
**Proposed Date of project commencement:**	Upon HREC approval
**Proposed Duration of Project:**	24 months
**Give succinct but comprehensive aims, hypotheses and potential significance of the project, or of its other purposes, noting also the expected benefits**	The aim of this study is to test the effectiveness of an "Integrated Microfinance/livelihood and community-based rehabilitation" (IMCBR) program targeted to children with cerebral palsy (CP) and their parents from ultra-poor families in rural Bangladesh. The program aims to improve the health-related quality of life (HRQoL), motor function, communication and nutritional status of children with CP; mental health, HRQoL and social capital of their parents; and socio-economic status and food security of their families.<br><br>Our specific objectives are;<br>1. To conduct a randomized controlled trial (RCT) with three parallel arms comparing (a) IMCBR, (b) community-based rehabilitation (CBR) alone, and (c) care-as-usual (i.e. no intervention).

	2. To measure the effectiveness of IMCBR in improving the HRQoL, motor function, communication, and nutritional status of children with CP from ultra-poor families living in rural Bangladesh.<br>3. To measure the effectiveness of IMCBR in improving mental health, HRQoL, and social capital of parents of children with CP living in rural Bangladesh.<br>4. To measure the effectiveness of IMCBR in improving the socio-economic status of ultra-poor families of children with CP living in rural Bangladesh.<br><br>**Hypothesis**<br>We hypothesize that compared to care-as-usual and CBR alone, the IMCBR program will be more effective in improving HRQoL, motor function, communication, and nutritional outcomes of children with CP from ultra-poor families; and the mental health, HRQoL, and social capital of their primary caregivers; and overall improvement in socio-economic status of the ultra-poor families of children with CP in rural Bangladesh.<br><br>**Significance**<br>To the best of our knowledge, this will be the first RCT of an integrated microfinance/livelihood and CBR program for children with CP in LMIC settings. Evidence from the study could transform approaches to improving the wellbeing of children with CP and their families living in extreme poverty. The study has been informed by the findings of a population-based surveillance (i.e. Bangladesh CP Register) and CBR in the local areas, indicating the need for interventions to focus on both the health and economic improvement. This study will be able to compare the effectiveness of CBR with a new integrated intervention as well as comparing both with standard care practiced in the locality. We propose a six months follow-up after completion of the intervention to test the longer-term impact of the intervention. These data will be scientifically valuable for large scale sustainable program implementation.<br><br>On the other hand, people with disabilities and their families are often excluded from social and economic activities in low- and middle-income countries (LMICs). About 97% of the families of children with CP in rural Bangladesh live in extreme poverty [1]; only 31% of ultra-poor families of people with disabilities receive government benefits (in form of social protection) [2]. If the proposed integrated program is proved to be scientifically effective in improving the wellbeing of children with CP and their caregivers, health, and economic outcomes of the families, the implementing partner plans to scale up the program using existing connections with NGOs and microfinance organizations in Bangladesh, and in other LMICs where CSF Global is research active (e.g. Nepal, Indonesia and Ghana).
**Give a succinct but comprehensive statement of the scientific background to the project and project plan**	Bangladesh CP Register research findings confirm that poverty is a key contributor to late diagnosis and limited access to early intervention and rehabilitation for children with CP in rural Bangladesh [1]. 97% families of children with CP in the country live below the poverty line. Therefore, in LMICs, efforts to improve outcomes for children with CP (including quality of life, motor, cognitive and nutritional attainments) should also include measures to improve family economic/social capital. We propose a randomized controlled trial to evaluate the effectiveness of an integrated microfinance/livelihood and community-based rehabilitation (IMCBR) program for ultra-poor families of children with CP in rural Bangladesh. We hypothesize that IMCBR will facilitate improved access to capital leading to better income and thus increase the family's investment in physical health overall. Moreover, CBR will provide an opportunity for sharing ideas, information, and developing important non-cognitive skills, such as self-confidence of primary caregivers.
**Briefly describe all methodology to be used with participants**	This will be a cluster RCT comparing three arms: (a) IMCBR; (b) CBR alone; and (c) care-as-usual (i.e. no intervention).<br><br>Seven clusters will be recruited within each arm. Each cluster will consist of 10 child-caregiver dyads totaling 21 clusters with 210 dyads.<br><br>Parents recruited in the IMCBR arm will take part in a microfinance/livelihood program and CP Parent Training Module (PTM), their child with CP will take part in a Goal Directed Training (GDT) program on a weekly basis. The CBR arm includes the same GDT and PTM

	interventions excluding the microfinance/livelihood program. The programs will be facilitated by specially trained Community Rehabilitation Officers (CROs). Whereas, the care-as-usual arm will be provided with information about early intervention and rehabilitation.<br><br>The duration of the interventions will be 12 months and outcomes will be measured at baseline, and at 6, 12, and 18 months using a standard pre-tested questionnaire. The assessors will be blinded to group allocation.<br><br>Data management and analysis will be conducted using STATA.
**Give a statement of the possible dangers or ill effects of these procedures and the precautions to be taken to prevent or minimize them**	It is extremely unlikely that GDT as part of CBR will result in any adverse outcomes. However, to minimize any potential risks from rehabilitative services the CROs will be trained thoroughly on basic therapeutic skills. In addition to that, they will also be taught how to assess adverse effects and when to stop providing rehabilitation services. Furthermore, one experienced Research Physiotherapist will be responsible for supervising CROs and monitor their service delivery. Each participant will also be assessed thoroughly by a trained clinician and physiotherapist at the first stage of the study and if there is any contraindication for active therapy, the participant will not be recruited into the study.
**Give a statement on the demands, inconvenience or discomfort to the participants**	We will use adequate safeguards (as described above) to minimize any associated physical risks. There will be no potential risk (related to privacy) to the participants from this study. Assessment and interview for the study will be conducted in communities close to the study participants. It is possible that the attendance to the weekly meeting may result in a loss of work time. Participation in the study may require 180 minutes maximum per week and participants will be registered in the study only if they agree to commit this time voluntarily. It is expected that parents will participate in the weekly meetings realizing that this will be beneficial for their child. However, no monetary benefits will be given to compensate for the given time. The consent form will contain both of these information. Families will be provided with information about the study. The health professionals will explain in detail the purpose of the study. If they are illiterate, the information sheet will be read out to the family members/caregivers in the local language (Bengali) and written consent will be obtained from the participants or caregivers.
**Give the number, type and age range of all the participants, including controls**	Participants will be considered eligible for participation based on the following criteria:<br>1. Children with CP aged ≤5 years, classified as from an ultra-poor family (i.e. per day per capita income <1.90 USD; [3]) and registered in the BCPR. The BCPR registers children with CP following the case definition of CP used by Surveillance of CP in Europe (SCPE) and the Australian CP Register (ACPR) [1].<br>2. Primary caregiver (e.g. parent, sibling, grandparent of the child with CP)<br>3. Primary caregiver has the capacity to give informed consent and is willing to take part in the study including microfinance/livelihood arm along with their child with CP.<br><br>**Sample size calculation**<br>The sample size for this cluster RCT has been computed based on methods described in Donner et al. [4]. We will recruit seven clusters in each arm, and each cluster will consist of 10 CP children with CP- primary caregivers dyads totaling 21 clusters of 210 dyads. Based on our pilot data and existing literature we predict 35% improvement of HRQoL in the IMCBR group, 20% improvement in the CBR alone group, and 5% improvement in the care-as-usual group. With a sample size of 210 dyads, this study will have 80% statistical power to detect these effects (two-sided α-value=0.05) [5]. Power calculation takes into account up to 20% sample attrition by the end of the trial. A homogeneous study population will allow us to balance randomization considering intra-cluster correlation of 0.5 and coefficient of variation in cluster size of 0.5.
**Sources and means of recruitment**	The study will utilize the BCPR as a sampling frame for participant recruitment. The BCPR is an ongoing surveillance of children with CP commenced in 2015 [1], and currently being operated in four districts of Bangladesh. Between 2015 and 2019, 1125 children with CP have been registered into the BCPR from Shahjadpur (i.e. the study site). As part of this RCT, dyads of children with CP and their primary caregivers who meet the inclusion criteria will be recruited and assigned to different arms of the study.

**Will any special relationship exist between the recruiter and the participants?**	No
**Criteria for exclusion**	Participants will be considered ineligible for participation based on the following criteria:<br>1. Currently in receipt of microfinance/livelihood support from another source.<br>2. Currently participating in any other clinical trial or intervention program.
**Details of any proposed payment to participants**	No payment will be made to the study participants
**Where will the procedures involving participants be undertaken?**	The study will be implemented in Shahjadpur sub-district (~324.15 sq. km) of Sirajganj district located in the northern part of Bangladesh. The study site is comprised of ~70,998 households with a total population of ~561,076 (child population aged 0-18 years ~226,114), and 12,117 live births per annum [1]. The study site constitutes a complex socio-demographic locale including urban, rural, and hard-to-reach areas and represents the overall socio-demographic and economic characteristics of rural areas in Bangladesh [6, 7].
**How will risk factors be minimized?**	An independent Data Safety and Monitoring Board (DSMB) will be formed for this trial. The members of DSMB will meet monthly to monitor the safety of trial participants and the quality of trial data. The Chair of the DSMB will report to Chief Investigator regarding issues related to data safety, quality of intervention and serious adverse event.<br><br>A serious adverse event will be defined as any event that results in injury, requires inpatient hospitalization or prolongation of existing hospitalization, or death or results in a persistent or significant disability or incapacity.<br><br>The DSMB will conduct a blinded interim analysis of effectiveness and safety endpoints once 210 participants have completed the trial. The DSMB may recommend continuing the trial, early termination of the trial, or modification of the trial. A recommendation to terminate the trial early will be made if there is clear evidence of a clinically harmful effect. The trial will not be stopped early on the grounds of futility.
**How will information be handled to safeguard confidentiality both during and after completion of the research project?**	Data will be collected using paper-based forms. Research data will be anonymized and stored securely and separately for participant identifiable information. This will include monitoring secure data transfer from field to central office, data entry and quality control of completed forms, querying of missing or invalid data, and archiving of physical forms. Data will be collected using validated questionnaires. Data will be entered into PCs using Microsoft Access or SQL Server as the relational database engine. Any error identified during data entry or in data cleaning will be logged for field supervisor assessment and will be resolved after proper field verification. The physical data will be stored for 7 years in a locked cabinet at head office of CSF Global based in Bangladesh as per national and international guidelines.
**If the project involves use of medication/drugs/ procedure, give details:**	**Intervention**<br>**Arm A- Integrated Microfinance/livelihood and Community-Based Rehabilitation (IMCBR)**<br>Participants randomized to IMCBR arm will be supported to create microfinance/livelihood groups (10 participant-pairs per group). The groups will be formed voluntarily along geographical boundaries to facilitate participation, retention, and meeting logistics. Each cluster will meet weekly to discuss microfinance/livelihood activities (e.g. weekly credit collection and troubleshooting) (90 minutes) and for CBR with children with CP comprising early intervention and primary caregiver's education (90 minutes).<br><br>*A.1 Microfinance/livelihood program details*<br>Group meetings will be organized with members of each cluster to discuss (i) details of the program, (ii) potential benefits and challenges of participation in the program, and (iii) motivations for participation. Participants of each cluster can then apply for a loan/livelihood support; a minimum 10% deposit of the requested loan/livelihood support amount in the form

	of savings is required and is admissible immediately after cluster formation. Once the application is completed, loan approval and disbursement of the loan will occur approximately within one week.<br>Amount, return cycle of loan and investment areas: Each of the ultra-poor families will receive a loan/livelihood support amounting/equivalent to ~100-300AUD at 12% flat interest rate. The return cycle will be one year with a weekly repayment schedule. Common investment areas for the ultra-poor loan will be for goat or cattle rearing, seeds for agriculture, home-based weaving, and handicraft business [8].<br><br>*A.2 CBR*<br>There will be two major components of the CBR program, which will occur during cluster meetings following the microfinance/livelihood portion.<br><br>a. Goal Directed Training (GDT): Community-based GDT focused on motor learning will be conducted with children with CP and their primary caregivers. GDT is an activity-based approach to therapy where meaningful, client-selected (i.e. caregivers of children with CP) goals are used to provide opportunities for problem-solving and to indirectly drive the movements required to successfully meet task demands [9]. Evidence from a meta-analysis shows that GDT based interventions are highly effective and should be the gold standard treatment for CP [10]. In this study, GDT will be delivered by child's primary caregiver (participating parent).<br><br>*b. Parents Training Module (PTM):* Primary caregivers will participate in PTM to learn basic therapeutically correct skills for the day-to-day care and support of their child with CP embedded in the principles of GDT. This study will follow the PTM 'Getting to know cerebral palsy' which includes 10 modules and covers topics; introduction to CP, evaluating your child, positioning and carrying, communication, everyday activities, feeding your child, play, disability in your local community, running your own parent support group, and assistive devices and resources [11].<br><br>Specially trained Community Rehabilitation Officers (CRO's) will facilitate each of the cluster meetings (both microfinance/livelihood and CBR activities). The CROs will facilitate microfinance/livelihood discussions and lead the GDT and PTM sessions with the aim to upskill primary caregivers so that they can continue to deliver GDT independently at home. Prior to implementation of the RCT, CROs will take part in a 5-day training by Research Physiotherapist. The CRO training will cover the following areas; (i) socio-cultural considerations in working with primary caregivers of children with CP, (ii) introduction to microfinance/livelihood support program management, (iii) developmental milestones and development in children with CP, (iv) therapeutic principles, (v) GDT, (vi) activity focused therapies, (vii) basic speech development strategies, (viii) contraindications of therapies, (ix) research ethics, and (x) child rights.<br><br>**Arm B- CBR alone**<br>Participants from clusters randomized to this arm will attend a weekly rehabilitation session at a local focal point (preferably one of the group members home). Each session will last for 90 minutes and will be identical to the CBR component of IMCBR (discussed above); however, the microfinance/livelihood component will not be provided.<br><br>**Arm C- Care-as-usual (i.e. no intervention)**<br>This group will not receive any active intervention. Once children with CP are identified and randomized into clusters, the 'care-as-usual' participants will be provided with basic education on early intervention and rehabilitation and will be encouraged to access healthcare via usual routes, which typically include treatment in government hospitals.

**Has this project been submitted to any other Ethics Committee?**	☐ Yes (✓) No
**If yes; name of committee**<br>*(please attach a copy of approval)*	

**Approval granted?**	☐ Yes ☐ No
**What do you think are the ethical issues raised by the proposed project considering your previous answers?**	We believe that the proposed project will not cause any ethical implications to the study participants.
**Please state your response to them**	N/A

OBTAINING INFORMED CONSENT

Please note a copy of the explanatory material/information sheet which will be shown to the subjects and the consent form **must be included**.

Consent Form and Participant's Information Sheet have been attached.

**Who will explain the project to the potential participants?**	Research Physiotherapist
**Is there a special relationship between the person explaining the project, or any of the investigators, and the participants?**	No
**When will the explanation be given?**	Prior to data collection.
**Will the participants be able to give consent themselves?**	☐ Yes (✓) No
**If not, why? To whom will the project be explained and who will give consent?**	Children with CP less than 5 years of age will not be able to give consent and therefore, written informed consent will be sought from primary caregivers.
**Will written consent be obtained from all participants?**	(✓) Yes ☐ No
**If not, please give reasons?**	
**Who will act as a witness?**	Parents of other children with CP

References:

1. Khandaker G, Muhit M, Karim T, Smithers-Sheedy H, Novak I, Jones C, et al. Epidemiology of cerebral palsy in Bangladesh: a population-based surveillance study. Dev Med Child Neurol. 2018;61(5):601-9.

2. MIX [Internet]. Washington DC: Microfinance Information Exchange; C2014 [cited 2019 May 27]. Available from: http://www.themix.org

3. BRAC. An end in sight for ultra-poverty: Scaling up BRAC's graduation model for the poorest. Briefing note 1: Ending extreme poverty. Dhaka: BRAC; 2013. p.1-8. Available from: http://www.brac.net/sites/default/files/BRAC%20Briefing%20-%20TUP.pdf

4. Donner A, Klar N. Design and analysis of cluster randomization trials in health research. Hoboken, NJ, USA: John Wiley & Sons, Inc; 2010.

5. Tsoi WS, Zhang LA, Wang WY, Tsang SK, Lo SK. Improving quality of life of children with cerebral palsy: a systematic review of clinical trials. Child Care Health Dev. 2012;38(1): 21-31.

6. Government of Bangladesh (GOB). Annual Report 2011-2012. Dhaka: Local Government Division, Ministry of Local Government, Rural Development and Cooperatives; 2013.

7. Government of Bangladesh (GOB). HEALTH BULLETIN 2016. Dhaka: Ministry of Health and Family Welfare; 2016. Available from: https://dghs.gov.bd/images/docs/Publicaations/HB%202016%20_2nd_edition_13_01_17.pdf

8. Hossain MK. Measuring the impact of BRAC microfinance operations: A case study of a village. Int Bus Res. 2012;5(4):112-23. doi:10.5539/ibr.v5n4p112

9. Mastos M, Miller K, Eliasson AC, Imms C. Goal-directed training: linking theories of treatment to clinical practice for improved functional activities in daily life. Clin Rehabil. 2007;21(1):47-55.

10. Sakzewski L, Ziviani J, Boyd RN. Efficacy of upper limb therapies for unilateral cerebral palsy: a meta-analysis. Pediatr. 2014;133(1):e175-204.

11. Zuurmond M, O'Banion D, Gladstone M, Carsamar S, Kerac M, Baltussen M, et al. Evaluating the impact of a community-based parent training programme for children with cerebral palsy in Ghana. PLoS One. 2018;13(9):e0202096.

CONSENT FOR INCLUSION IN THE STUDY TITLED;

Supporting Ultra-Poor People with Rehabilitation and Therapy - a randomized controlled trial among families of children with Cerebral Palsy in rural Bangladesh (SUPPORT CP trial)

I, [ ] (please print name)

hereby give consent to the inclusion of

[ ] (Child's full name)

in the SUPPORT CP trial, being, primary caregiver responsible (please circle the appropriate response here and throughout this document).

I have read and understood the information sheet and had any questions answered to my satisfaction. I understand that my and my child's participation are fully voluntary in nature and I have the right to withdraw both of us from the study anytime during the study period. I am aware that I should retain a copy of the consent form (when completed) and the information sheet for my records.

I consent to:

Yes	□	No	□	The collection, recording and permanent storage of information relating to me/my child in the SUPPORT CP trial
Yes	□	No	□	Share de-identified information with Cerebral Palsy Alliance Research Institute
Yes	□	No	□	Receive invitations from time to time from CSF Global project staff to participate in future research studies.

Signed [ ] Dated ____/____/ ________

Relationship to the child [ ]

Use only if discussed with an education professional

I, being an education professional certify that, I have explained the project to the adolescent/ primary caregiver and/ or person responsible and consider that he/ she understands what is involved and has freely given his/her consent.

Signed [ ] Dated __ __ /____/ ________

Name [ ] Title [ ]

Participant Information Sheet

Project Title:

Supporting Ultra-Poor People with Rehabilitation and Therapy - a randomized controlled trial among families of children with Cerebral Palsy in rural Bangladesh (SUPPORT CP trial)

Purpose and Objective of the study:

Poverty is a key contributor to late diagnosis and limited access to early intervention and rehabilitation for children with CP in rural Bangladesh. 97% families of children with CP in the country live below the poverty line. Therefore, in low- and middle-income countries, efforts to improve outcomes for children with CP (including quality of life, motor, cognitive and nutritional attainments) should also include measures to improve family economic/social capital. We propose a cluster randomized control trial to evaluate the outcome of an Integrated Microfinance/livelihood and Community-Based Rehabilitation (IMCBR) program for ultra-poor families of children with CP in rural Bangladesh. We predict that this IMCBR will facilitate improved access to capital leading to better income and thus increase the family's investment in physical health overall. Moreover, community-based rehabilitation (CBR) will provide an opportunity for sharing ideas, information, and developing important non-cognitive skills, such as self-confidence of primary caregivers.

The aim of the study is to investigate the outcome of the new integrated intervention (i.e. IMCBR) for ultra-poor families of children with CP in rural Bangladesh in improving health of children with CP and economic/social capital of their families.

Procedure:

Participants randomized to Arm-A (IMCBR) of the trial will attend weekly group sessions for the 12-month duration of the study. During these sessions, they will discuss livelihood activities (e.g. utilization of given livelihood commodity, income, troubleshooting, etc.) and community-based rehabilitation (where education on cerebral palsy management and basic physiotherapy techniques will be taught). Participants randomly selected for Arm-B (CBR alone) will receive community-based rehabilitation only, similar to Arm-A, weekly for 12-month. Participants randomized to Arm-C (care-as-usual i.e. no intervention) will be provided with basic education on early intervention and rehabilitation and will be encouraged to access healthcare via usual routes. Study outcomes will be assessed at baseline, and at 6, 12 and 18 months after randomization.

Potential risks:

There are no known or anticipated risks to you by participating in this research.

Confidentiality:

- Your participation is completely based on your willingness to participate, you are free to withdraw from the research project anytime and withdrawal will not affect you in any way. The research investigator will undertake to safeguard the confidentiality of your response. We will not associate your name with anything you say to your personal information when the data will be published, used a part of a thesis or presented at a conference. Consent forms will be stored separately from the data collected.
- All the data will be stored in password-protected computer and the file will also be password protected. Hard copies will be stored in a locked cabinet. Data will be stored for 6 years after submission of the final report. All the data will be deleted from the computer and hard copies will be shredded.

Right to Withdraw:

- Your participation is voluntary, and you can answer only those questions that you are comfortable with. You may withdraw from the research project for any reason, at any time without explanation or penalty of any sort.

Questions:

- Contact the following designated researcher of this study using the information given below, if you have any questions regarding your rights as a participant.

Name: Mahmudul Hassan Al Imam

Designation: Research Physiotherapist

Detail Address: CSF Global, Flat A-5 & B-3, House 9, Road 2/1, Banani R/A, Dhaka 1213, Bangladesh

Mobile: 01762032227 Telephone (Off.): +88 02 9855731

e-mail: physiomahmud@yahoo.com
